# 2,5-Dihydroxy-1,4-benzoquinones Appended with −P(=O)(OR)_2_ (R = Me or Et) Groups and Their Ammonium and Lithium Salts: Structural, Spectroscopic, and Electrochemical Properties

**DOI:** 10.1021/acsomega.5c07399

**Published:** 2025-10-31

**Authors:** Claire A. Kearney, Kailin M. Mooney, Jordan N. Sanders, Kai J. Edison, Milan H. Hague, S. Joseph Lippert, Timothy J. Dobson, Edward J. Valente, Eugenijus Urnezius

**Affiliations:** Department of Chemistry & Biochemistry, 250672 University of Portland, 5000 N. Willamette Blvd, Portland, Oregon 97203, United States

## Abstract

Synthetic methods yielding 2,5-dihydroxy-1,4-benzoquinones appended with dialkylphosphonato groups were established. Reactions of 1,4-dichloro-2,5-dihydroxybenzene with dialkylchlorophosphates ClP­(=O)­(OR)_2_ (R = Me or Et) produced 1,4-dichloro-2,5-bis­(dimethylphosphato)­benzene (**1a**) and 1,4-dichloro-2,5-bis­(diethylphosphato)­benzene (**1b**). Low-temperature reactions of **1a**–**b** with lithium diisopropylamide (2 equiv) proceeded with double anionic phospho-Fries rearrangement, yielding 2,5-dichloro-3,6-bis­(dialkylphosphonato)-1,4-hydroquinones **2a**–**b**. Oxidations of **2a**–**b** with K_2_S_2_O_8_ under basic conditions proceeded with the nucleophilic displacement of both chlorides by the hydroxy groups. Acidification of the reaction mixtures led to the isolation of 2,5-dihydroxy-1,4-benzoquinones appended with two dimethylphosphonato (H_2_ **3a**) or diethylphosphonato (H_2_ **3b**) groups. Reactions of H_2_ **3a** and H_2_ **3b** with excess NH_3_(aq) or with LiOH yielded salts (NH_4_)_2_ **3a**, (NH_4_)_2_ **3b**, Li_2_ **3a**, and Li_2_ **3b**. All compounds were characterized by spectroscopic (FT-IR, FT-NMR (^1^H, ^13^C, and ^31^P)) methods and by high-resolution mass spectrometry or elemental analyses. Both 2,5-dihydroxy-1,4-benzoquinones H_2_ **3a**–**b** and their ammonium and lithium salts were investigated by cyclic voltammetry. Compounds **2a**, H_2_ **3a**, H_2_ **3b**, and salts (NH_4_)_2_ **3a**·H_2_O, (NH_4_)_2_ **3b**·2H_2_O, Li_2_ **3a**·2H_2_O, and Li_2_ **3b** were characterized by single-crystal X-ray diffraction methods. Compounds H_2_ **3a**/H_2_ **3b** and their salts represent the first examples of anillic acids appended with −P­(=O)­OR_2_ groups. Phosphonato functionalities were shown to be important factors in increasing the solubility of free acids as well as providing coordination/hydrogen bonding sites in their salts.

## Graphical abstract

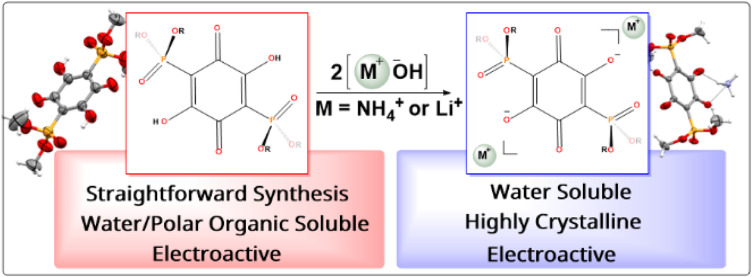

## Introduction

Derivatives of 2,5-dihydroxy-1,4-benzoquinones (Figure 1, **A**) have long attracted the attention of researchers from various subfields of science. They have been recognized for their relatively low p*K*_a_ values and are thus often referred to as anilic acids.^1^ Anilate (2,5-dioxy-1,4-benzoquinonate) dianions are distinguished by two [O,O] chelating sites, which have rendered their extensive usage as bridging ligands in coordination chemistry.^2–4^ The presence of the redox-active quinone moiety also enables anilic acids to play a role in proton/electron transfer events, which have found usage in developing organic materials with novel electric and magnetic properties.^5–10^ More recently, various derivatives of 2,5-dihydroxy-1,4-benzoquinone have been incorporated into the design of components for electric batteries.^11–19^ In order to make further progress in these areas, there is a need for substituted quinone derivatives that are soluble in water and/or in organic solvents, stable to chemical decomposition, and display redox potentials in a specific range.^20–22^ For compounds with the 2,5-dihydroxy-1,4-benzoquinone core, some of these properties can be tuned by placing additional substituents at the remaining positions on the quinone ring (Figure 1, X). However, syntheses of such molecules are often nontrivial, and only about 20 such compounds are known.^3^

**Figure 1:**
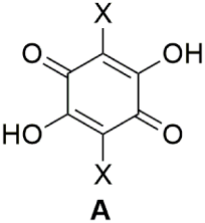
General structural formula of anilic acid (X = halogen, cyano, nitro, alkyl, or aryl.

Our group has been investigating various derivatives of 1,4-benzoquinones appended with two phosphoranyl −P­(=O)­R_2_ groups. We have previously shown that 2,5-bis­(diisopropylphosphoranyl)-3,6-dihydroxy-1,4-benzoquinone can be formed via oxidative dehalogenation when 2,5-bis­(diisopropylphosphoranyl)-3,6-difluoro-1,4-hydroquinone is reacted with selected Cu­(II)-2,2’-bipyridyl complexes.^23^ The presence of phosphoranyl groups expanded the anilate ligand functionality, with the oxygen atom from P=O moiety serving as either a donor site for metal coordination or as an H-bond acceptor in a hydrogen-bonding network.^23^ This led us to pursue a broader study where the oxidation of 2,5-bis­(−P­(=O)­R_2_))-3,6-dihalo-1,4-hydroquinones was followed by reaction with selected nucleophiles. Following this methodology, we obtained 2,5-dihydroxy-1,4-benzoquinones appended with two −P­(=O)­Ph_2_ or −P­(=O)*i*Pr_2_ groups, as well as developed a new synthetic method toward 2,5-bis­(phosphoranyl)-3,6-diaminoquinones.^24,25^ While studying anilic acids appended with two −P­(=O)­R_2_ groups, we noted that these compounds display strong intramolecular OH···O=P- bonding which in turn affects their redox-potentials, solid-state structures, and solubilities.^24^ They readily dissolved in polar organic solvents (THF, CH_3_CN, DMF, CH_2_Cl_2_, CHCl_3_, CH_3_OH, and (CH_3_)_2_C=O) but were not soluble in water at neutral pH. In theory, the solubility properties of 2,5-dihydroxy-3,6-bis­(−P­(=O)­R_2_)-1,4-benzoquinones could be tuned by varying alkyl/aryl substituents on phosphorus atoms in −P­(=O)­R_2_ groups, but such an approach is hindered by the limited commercial availability of chlorophosphines ClPR_2_. Therefore, we considered the syntheses of 2,5-dihydroxy-1,4-benzoquinones appended with phosphonato (-P­(=O)­(OR)_2_) groups (Figure 2, H_2_ **3a** and H_2_3**b**). We reasoned that more hydrophilic phosphonato functionalities might yield anilic acid derivatives featuring higher solubility in water while retaining some of their solubility in organic solvents. Furthermore, phosphoester linkages in -P­(=O)­(OR)_2_ can be subjected to hydrolysis to yield the phosphonic acid moiety −P­(=O)­(OH)_2_.^26^ Attaching such groups to electroactive organic compounds via hydrocarbon linkages has been implemented as an effective method to tune their solubility in aqueous media.^27,28^ Positioning phosphonato groups directly onto the 2,5-dihydroxy-1,4-benzoquinone moiety may also be a tool for adjusting its electronic properties, as we have observed −P­(=O)­R_2_ substituents to lower the redox potential of 2,5-bis­(phosphoranyl)-3,6-diaminoquinones.^25^ With this in mind, we proceeded to synthesize two new 2,5-dihydroxy-1,4-benzoquinones appended with −P­(=O)­(OR)_2_ groups (H_2_ **3a** and H_2_ **3b**). These compounds readily react with bases, forming water-soluble salts containing dianions **3a** and **3b**. Ammonium and lithium salts (Figure 2) have been isolated and fully characterized, with the results reported here.

**Figure 2:**
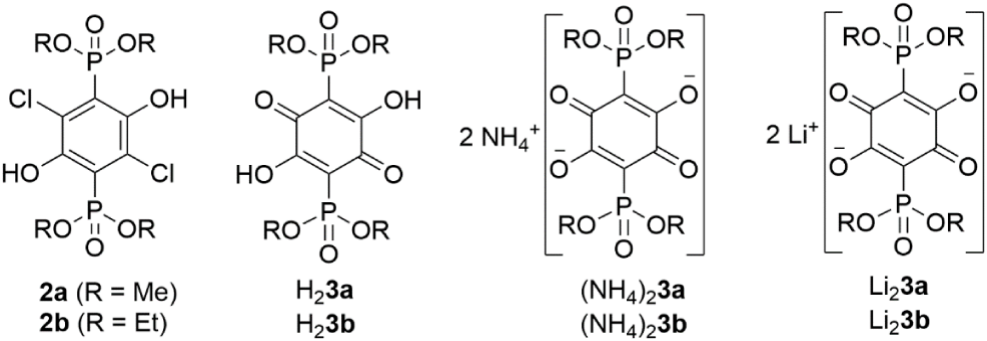
Compounds synthesized and investigated in this work.

## Results and Discussion

### Syntheses

Reactions of 1,4-dichloro-2,5-dihydroxybenzene with chlorophosphate ClP­(=O)­(OR)_2_ produced 1,4-bis­(dialkoxyphosphato)-2,5-dichlorobenzenes **1a**–**b**, as depicted in Scheme 1 (step 1). Compound **1a** was isolated as a crystalline solid, and **1b** was obtained in the form of viscous oil. Their ^31^P NMR signals (singlets at −4.8 ppm (**1a**) and −7.0 ppm (**1b**) matched well with data reported for other structurally related compounds.^26^

**Scheme 1:**
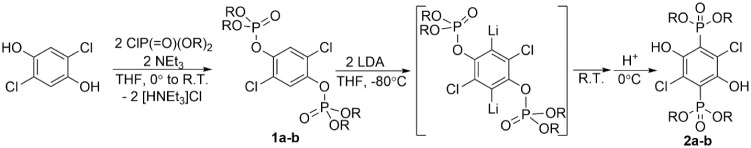
Syntheses of 1,4-Dichloro-2,5-bis­(dialkylphosphato)­benzenes **1a** (R = Me) and **1b** (R = Et) and 2,5-Dichloro-3,6-bis­(dialkylphosphonato)-1,4-hydroquinones **2a**–**b**

Low-temperature reactions of **1a**–**b** with LDA proceeded with deprotonation of the aromatic C–H sites, which then led to a double anionic phospha-Fries rearrangement (steps 2 and 3, Scheme 1).^29^ This is a well-established synthetic methodology for introducing phosphonato groups onto hydroquinone or phenol cores.^26,30,31^ The final steps in our syntheses included acidification of the reaction media, followed by extraction and crystallization, thus yielding 2,5-dichloro-3,6-bis­(dialkylphosphonato)-1,4-hydroquinones **2a**–**b** (Scheme 1). Both compounds were isolated as crystalline solids and were fully characterized, including single-crystal X-ray diffraction characterization of **2a**. Chemical shift values in ^31^P NMR spectra of **2a** (20.5 ppm) and **2b** (17.5 ppm) in CDCl_3_ were shifted upfield when compared to analogous signals reported for 2,5-bis­(dimethylphosphonato)-1,4-hydroquinone (25.09 ppm) and 2,5-bis­(diethylphosphonato)-1,4-hydroquinone (22.32 ppm) reported in the same solvent.^32^ We attribute this increase in chemical shielding to contributions of electron density from chlorine atomic orbitals to HOMO of **2a**–**b**, similar to what we reported for the role of fluorine in 2,5-difluoro-3,6-bis­(diphenylphosphoranyl)-1,4-hydroquinone.^33^ We also observed that solutions of both **2a** and **2b** strongly emit in the blue range of the visible spectrum when subjected to illumination by UV light. This is consistent with our earlier report, where analogous observations were made about the properties of 2,5-dichloro-1,4-hydroquinones appended with −P­(=O)­R_2_ (R = Ph or *i*Pr) groups.^24,25^ All of these compounds are currently being investigated in our broader collaborative study on various 1,4-hydroquinones appended with phosphoryl (−P­(=O)­R_2_) or phosphonato (−P­(=O)­(OR)_2_) groups as single benzene fluorophores.^34,35^

Subjecting compounds **2a**–**b** to an excess of base and a strong oxidizing agent led to the oxidative dehalogenation of the dichlorohydroquinone cores, yielding derivatives of new anilic acids (Scheme 2). This process follows our methodology established earlier for the conversion of 2,5-diphosphoranyl-3,6-dichloro-1,4-hydroquinones into anilic acids appended with −P­(=O)­R_2_ groups.^24^ Most of the procedures depicted in Scheme 2 were carried out in water, which is a notable difference from the conditions used in our earlier work.^24^ While the starting hydroquinones **2a**–**b** display very limited solubility in water, their salts formed after the addition of MOH (M = Na or K) dissolved readily. Reactions of these salts with persulfate presumably yield the dichloroquinones shown in square brackets (Scheme 2, step 1), but all of our attempts to isolate these intermediates were unsuccessful.

**Scheme 2:**
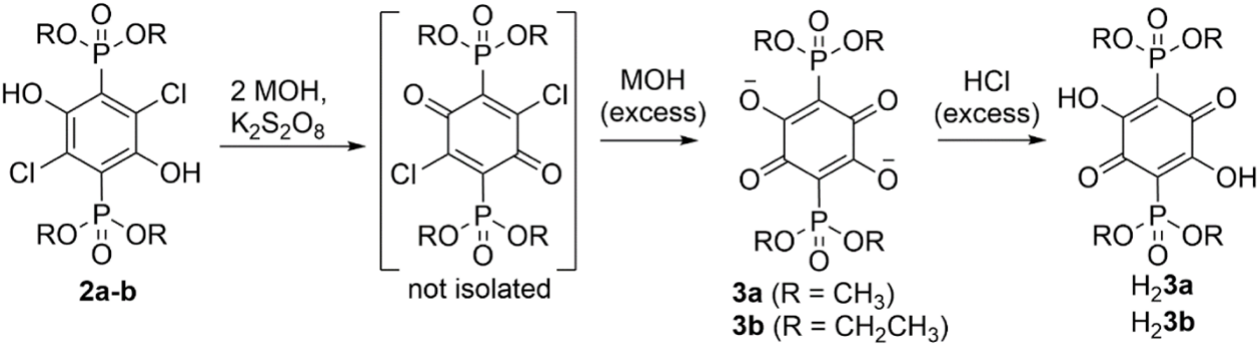
Syntheses of 2,5-Dihydroxy-1,4-benzoquinones Appended with −P­(=O)­(OR)_2_ Groups

When reactions were conducted with excess of KOH, we observed gradual (over 20–25 min) disappearances of ^31^P NMR signals attributed to deprotonated hydroquinones **2a** (17.1 ppm) and **2b** (16.1 ppm), which were accompanied by the rise of new signals at 28.0 and 25.2 ppm. The latter signals were attributed to potassium salts of dianions **3a**–**b**. After about half an hour, the reaction mixture consisted of a homogeneous yellow solution containing the corresponding anilate salt as well as dissolved sulfate, chloride, and the remaining base. Attempted separations of these components via fractional crystallization were not successful, as mixtures of salts were deposited upon slow (weeks-long) evaporation of water. Separation of organic/inorganic components was accomplished when these isolated solid mixtures were suspended in acetonitrile, followed by the addition of a slight excess of concentrated aqueous HCl. Under these conditions, dianions **3a**–**b** were protonated, and the anilic acids formed (H_2_ **3a/**H_2_ **3b**) dissolved in CH_3_CN/H_2_O (∼4:1) mixture. Stirring this suspension for ∼10–15 min led to the formation of a deep yellow solution above a white crystalline solid (presumably a mixture of potassium sulfate/chloride). The solids were separated by filtration, volatiles were removed, and recrystallization yielded compounds H_2_ **3a**–H_2_ **3b** as orange crystalline materials. Both compounds were fully characterized, including single-crystal X-ray structural work. To the best of our knowledge, these compounds are the first examples of anilic acids appended with phosphonato substituents. They are soluble in polar organic solvents (THF, CH_3_CN, CH_2_Cl_2_, CHCl_3_, and CH_3_OH) but have limited solubilities in toluene or benzene. Furthermore, both H_2_ **3a** and H_2_ **3b** are also soluble in water. According to our initial measurements, H_2_ **3a** is sufficiently soluble to form up to a 0.6 M aqueous solution at room temperature (23.0–23.5 °C), whereas the solubility of H_2_ **3b** is lower (∼0.1 M). For comparison, the parent compound, 2,5-dihydroxy-1,4-benzoquinone, was reported to form ∼3 mM aqueous solutions at room temperature.^36^

Analysis of ^1^H, ^13^C, and ^31^P NMR spectra of H_2_ **3a** and H_2_ **3b** sheds more light on the behavior of anilic acid functionality in solutions. Broad signals for OH protons (H_2_ **3a** δ ∼ 11.3–10.2 (br); H_2_ **3b** δ ∼ 12.3–10.8 (br)) were observed in their ^1^H spectra recorded in CDCl_3_. Corresponding signals were not present in D_2_O, which can be attributed to fast H^+^/D^+^ exchange occurring between the anilic acid and the solvent. This process also affected ^13^C NMR signals of oxygen-bound carbon atoms in the 2,5-dihydroxy-1,4-benzoquinone cores of H_2_ **3a** and H_2_ **3b**. Anilic acid moieties of both compounds showed only two signals in ^13^C spectra recorded in D_2_O (H_2_ **3a**: δ 176.1 (m, (br)); 97.7 (d, ^1^J_PC_ = 183 Hz); H_2_ **3b**: δ 175.8 (m, (br)), 99.9–98.0 (m)). This is consistent with fast proton transfer to the solvent, which renders −C–O^–^ and C=O carbon atoms of the anilic acid equivalent by resonance. We have observed similar patterns in ^13^C NMR for 2,5-dihydroxy-1,4-benzoquinones appended with two −P­(=O)­R_2_ groups.^24^ Due to the limited solubility of H_2_ **3a** in CDCl_3_ signals of oxygen-bound carbon atoms from the anilic acid core were not observed in ^13^C NMR. Compound H_2_ **3b** was more soluble, and the ^13^C NMR spectrum of a saturated solution showed two broad but distinct signals at 177.5 and 167.5 ppm, which we assigned to C=O and C–OH carbon atoms of the anilic acid unit (Supporting Information, Figure S20). Differences between the ^31^P NMR spectra of H_2_ **3a** and H_2_ **3b** in CDCl_3_ and D_2_O were less pronounced. In CDCl_3_, sharp singlets at 20.7 (H_2_ **3a**) and 17.7 (H_2_ **3b**) ppm were observed, whereas the corresponding signals in D_2_O were broader, centered at 22.6 and 18.9 ppm, respectively. Such changes can be attributed to differences in hydrogen bonding available for P=O groups in these two solvents.^37^

When aqueous solutions of acids H_2_ **3a** and H_2_ **3b** were treated with two equiv of base (ammonia or alkali metal hydroxide), the corresponding anilate salts were readily formed. The work presented herein is limited to our investigations of the ammonium and lithium salts (NH_4_)_2_ **3a**/(NH_4_)_2_ **3b** and Li_2_ **3a**/Li_2_ **3b** (Scheme 3). They were isolated in the crystalline state by slow evaporation of the solvent. All four compounds are soluble in water. According to our preliminary estimations, ammonium salts were substantially more soluble, with both (NH_4_)_2_ **3a** and (NH_4_)_2_ **3b** able to form up to 1.0 M aqueous solutions at 23.0–23.5 °C. Under the same conditions, Li_2_ **3a** was soluble enough to form ∼0.15 M solution, whereas Li_2_ **3b** was the least soluble (up to 0.07 M). Selected organic solvents (THF, CH_3_OH, and CH_3_CN) did not yield solutions of these salts that could be used for practical purposes (spectroscopy or cyclic voltammetry).

**Scheme 3:**
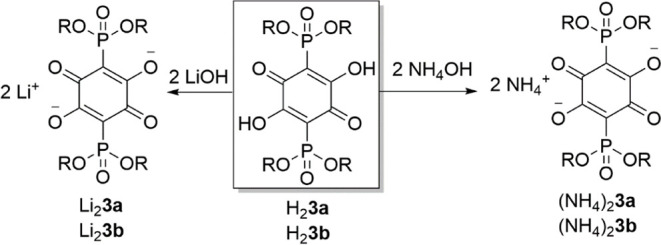
Syntheses of (NH_4_)_2_ **3a**/(NH_4_)_2_ **3b** and Li_2_ **3a**/Li_2_ **3b**

### Structural Studies

Compounds **2a**, H_2_ **3a**, H_2_ **3b**, and salts (NH_4_)_2_ **3a**/(NH_4_)_2_ **3b** and Li_2_ **3a**/Li_2_ **3b** have been characterized by single-crystal X-ray diffraction methods. General crystallographic parameters are compiled in Table S1 included in Supporting Information. Thermal ellipsoid plots for **2a**, H_2_ **3a**, and H_2_ **3b** are shown in Figure 3.

**Figure 3:**
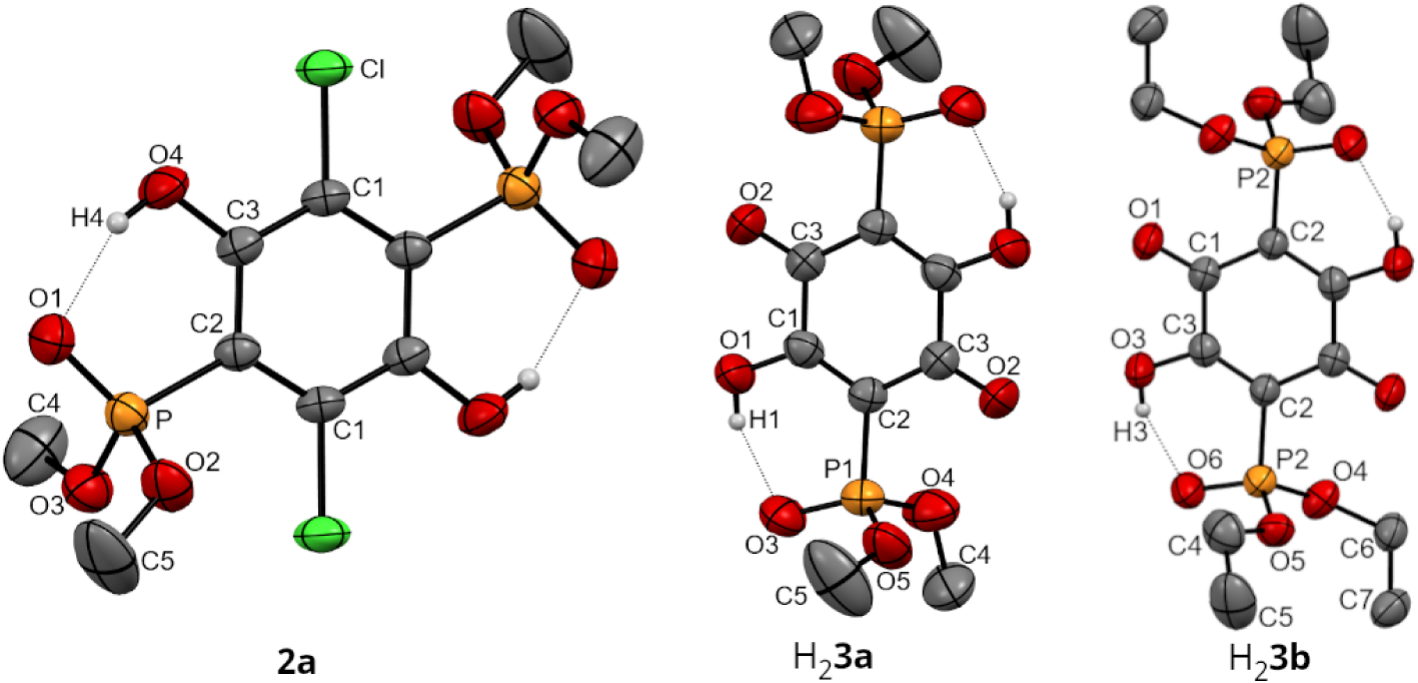
Thermal ellipsoid plots (probability level 50%) of **2a**, H_2_ **3a** (one of the two independent molecules in the unit cell), and H_2_ **3b**. Hydrogen atoms of the C–H bonds have been omitted for the sake of clarity.

Crystals suitable for single-crystal X-ray diffraction experiments were obtained from dichloromethane (**2a**) or acetone (H_2_ **3a** and H_2_ **3b**). All three compounds feature intramolecular P=O···H–O hydrogen bonds (**2a**: O1–H4, 1.817 Å; H_2_ **3a**: O3–H1, 1.781 Å and O8–H6, 1.954 Å; H_2_ **3b**: O6–H3, 1.796 Å). This is consistent with our earlier report, where such interactions were determined for structurally similar 2,5-difluoro-3,6-bis­(P­(=O)­R_2_)-1,4-hydroquinones^33^ (R = Ph or ^*i*^Pr). Intramolecular hydrogen bonding in **2a** and H_2_ **3a**/H_2_ **3b** is limited to P=O functionality only, with no interactions detected between the oxygen atoms of the phosphoester groups in any of the three compounds detected. Other bond lengths and angles determined for **2a,** H_2_ **3a**, and H_2_ **3b** (Table 1) are not exceptional.

**Table 1:** Selected Bond Lengths and Angles for **2a,** H_2_ **3a,** and H_2_ **3b**

Selected bond lengths (Å) and angles (◦)					
**2a**		H_2_ **3a** ^a^		H_2_ **3b**	
O4–C3	1.3539(17)	O2–C3	1.215(5); (1.212(5))	O1–C1	1.214(3)
O1–P	1.4765(13)	O1–C1	1.353(6); (1.331(5))	O3–C3	1.315(3)
P–C2	1.8032(14)	P1–C2	1.774(4); (1.777(4))	P2–C2	1.791(3)
C1–C2	1.4037(19)	O3–P1	1.471(4); (1.473(4))	O6–P2	1.481(2)
C2–C3	1.4120(19)	C1–C3	1.503(6); (1.517(6))	C1–C3	1.509(4)
C1–C3	1.400(2)	C1–C2	1.353(6); (1.345(6))	C1–C2	1.463(4)
C1–Cl	1.7332(15)	C2–C3	1.438(6); (1.440(6))	C3–C2	1.356(4)
O4–C3–C2	123.98(13)	O2–C3–C1	118.3(4); (119.4(4))	O1–C1–C2	123.1(2)
O4–C3–C1	117.16(12)	O1–C1–C3	112.7(4); (111.6(4))	O1–C1–C3	119.2(2)
P–C2–C3	118.83(10)	O2–C3–C2	123.2(4); (122.5(4))	O3–C3–C1	112.1(2)
P–C2–C1	122.46(11)	P1–C2–C1	119.2(3); (119.6(3))	O3–C3–C2	125.7(3)
C1–C3–C2	118.86(12)	C3–C1–C2	121.7(4); (121.7(4))	C2–C1–C3	117.7(2)
C3–C2–C1	118.71(13)	C1–C2–C3	119.8(4); (120.1(4))	C1–C3–C2	122.2(2)
		P1–C2–C3	120.9(3); (120.3(3))	P2–C2–C3	119.8(2)

a Two independent molecules of H_2_ **3a** are present in the unit cell; the values of structurally equivalent parameters for the other molecule are listed in parentheses.

Crystalline samples of ammonium and lithium salts (NH_4_)_2_ **3a**·H_2_O, (NH_4_)_2_ **3b**·2H_2_O, Li_2_ **3a**·2H_2_O, and Li_2_ **3b** were obtained by slow evaporation of their aqueous solutions. A thermal ellipsoid plot representing a unit cell of (NH_4_)_2_ **3a**·H_2_O with extended packing is shown in Figure 4. Two nonequivalent anilates distinguished by P1 and P2 labels of phosphorus atoms are present in the unit cell. Dianions **3a** with P1 atoms can be visualized as forming layers that are held together by hydrogen bonds formed with ammonium cations and water molecules. Hydrogen atoms from H_2_O and NH_4_^+^ are also engaged in interactions with oxygen atoms from other anilates (with the P2 phosphorus atom, Figure 4), thus establishing bridges between layers. Selected bond lengths and angles of the anilate fragments from (NH_4_)_2_ **3a** and (NH_4_)_2_ **3b** are compiled in Table 2. An expanded view of hydrogen bonding surrounding both types of anilates in (NH_4_)_2_ **3a**·H_2_O is shown in Figure 5. The first picture (Figure 5a) depicts a H-bonding network between a pair of anilates belonging to the layer fragment. A notable structural feature seen in this fragment is hydrogen bonding from NH_4_^+^ and H_2_O that involves all the oxygen atoms on dianions **3a** present in the layer substructure. Interactions between N–H protons from the ammonium cations bearing N1 nitrogen with oxygen atoms from the 2,5-dioxy-1,4-quinonate cores of the **3a** (O4–H1AC 2.086 Å, O5–H1AD 2.059 Å) result in the formation of a 14-membered ring. This arrangement is further fortified by additional hydrogen bonds from a pair of diagonally opposed anilate oxygen atoms to ammonium cations (N2) positioned above and below the 14-membered ring (O4–H2AB 1.994 Å, O4–H2AC 1.991 Å). The oxygen atom of the phosphonato (P=O) group is bound to the N–H group of the N1 ammonium cation (O2–H1AD 2.460 Å), and to an −OH group of the water molecule (O2–H1WA 2.044 Å). Oxygen atoms from the phosphoester functions (P­(OCH_3_)_2_) also participate in hydrogen bonding, forming relatively long hydrogen bonds (O3–H1AC 2.708 Å, O1–H1AC 2.602 Å) to one of the N–H groups on the N1 ammonium cation.

**Figure 4:**
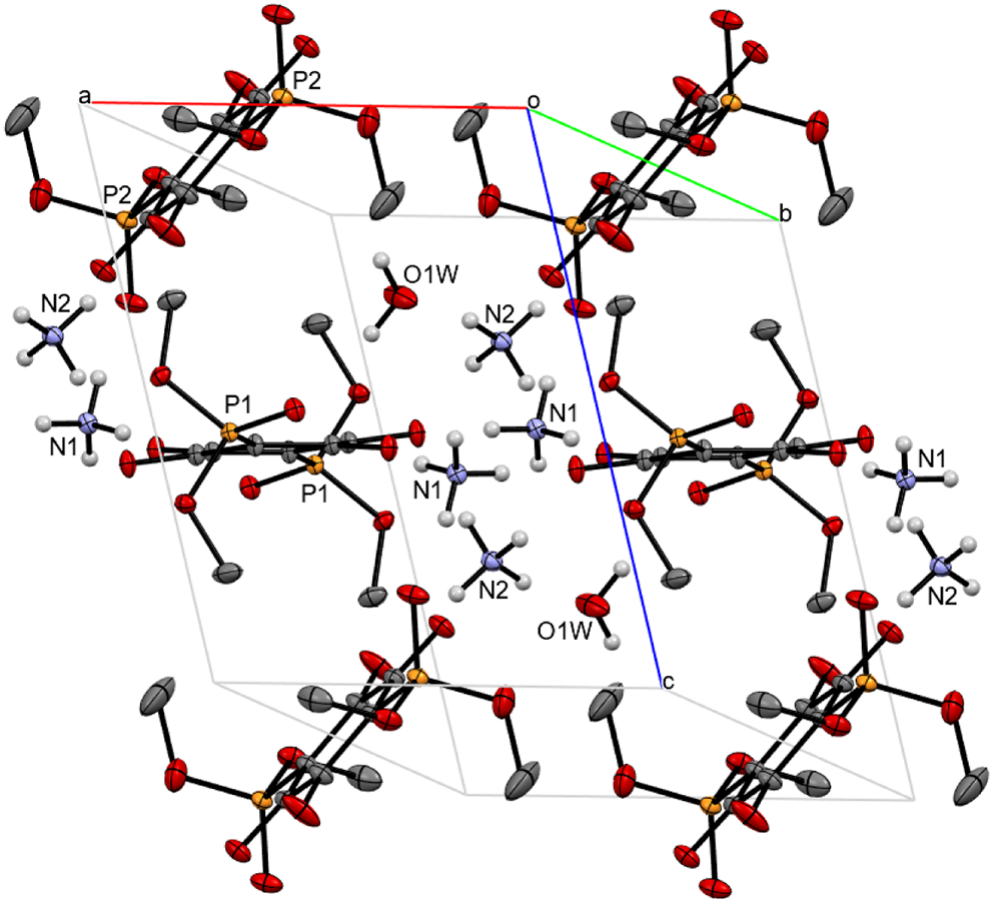
Thermal ellipsoid plot (probability level 50%) of (NH_4_)_2_ **3a**·H_2_O. Hydrogen atoms of the C–H bonds are omitted for clarity.

**Figure 5:**
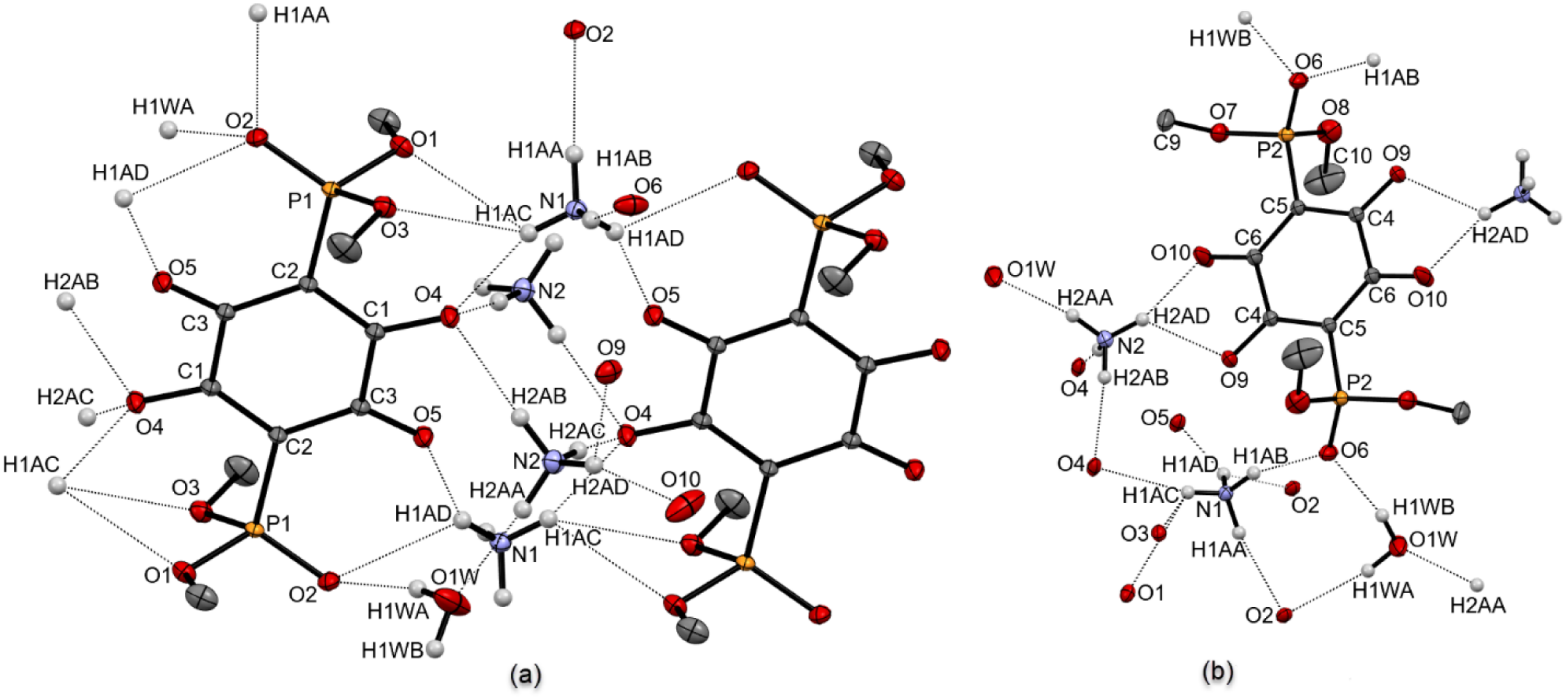
Hydrogen bonding network in crystalline (NH_4_)_2_ **3a**·H_2_O. (a) Fragment of the layer structure. (b) Hydrogen bonding involving bridging anilate. Hydrogen atoms of the C–H bonds are omitted for clarity.

**Table 2:** Selected Bond Lengths and Angles for the Anilate Anions in (NH_4_)_2_ **3a**, (NH_4_)_2_ **3b**, Li_2_ **3a**, and Li_2_ **3b**

Selected bond lengths (Å) and angles (◦)									
(NH_4_)_2_ **3a** (layer)		(NH_4_)_2_ **3a** (bridging)		(NH_4_)_2_ **3b**		Li_2_ **3a**		Li_2_ **3b**	
O5–C3	1.2348(10)	O10–C6	1.2444(12)	O2–C3	1.2374(16)	O2–C1	1.235(3)	C1–O1	1.235(4)
C3–C2	1.4236(12)	C6–C5	1.4131(13)	C3–C4	1.4164(17)	C2–C1	1.423(3)	C1–C2	1.424(5)
C2–C1	1.4043(12)	C5–C4	1.4272(13)	C4–C5	1.4169(18)	C2–C3	1.414(3)	C2–C3	1.397(5)
C1–O4	1.2563(11)	C4–O9	1.2383(11)	C5–O3	1.2356(16)	O3–C3	1.242(3)	C3–O2	1.268(4)
C3–C1	1.5455(12)	C6–C4	1.5503(13) 1.4725(11)	O1–C2	1.2279(16)	C3–C1	1.549(3)	C1–C3	1.535(5)
C2–P1	1.7660(9)	C5–P2	1.7706(10)	C2–C1	1.4208(17)	P1–C2	1.768(2)	C2–P1	1.782(3)
P1–O2	1.4810(7)	P2–O6	1.4789(8)	C1–C6	1.4106(17)	P1–O1	1.4778(17)	P1–O3	1.472(3)
				C6–O4	1.2455(16)				
O4–C1–C2	124.03(8)	O9–C4–C5	123.67(9)	C2–C3	1.5440(17)	C3–C2–C1	120.17(19)	O1–C1–C2	124.2(3)
O4–C1–C3	115.20(7)	O9–C4–C6	115.84(8)	C5–C6	1.5450(17)	O3–C3–C2	126.6(2)	O1–C1–C3	115.2(3)
C1–C2–C3	119.72(8)	C4–C5–C6	120.50(8)	P1–C1	1.7696(13)	O3–C3–C1	114.07(19)	C2–C1–C3	120.6(3)
P1–C2–C3	122.75(6)	P2–C5–C4	124.32(7)	P2–C4	1.7666(13)	O2–C1–C2	124.7(2)	C3–C2–C1	118.5(3)
O2–P1–C2	112.98(4)	O6–P2–C5	114.30(4)	P1–O7	1.4725(11)	O2–C1–C3	115.1(2)	O2–C3–C2	125.2(3)
				P2–O9	1.4683(12)	O1–P1–C2	113.03(10)	O3–P1–C2	111.95(15)
				O2–C3–C4	125.72(12)				
				C3–C4–C5	119.46(11)				
				O3–C5–C4	124.73(12)				
				O1–C2–C1	125.16(12)				
				O1–C2–C3	114.61(11)				

Connectivity between layers in solid (NH_4_)_2_ **3a**·H_2_O is established via hydrogen bonding from ammonium cations and water molecules to bridging anilates. An expanded view of these interactions is shown in Figure 5b. In addition to contributing to bonding in the layer fragment, water and the ammonium ion (N1) also form hydrogen bonds to the phosphonato oxygen atom from the bridging anilate (O6–H1AB 1.915 Å, O6–H1WB 1.931 Å). The other ammonium ion (N2) interacts with the bridging anilate via hydrogen bonds to the oxygens of the 2,5-dioxy-1,4-quinonate (O10–H2AD 1.887 Å, O9–H2AD 2.361 Å). Oxygen atoms in the phosphoester groups (P­(OCH_3_)_2_) on the bridging anilates are not part of the hydrogen bonding network.

The thermal ellipsoid plot capturing a fragment of the structure of (NH_4_)_2_ **3b**·2H_2_O is shown in Figure 6. Both ammonium cations (N1A and N2A) and water molecules (O1W and O2W) are located over two nonequivalent positions. One pair of oxygen atoms from the 2,5-dioxy-1,4-quinonate core (O1 and O2) form hydrogen bonds to the H1WA atom from one of the water molecules (O1–H1WA 2.076 Å, O2–H1WA 2.374 Å), with an additional bond to the ammonium cation formed from O2 (O2–H1AD 1.978 Å).

**Figure 6:**
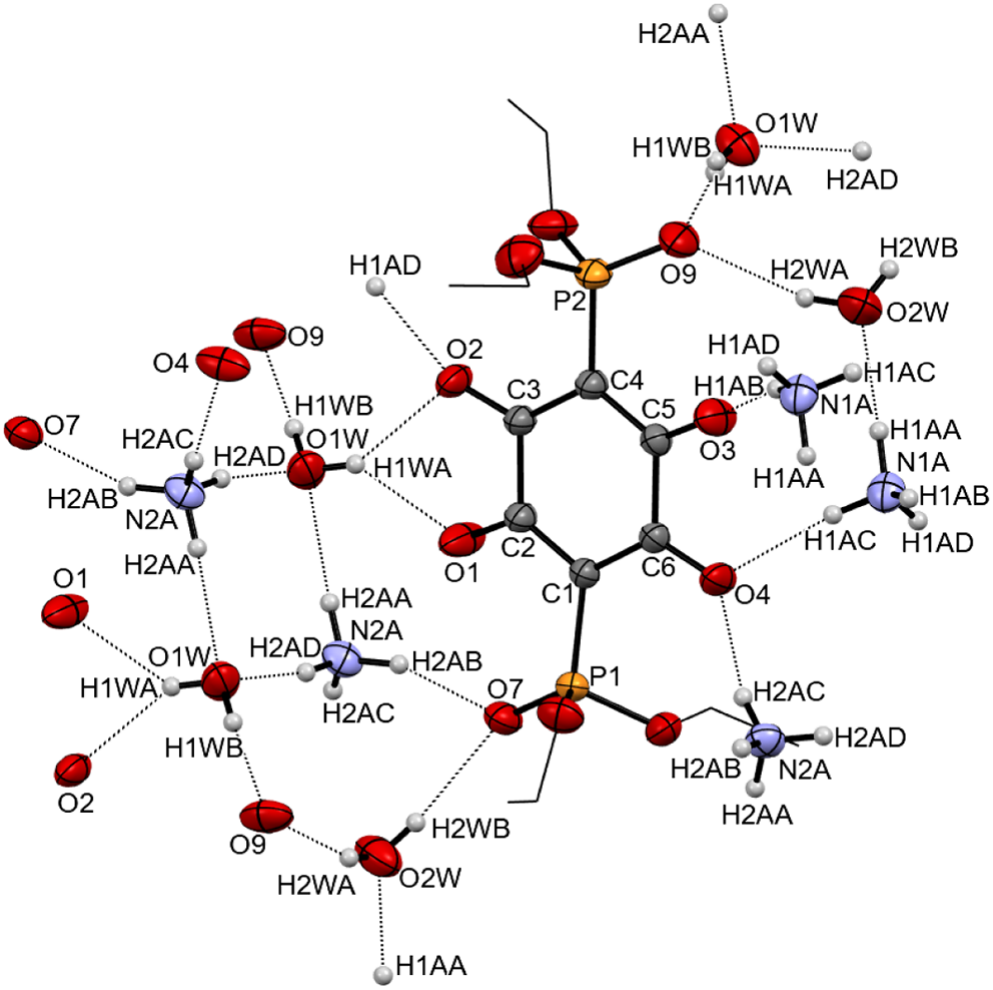
Thermal ellipsoid plot (probability level 50%) showing extended hydrogen bonding in crystals of (NH_4_)_2_ **3b**·2H_2_O. For clarity, hydrogen atoms of the C–H bonds are omitted; ethyl groups are shown in wireframe.

Oxygen atoms on the opposite side of the same 2,5-dioxy-1,4-quinonate core (O3 and O4) are bound exclusively to hydrogens from N–H functionalities (O3–H1AB 2.016 Å, O4–H1AC 2.073 Å, and O4–H2AC 1.996 Å) from the ammonium cations. Other notable interactions include hydrogen bonding to P=O oxygen atoms of the phosphonate groups (O7–H2AB 2.036 Å, O7–H2WB 2.104 Å, O9–H2WA 2.136 Å, and O9–H1WB 1.866 Å).

Crystals of lithium salts Li_2_ **3a**·2H_2_O and Li_2_ **3b** were grown from aqueous solutions upon the slow evaporation of water. A thermal ellipsoid plot representing a fragment of Li_2_ **3a**·2H_2_O is shown in Figure 7. Lithium ions are pentacoordinate, bound into two distinct chelating pockets, both sharing an O3 atom from the dianion **3a**. A five-membered ring is formed with a Li cation coordinating to the O2 and O3 atoms of the anilate ion (Li1–O2 2.104(5) Å, Li1–O3 2.060(5) Å).

**Figure 7:**
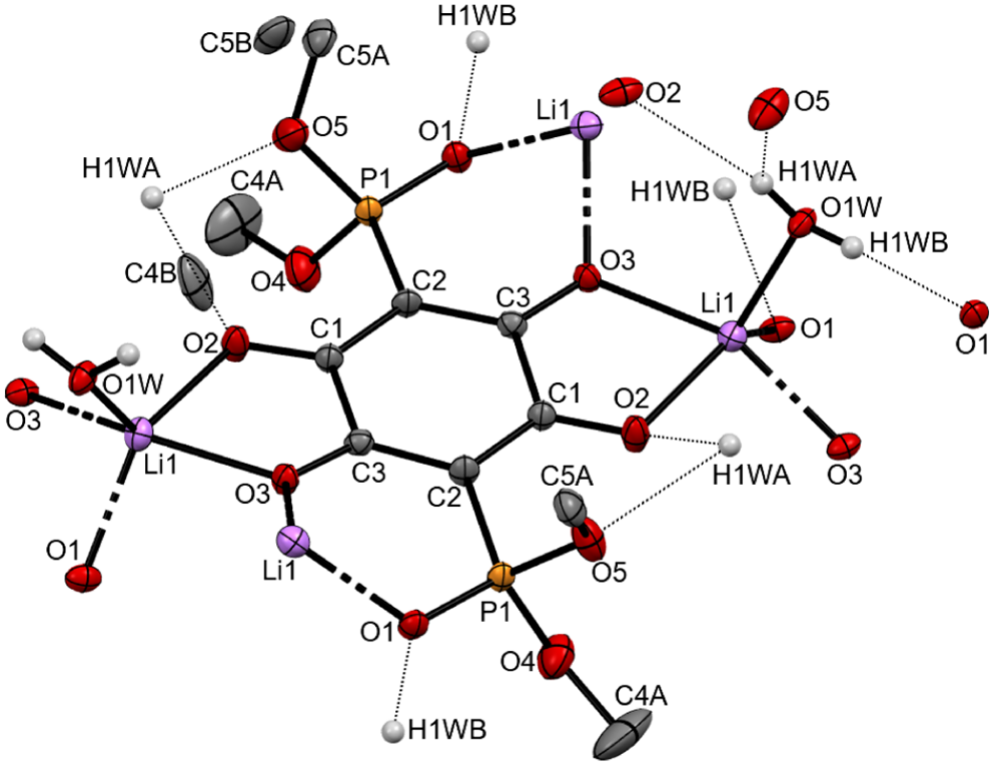
Thermal ellipsoid plot (probability level 50%) of Li_2_ **3a**·2H_2_O. Hydrogen atoms of the C–H bonds have been omitted for clarity. The terminal methyls of both methoxy groups are disordered over two positions. They were modeled with partial occupancy of methyl groups [major conformer 0.506(7)] and involved 44 restraints for C–O distances and ethoxy C,O isotropic librational factors.

Atom O3 is also part of the second chelating pocket [O, (P=O)], where a six-membered ring is formed with Li^+^ also coordinated to the oxygen of the phosphonato group (Li1–O3 2.049(4) Å, Li1–O1 2.006(5) Å). The fifth coordination site on the metal is occupied by a coordinated water molecule (Li1–O1W 1.924(5)­Å). The geometry around the Li center is best described as distorted square pyramidal based on the estimated value of the tau factor for pentacoordinate compounds (τ_5_ = 0.375).^38^ Both hydrogen atoms of the coordinated H_2_O molecule are engaged in hydrogen bonding to various oxygen atoms on phosphonate (H1WB–O1 1.955 Å), anilate (H1WA–O2 2.186 Å), and phosphoester (H1WA–O5 2.339 Å) functionalities on adjacent **3a** moieties. These hydrogen bonds contribute to the assembly of a metal–organic framework structure in crystals of Li_2_ **3a**·2H_2_O. A structural fragment consisting of four dianions **3a** with coordinated lithium cations and water (viewed along the *c* axis) is shown in Figure 8a. The projection resembles a paddle wheel pattern, with lithium centers and coordinated oxygen donors arranged along an “axis”, and each anilate (C_6_O_4_^2–^) plane serving as “paddle”. Continuing buildup around this fragment produces the MOF structure of Li_2_ **3a**·2H_2_O, which resembles a basketweave tile pattern along the *c* axis (Figure 8b). Each “tile” represents a rectangular channel that extends along the *c* axis, where the inner space is partially occupied by −OCH_3_ groups of the phosphonato functionalities.

**Figure 8:**
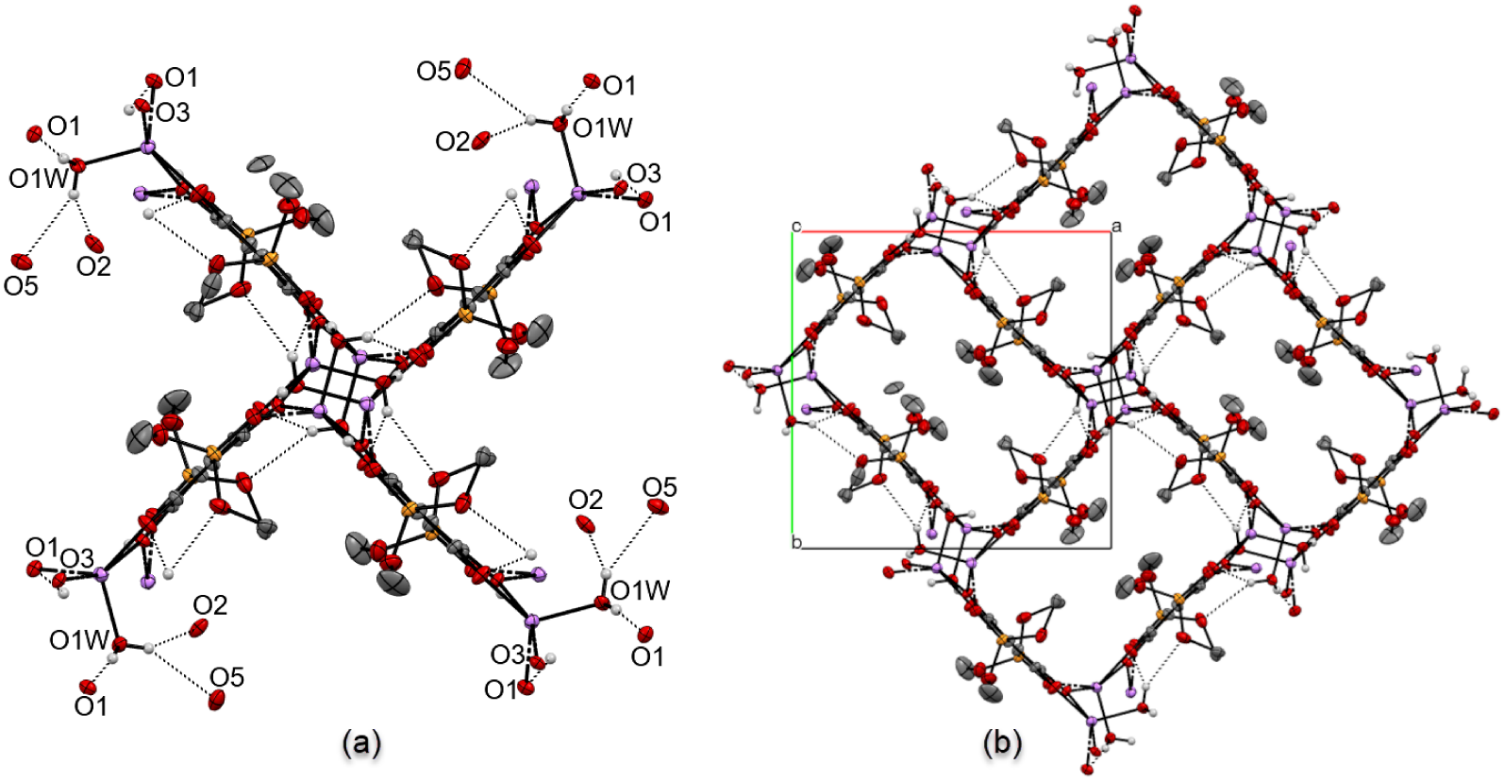
Paddlewheel (a) and basketweave tile (b) patterns in the extended MOF structure of Li_2_ **3a**·2H_2_O. Hydrogen atoms of the C–H bonds are omitted for clarity.

The dimensions of each channel are measured at 6.40 Å × 9.90 Å, based on the distances between planes drawn through C_6_O_4_^2–^ cores on the opposing sides of the channel.

The thermal ellipsoid plot representing a structural fragment of Li_2_ **3b** is shown in Figure 9a. Lithium ions are tetracoordinate, bound to oxygen atoms in [O,O] (Li–O1 1.981(7) Å, Li–O2 1.961(7) Å), and [O, (P=O)] (Li–O2 1.915(7) Å, Li–O3 1.855(6) Å) chelation pockets on the anilate anions **3b**. Even though crystals were grown from an aqueous solution, there is no coordinated water, as seen in Li_2_ **3a**·2H_2_O. The coordination geometry around the metal can be described as distorted tetrahedral, based on the estimated value of the tau factor for tetracoordinate centers (τ_4_ = 0.727).^39^ The buildup of the crystalline lattice in Li_2_ **3b** can be visualized through the expanded fragment shown in Figure 9b. Coordination of lithium cations into [O,O] and [O, (P=O)] pockets of adjacent anilate units results in an almost orthogonal arrangement of the connected C_6_O_4_^2–^ planes (Figure 9b; the angle between the horizontal plane (shown in dark gray) and the vertical plane (plane of the paper) is measured at 89.04°). Expansion of this fragment produces a two-dimensional layer. In the extended structure of crystalline Li_2_ **3b** (packing diagram along the *b* axis shown in Figure 10a), such layers are stacked, with the ethoxy groups from the phosphonate functionalities pointing into the space between the layers. Another noteworthy feature of Li_2_ **3b** is the presence of rhomboid channels. These channels are revealed when a projection of the packing diagram along the *c* axis is additionally rotated by 20° along the vertical axis (Figure 10b). The size of the channel (wall to wall) is estimated at 3.05 Å × 3.78 Å, based on the distances between planes drawn through C_6_O_4_^2–^ cores constituting opposing sides of the channel.

**Figure 9:**
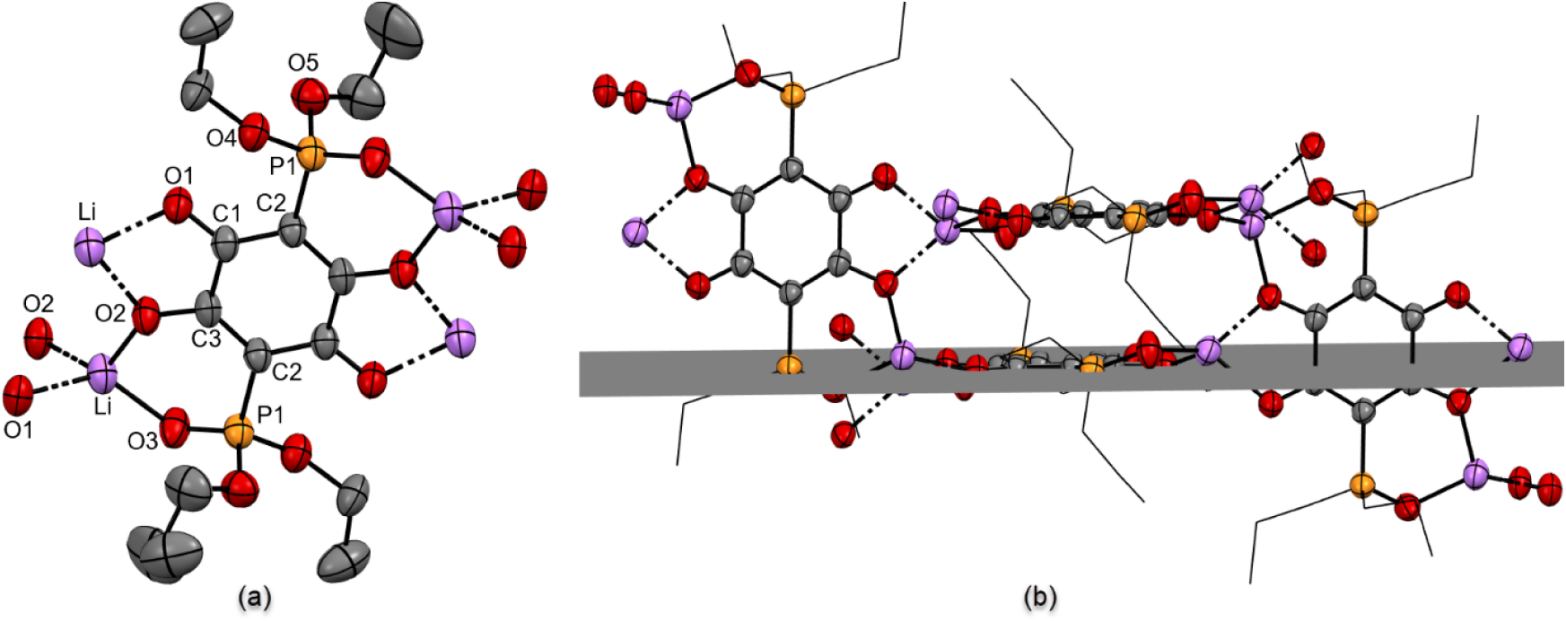
(a) Thermal ellipsoid plot (probability level 50%) of Li_2_ **3b**. The terminal methyl group in the OCH_2_CH_3_ is disordered over two positions. These were modeled with partial occupancy of terminal methyl groups [major conformer 0.64(4)] and involved 5 restraints on C–O and C–C bond distances. (b) Connectivity pattern of lithium ions and anilate dianions **3b**. For clarity, hydrogen atoms of the C–H bonds are omitted; ethyl groups are shown in wireframe.

**Figure 10:**
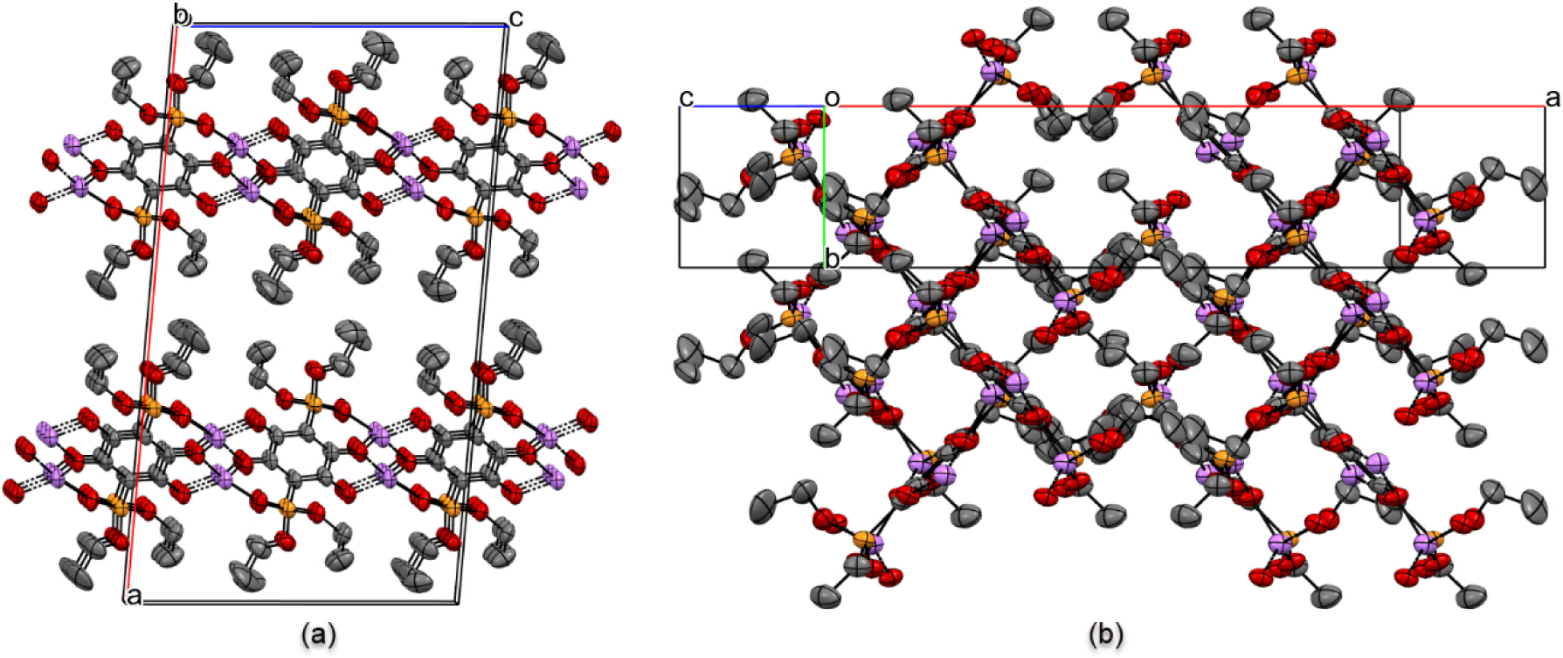
(a) Extended packing fragment of Li_2_ **3a** viewed along the *c* axis of the unit cell (layer structure); (b) same fragment viewed rotated 20°along the vertical (b) axis. Hydrogen atoms of the C–H bonds are omitted for clarity.

Structural parameters of anilate dianions **3a** and **3b** in their ammonium and lithium salts are compiled in Table 2. Values of C–O bond lengths determined for atoms within C_6_O_4_^2–^ units range from 1.2279(16) Å to 1.268(4) Å. Each anilate ring features four shorter C–C bonds ranging from 1.397(5) to 1.4272(13) Å and two substantially longer ones (1.535(5) to 1.5503(13) Å). These features are consistent with a resonance form where the anilate ring is considered as two W-shaped 5-atom O–C–C–C–O fragments are connected into a ring by long C–C bonds (Scheme 4, right).^40,41^

**Scheme 4:**
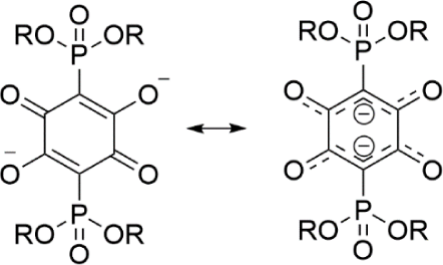
Resonance Forms of the Anilate Unit

### Cyclic Voltammetry

New anilic acids H_2_ **3a** and H_2_ **3b** and their ammonium and lithium salts were also investigated by cyclic voltammetry. Compounds H_2_ **3a** and H_2_ **3b** are sufficiently soluble in both water and polar organic solvents, thus allowing for CV measurements in both protic and aprotic media. However, CV scans conducted on aqueous solutions of dihydroxyquinones H_2_ **3a**–H_2_ **3b** exhibited poorly defined redox wave profiles, which we attribute to the effects of hydrogen bonding/proton transfer affecting electron transfer processes.^42,43^ Measurements conducted in acetonitrile solutions produced better-resolved potential/current responses, with the profiles of their CV scans shown in Figure 11. Both molecules undergo two irreversible reduction events in the cathodic scan, with peak current potentials at −0.69 V and −1.31 V for H_2_ **3a**, and −0.77 V and −1.45 V for H_2_ **3b** (vs Fc/Fc^+^). We attribute these processes to successive 1e^–^ reductions of the quinone moiety. Upon the return scan, both compounds display a broad irreversible reduction wave (0.12 and 0.15 V for H_2_ **3a** and H_2_ **3b**, respectively). This redox behavior is very similar to that reported earlier for 2,5-dihydroxyquinones appended with −P­(=O)­Ph_2_ or -P­(=O)*i*Pr_2_ substituents.^24^

**Figure 11:**
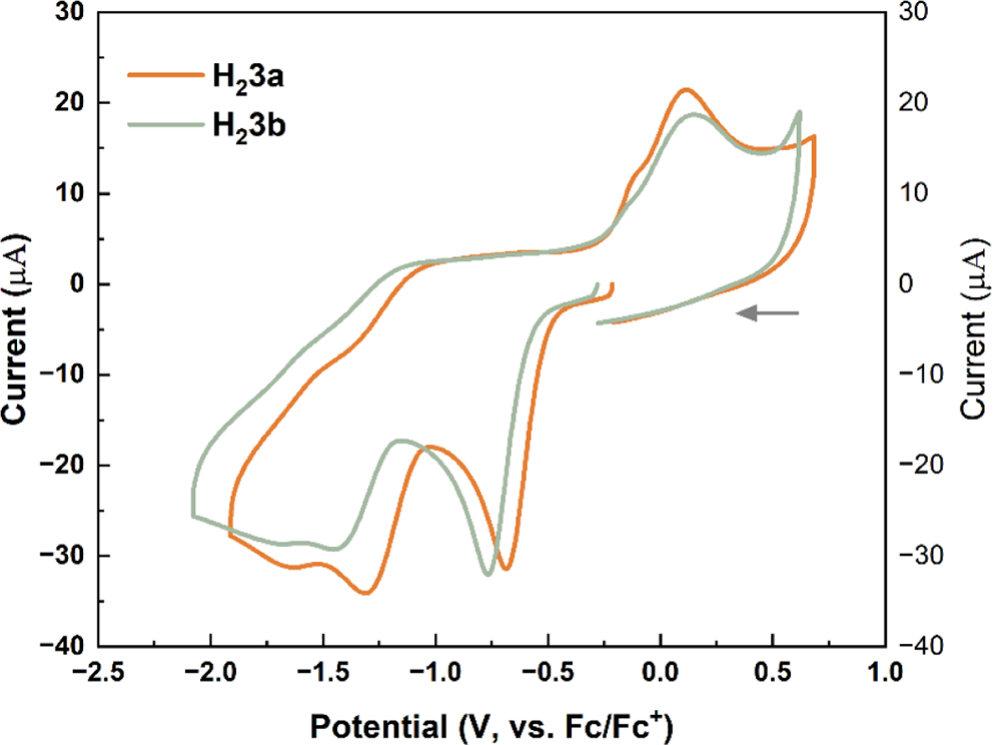
CV plots for H_2_ **3a** (1.9 mM) and H_2_ **3b** (1.8 mM) in CH_3_CN with 0.1 M NEt_4_BF_4_ as the supporting electrolyte; scan rate 200 mV/s.

Cyclic voltammetry profiles recorded for aqueous solutions of ammonium salts are shown in Figure 12. Both compounds undergo reduction processes in the cathodic region which are attributed to reduction of quinone moieties in **3a**–**b**, but the profiles of these scans are somewhat different. Reduction of **3a** is characterized by a single broad wave with a peak current potential at −0.72 V (vs Ag/AgCl). We tentatively assigned this process to the 2e^–^ reduction process **3a**→**3a** ^2–^. The CV recorded for (NH_4_)_2_ **3b** (under the same conditions) shows a reduction wave that consists of two overlapping processes with peak current potentials estimated at −0.45 and −0.65 V (Figure 12, yellow line). This implies that unlike **3a**→**3a** ^2–^ process discussed earlier, the reduction of **3b** in (NH_4_)_2_ **3b** proceeds in two 1e^–^ steps. Upon the return scan, both salts show one broad oxidation wave with essentially matching peak current potentials (0.15 V for (NH_4_)_2_ **3a** and 0.14 V for (NH_4_)_2_ **3b**). One possible reason for the different CV profiles of (NH_4_)_2_ **3a** and (NH_4_)_2_ **3b** in the cathodic region could be the differences in p*K*_a_ values of the parent acids H_2_ **3a** and H_2_ **3b**.^44^ At present, we have ongoing investigations where additional salts featuring dianions **3a**–**b** and other alkali metal cations are being scrutinized, and we anticipate addressing this issue in our future publications.

**Figure 12:**
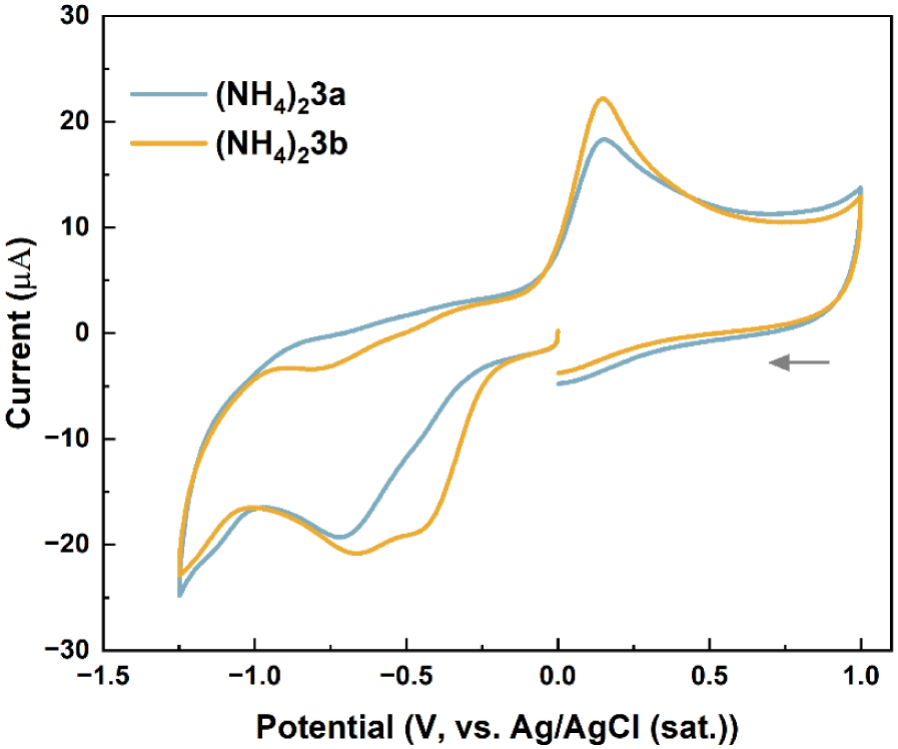
CV plots for (NH_4_)_2_ **3a** (1.9 mM) and (NH_4_)_2_ **3b** (1.8 mM) in water, with 0.1 M NEt_4_BF_4_ as the supporting electrolyte; scan rate 200 mV/s.

When aqueous solutions of salts Li_2_ **3a** and Li_2_ **3b** were subjected to cyclic voltammetry measurements with LiCl as the supporting electrolyte (0.10 M), the CV profiles obtained showed very broad, poorly discernible signals. Better-resolved responses were observed when these measurements were carried out with 0.10 M LiOH as the supporting electrolyte (Figure 13). Both compounds display one irreversible redox wave during the cathodic scan (peak current potentials of −1.36 V and −1.27 V for Li_2_ **3a** and Li_2_ **3b**, respectively). We attribute these processes to two-electron reductions of quinone moieties in **3a**–**b**, where proton involvement is limited due to the higher pH of the media.^45^ These were accompanied by broad irreversible waves during the anodic scans (−0.49 V and −0.59 V). For comparison, cyclic voltammetry of substituent-free 2,5-dihydroxy-1,4-benzoquinone recorded under similar conditions (aqueous media at pH 13) showed a quasireversible 2-electron redox event at −0.68 V (vs SHE).^16^

**Figure 13:**
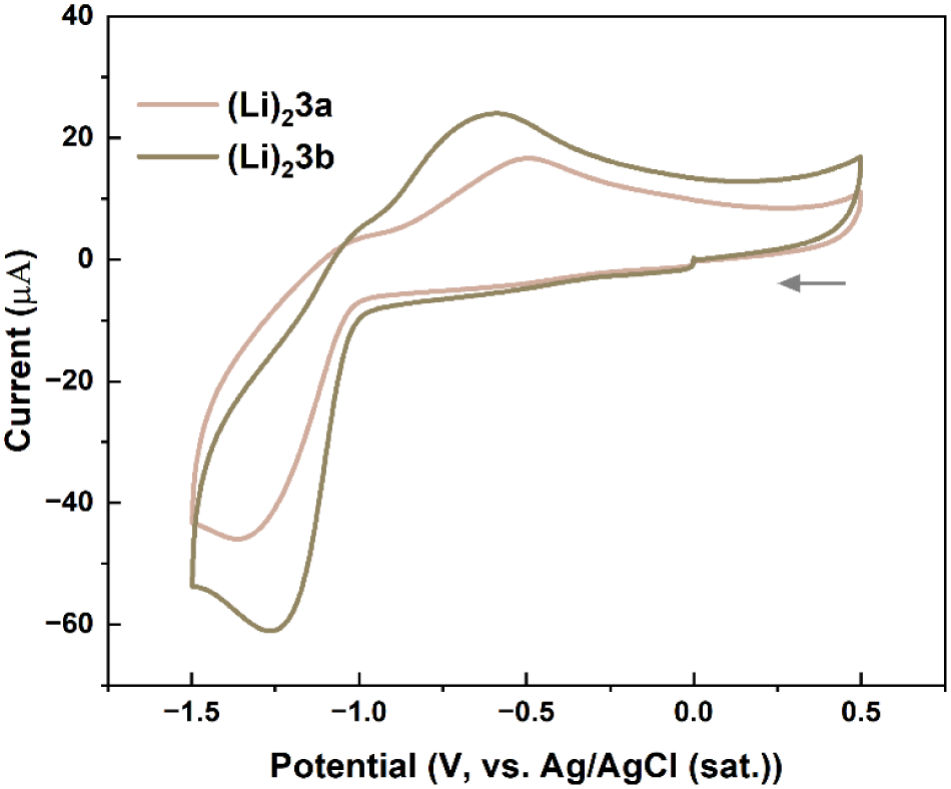
CV plots for Li_2_ **3a** (2.1 mM) and Li_2_ **3b** (3.0 mM) in water, with 0.1 M LiOH as the supporting electrolyte; scan rate 200 mV/s.

## Conclusions

Synthetic protocols enabling the synthesis of 2,5-dihydroxy-1,4-benzoquinones appended with −P­(=O)­(OR)_2_ (R = Me or Et) groups were established. The presence of phosphonato groups renders these new anilic acids soluble in both water and polar organic solvents. Single-crystal X-ray diffraction experiments performed on new ammonium and lithium anilates obtained in this work revealed that oxygen atoms from −P­(=O)­(OR)_2_ groups function as hydrogen bond acceptors and donor sites toward lithium cations. Extended open channels present in crystals of lithium salts Li_2_ **3a** and Li_2_ **3b** suggest that 2,5-dihydroxy-1,4-benzoquinones appended with phosphonato groups may be suitable for developing ion-conducting or charge storage materials, similar to those based on 1,4-hydroquinone appended with two phosphonato or phosphinato groups.^46,47^ Cyclic voltammetry experiments show that 2,5-dihydroxy-1,4-benzoquinone cores in both the new anilic acids and their salts undergo expected two-electron redox processes, which are strongly influenced by hydrogen bonding and/or the pH of the media.

## Experimental Methods

### General Information

1,4-Dichloro-2,5-dimethoxybenzene was prepared according to previously published procedures.^48,49^ Anhydrous THF and diethyl ether were obtained by distillation from deep blue-purple sodium-benzophenone solutions and were stored under N_2_ in airtight containers. Anhydrous methanol was obtained by distillation from Mg metal (with a small quantity of I_2_ added). Triethylamine and dichloromethane were purified by using the PureSolv system (Innovative Technology). Dimethylchlorophosphate and diethylchlorophosphate were purchased from TCI and degassed by “freeze-pump-thaw” method prior to use. Other solvents were purchased from commercial suppliers and used as received. Syntheses of 1,4-dichloro-2,5-dihydroxybenzene and compounds **1a**–**b** and **2a**–**b** were carried out under an air-free atmosphere, employing double manifold Schlenk line techniques.

^1^H NMR (400 MHz), ^13^C NMR (100 MHz), and ^31^P­{H} NMR (162 MHz) measurements were carried out on Varian MR-400 and/or Bruker Avance Neo-400 spectrometers. Spectra were referenced to SiMe_4_ (^1^H and ^13^C) by using residual solvent signals and 85% H_3_PO_4_ (^31^P) (external reference). FT-IR measurements were carried out on a Nicolet iS10 FT-IR spectrometer. High-resolution mass spectroscopic measurements were carried out at the Mass Spectrometry and Metabolomics Core, Michigan State University (East Lansing, MI 48824-1319), or at the Northern Illinois University Molecular Analysis Core (MAC) Laboratory, La Tourette Hall (LaT), Room 304, DeKalb, IL 60115. Elemental analyses were performed at Galbraith Laboratories Inc., (P.O. Box 51610, Knoxville, TN 37950-1610).

Cyclic voltammetry measurements were carried out on a WaveDriver 10 potentiostat (Pine Research). Dry acetonitrile was obtained by distillation from CaH_2_. Deionized water was obtained using a Milli-Q Direct 16 system. A standard 3-electrode cell was used, with the glassy carbon disk (0.071 cm^2^) and Pt wire used as working and counter electrodes, respectively. Measurements taken in CH_3_CN (H_2_ **3a** and H_2_ **3b**) used a Ag wire as the pseudoreference electrode and recrystallized ferrocene as the internal standard; the supporting electrolyte was dried at 110 °C for at least 2 h. Measurements taken in H_2_O were referenced to a Ag/AgCl (saturated) reference electrode (Pine Research). All measurements were carried out under a dinitrogen atmosphere.

### X-ray Data Collection and Refinement

X-ray diffraction data were collected at the University of Portland Diffraction Facility on an Oxford-Rigaku Gemini automated single-crystal diffractometer using CrysAlisPro for data collection, reduction, and absorption corrections.^50^ Compounds **2a**, H_2_ **3a**, H_2_ **3b**, (NH_4_)_2_ **3b** 2H_2_O, and Li_2_ **3b** were examined by single-crystal X-ray diffraction at room temperature; compounds (NH_4_)_2_ **3a**·H_2_O and Li_2_ **3a**·2H_2_O were studied at 110 K. Experimental parameters and general crystallographic data are listed in Table S1. Representative crystals of each compound were selected, snagged with fluorocarbon oil on a small nylon loop, and, for low-temperature determinations, quickly transferred to the cooled goniometer. Reflection data were collected with Mo Kα radiation (see Table S1) except for the lithium compounds and H_2_ **3b** for which Cu Kα radiation was used. Structures were solved using SHELXS-97 ^51^ and refined with SHELX-2018.^52^ Non-H atom positions were refined to convergence with associated anisotropic librational factors; H atoms were placed in calculated positions with isotropic librational factors equal to 120% of the equivalent isotropic librational factors for the adjacent atoms (150% for methyl and O–Hs); water H atoms were found in difference density maps and placed in these locations with a restraint on the O–H distance.

### Syntheses

#### 1,4-Dichloro-2,5-dihydroxybenzene

A Schlenk flask (250 mL) containing a solution of 1,4-dichloro-2,5-dimethoxybenzene (9.850 g, 47.6 mmol) in anhydrous dichloromethane (100 mL) was cooled to −60 °C. The reaction mixture was stirred, and BBr_3_ (9.1 mL, 95.2 mmol) was slowly added via syringe. The reaction mixture was allowed to attain room temperature and was stirred overnight. All volatiles were removed under reduced pressure, the remaining solid was cooled to −60 °C, and anhydrous methanol (100 mL) was then slowly added via syringe. The reaction mixture was allowed to attain room temperature and was stirred overnight. All volatiles were removed under reduced pressure, leaving behind the crude product as a gray solid. Additional purification was carried out by crystallization from hot acetonitrile. Yield: 7.890 g (92.6%). Spectroscopic characterizations matched the literature data.^53^

Compounds **1a**–**b** were prepared following the same methodology. Thus, a detailed procedure for only one of them, **1a**, is outlined.

#### 1,4-Dichloro-2,5-bis­(dimethylphosphato)­benzene **(1a)**

1,4-Dichloro-2,5-dihydroxybenzene (3.970 g, 22.2 mmol) was dissolved in 100 mL of anhydrous THF. In a separate flask, a solution of dimethylchlorophosphate (4.80 mL, 44.5 mmol) was prepared in 30 mL of anhydrous THF. The solution of dimethylchlorophosphate was transferred (via cannula) to the flask containing 1,4-dichloro-2,5-dihydroxybenzene, and the mixture was stirred and cooled to 0 °C. Anhydrous triethylamine (6.20 mL, 44.5 mmol) was slowly added (via syringe) to the reaction mixture. A white precipitate ([HNEt_3_]­Cl) formed immediately. The reaction flask was allowed to warm to room temperature, and the mixture was stirred overnight. The next day, the flask was disconnected from the Schlenk line, and further workup was carried out using standard benchtop techniques. Deionized water (50 mL) was added, and the organic layer was separated. The aqueous layer was extracted with diethyl ether (30 mL), and the organic phases were combined and washed with a saturated solution of sodium carbonate (20 mL). After this, the organic layer was separated, dried over anhydrous Na_2_CO_3_, and then filtered. All volatiles were removed under reduced pressure, yielding crude **1a** as a pale orange solid. Crystallization from CH_2_Cl_2_ afforded **1a** as a white crystalline solid. Yield: 5.31 g (61.0%). Mp = 105.6–107.6 °C. FT-IR (selected bands, cm^–1^): 3029 (w), 2963 (w), 1481(s), 1368 (m), 1284 (s), 1091 (m), 1043 (s), 992 (m), 936 (s). ^1^H NMR (CDCl_3_): δ 7.53 (s, 2H), 3.91 (d, ^3^J_PH_ = 11.5 Hz, 12 H). ^13^C NMR (CDCl_3_): δ 143.7 (dd, ^2^J_PC_ = 6.3 Hz, ^5^J_PC_ = 1.6 Hz, C_Ar_–O), 124.5 (dd, ^3^J_PC_ = 7.5 Hz, ^4^J_PC_ = 1.8 Hz, C_Ar_–Cl), 122.7 (m, C_Ar_–H), 55.4 (d, ^2^J_PC_ = 6.6 Hz, O–CH_3_). ^31^P NMR (CDCl_3_): δ 4.8 (s). HRMS (ES+): calcd for C_10_H_15_Cl_2_O_8_P_2_ [MH]^+^ *m*/*z* = 394.9619; found 394.9622.

#### 1,4-Dichloro-2,5-bis­(diethylphosphato)­benzene **(1b)**

The reaction was conducted on the same scale as that described for **1a**. Compound **1b** was isolated as a viscous orange oil. Yield: 7.532 g (75.8%). FT-IR (selected bands, cm^–1^): 2982 (m), 1480 (s), 1395 (m), 1369 (m), 1248 (s), 1192 (s), 1164 (m), 1024 (s), 984 (s). ^1^H NMR (CDCl_3_): δ 7.56 (m, 2 H), 4.29–4.23 (m, 8 H), 1.38 (t, ^3^J_HH_ = 7.2 Hz, 12 H). ^13^C NMR (CDCl_3_): δ 143.8–143.7 (m, C_Ar_–O), 124.3 (dd, ^3^J_PC_ = 7.8 Hz, ^4^J_PC_ = 1.6 Hz, C_Ar_–Cl), 122.6 (s, C_Ar_–H), 65.3 (d, ^2^J_PC_ = 6.6 Hz, O–CH_2_CH_3_), 16.1 (d, ^3^J_PC_ = 6.8 Hz, O–CH_2_ CH_3_). ^31^P NMR (CDCl_3_): δ −7.0 (s). HRMS (ES-): calcd for C_14_H_22_Cl_2_O_8_P_2_ (M^–^) *m*/*z* = 450.0167; found 420.9777 [M–C_2_H_5_]^−^.

Compounds **2a**–**b** were prepared by following the same methodology. Thus, a detailed procedure for only one of them, **2a**, is outlined.

#### 2,5-Dichloro-3,6-bis­(dimethylphosphonato)-1,4-hydroquinone **(2a)**

A Schlenk flask containing a solution of **1a** (7.610 g, 19.3 mmol) in THF (250 mL) was placed in a shallow Dewar and cooled to −80 °C using an isopropanol/liquid N_2_ bath. A solution of freshly prepared LDA (2.1 mol equiv, in Et_2_O (150 mL)) was added in portions over an ∼30 min period. A bright yellow precipitate gradually formed, and the reaction mixture was stirred overnight while it slowly returned to room temperature. The reaction flask was then disconnected from the Schlenk line and cooled to 0 °C in an ice bath for 15 min and concentrated HCl (6.5 mL, ∼5 mol equiv) was added slowly. The solid gradually dissolved, and a clear yellow solution was formed. The contents of the flask were transferred into a separatory funnel, a small quantity of the aqueous layer was separated, and the organic layer was dried over anhydrous Na_2_SO_4_. The liquid was separated from the solid by filtration, and slow evaporation of volatiles yielded **2a** as a colorless crystalline substance. Yield: 6.621 g (85.5%). Mp = 197.4–197.7 °C. FT-IR (selected bands, cm^–1^): 2981 (m, br), 2883 (w), 1453 (w), 1397 (s), 1206 (m), 1170 (s), 1024 (s), 880 (s), 848 (m), 799 (s). ^1^H NMR (CDCl_3_): δ 11.54 (br, OH), 3.84 (d, ^3^J_PH_ = 12.0 Hz, −OCH_3_). ^13^C NMR (CDCl_3_): δ 151.7 (dd, ^2^J_PC_ = 15.3 Hz, ^3^J_PC_ = 6.0 Hz, C_Ar_–OH), 123.0 (d, ^2^J_PC_ = 19.3 Hz, C_Ar_–Cl), 113.9 (dd, ^1^J_PC_ = 177.9 Hz, ^4^J_PC_ = 2.6 Hz), 53.8 (d, ^2^J_PC_ = 5.5 Hz, −OCH_3_). ^31^P NMR­(CDCl_3_): δ 20.5 (s). HRMS (ES-): calcd for C_10_H_13_Cl_2_O_8_P_2_ [M–H]^−^ *m*/*z* = 392.9463; found 392.9474.

#### 2,5-Dichloro-3,6-bis­(diethylphosphonato)-1,4-hydroquinone (**2b**)

The reaction was conducted starting with 16.7 mmol of **1b**. Compound **2b** was obtained as a white crystalline substance by crystallization from acetone at −22 °C. Yield: 6.057 g (79.6%). Mp = 148.9–150.6 °C. FT-IR (selected bands, cm^–1^): 2981 (m, (br), 2814 (w), 2756 (w, br), 1476 (m), 1447 (w), 1403 (s), 1314 (w), 1292 (w), 1208 (m), 1192 (m), 1173 (s), 1001 (s), 966 (s), 879 (s), 815 (m), 785 (s). ^1^H NMR (CDCl_3_): δ 11.68 (s, 2 H, −OH), 4.28–4.10 (m, 8 H, OCH_2_CH_3_), 1.37 (t, ^3^J_HH_ = 7.0, 12 H, OCH_2_CH_3_). ^13^C NMR (CDCl_3_): δ 151.4 (dd, ^2^J_PC_ = 14.9 Hz, ^3^J_PC_ = 6.1 Hz, C_Ar_–OH), 123.0 (d, ^2^J_PC_ = 19.0 Hz, C_Ar_–Cl), 114.9 (d, ^1^J_PC_ = 176.3 Hz, ^4^J_PC_ = 2.5 Hz, C_Ar_-P), 64.0 (d, ^2^J_PC_ = 5.5 Hz, −OCH_2_CH_3_), 16.1 (d, ^3^J_PC_ = 6.9 Hz, −OCH_2_ CH_3_). ^31^P NMR (CDCl_3_): 17.5 (s). HRMS (ES−): calcd for C_14_H_21_Cl_2_O_8_P_2_ [M–H]^−^, *m*/*z* = 449.0089; found: 449.0096.

Compounds H_2_ **3a** and H_2_ **3b** were prepared by following the same methodology. Thus, a detailed procedure for only one of them, H_2_ **3a**, is outlined.

#### 2,5-Bis­(dimethylphosphonato)-3,6-dihydroxy-1,4-benzoquinone (H_2_ **3a** *)*

Solid **2a** (3.961 g, 10.0 mmol) was placed in a round-bottom flask containing deionized water (150 mL). The mixture was magnetically stirred, and a solution of KOH (5.611 g, 100.0 mmol) in water (10 mL) was added. Continued stirring for ∼15 min led to the formation of a clear orange-brown solution. A solution of K_2_S_2_O_8_ (2.842 g, 10.5 mmol) in water (10 mL) was added, which led to a change of color to deep yellow. The reaction mixture was stirred for 1 h, and the contents were poured into a large evaporation dish. Slow evaporation (2–3 days) to dryness led to the deposition of a mixture of yellow and colorless crystalline solids. The solids were scraped off and placed in a beaker, followed by the addition of CH_3_CN (40 mL) and concentrated HCl (10 mL). The mixture was stirred for ∼10 min, and the deep yellow solution was separated from the white crystalline solid by filtration. The solid was rinsed with an additional quantity (∼5 mL) of CH_3_CN. The solid was discarded, and all the volatiles were removed under reduced pressure, leaving behind H_2_ **3a** as a pasty yellow solid. It was further purified by crystallization from acetone, yielding H_2_ **3a** as a brown-yellow crystalline solid. Yield: 1.490 g (41.6%). Mp = >170 °C (decomp). FT-IR (selected bands, cm^–1^): 2986 (w, br), 1668 (s), 1572 (s), 1434 (s), 1348 (m), 1266 (s), 1184 (m), 1144 (m), 1026 (s), 984 (s), 909 (m), 839 (s), 759 (m). ^1^H NMR (CDCl_3_): δ 11.3–10.2 (br), 2H, −OH), 3.88 (d, 12H, ^3^J_PH_ = 12.0 Hz, −OCH_3_). ^1^H NMR (D_2_O): δ 3.70 (d, ^3^J_PH_ = 11.6 Hz, 12H, −OCH_3_); signal for −OH protons was not observed. ^13^C NMR (CDCl_3_): δ 102.7 (d, ^1^J_CP_ = 175 Hz, P–C), 54.8 (d, ^2^J_CP_ = 6.5 Hz, −OCH_3_); C=O and C–OH signals from the 2,5-dihydroxyquinone ring were not observed. ^13^C NMR (D_2_O): δ 176.1 (m, br), C=O/C_ring_–O), 97.7 (d, ^1^J_PC_ = 183 Hz, P–C), 53.1 (d, ^2^J_PC_ = 5.4 Hz, −OCH_3_).^31^P NMR (CDCl_3_): δ 20.7 (s). ^31^P NMR (D_2_O): δ 22.6 (s, br). HRMS (ES+): calcd for C_10_H_15_O_10_P_2_ [MH]^+^ *m*/*z* = 357.0140, found 357.0138.

#### 2,5-Bis­(diethylphosphonato)-3,6-dihydroxy-1,4-benzoquinone (H_2_ **3b** *)*

The reaction was conducted starting out with 2 mmol (0.9023 g) of **2b**; the final product was crystallized from THF. Yield: 0.645 g (78.5%). Mp = 136.3–138.0 °C. FT-IR (selected bands, cm^–1^): 2992 (w), 1668 (s), 1576 (s), 1470 (w), 1441 (m), 1347 (m), 1257 (s), 1155 (s), 1013 (s), 980 (s), 961 (s), 913 (m), 847 9m), 743 (m). ^1^H NMR (CDCl_3_): δ ∼12.1–10.6 (br, 2H, OH), 4.30–4.15 (m, 8H, OCH_2_CH_3_), 1.35 (t, 12H. ^3^J_HH_ = 7.2 Hz, OCH_2_CH_3_). ^1^H NMR (D_2_O): δ 4.07–4.00 (m, 8H, OCH_2_CH_3_), 1.22 (t, 12 H, ^3^J_HH_ = 7.0 Hz, OCH_2_CH_3_). ^13^C NMR (CDCl_3_): δ 177.5 (br, C=O), 167.5 (br, C–OH), 103.5 (d, ^1^J_PC_ = 174.0 Hz, C–P), 64.8 (d, ^2^J_PC_ = 6.6 Hz, −OCH_2_CH_3_), 16.1 (d, ^3^J_PC_ = 7.0 Hz, -OCH_2_ CH_3_). ^13^C NMR (D_2_O): δ 175.9–175.8 (m, br), C=O/C_ring_–O), 99.9–98.0 (dm, P–C), 63.6 (d, ^2^J_PC_ = 5.1 Hz, −OCH_2_CH_3_), 15.6 (d, ^3^J_PC_ = 6.3 Hz, −OCH_2_ CH_3_). ^31^P NMR (CDCl_3_): δ 17.7 (s). ^31^P NMR (D_2_O): δ 18.9 (s, br). HRMS (ES+): calcd for C_14_H_23_O_10_P_2_ [MH]^+^, *m*/*z* = 413.0770, found 413.0770.

Ammonium salts (NH_4_)_2_ **3a** and (NH_4_)_2_ **3b** were prepared by the same methodology; therefore, a detailed procedure for (NH_4_)_2_ **3a** is provided

#### Ammonium 2,5-bis­(dimethylphosphonato)-3,6-dioxy-1,4-benzoquinonate, ((NH_4_)_2_ **3a**)

Dihydroxyquinone H_2_ **3a** (0.358 g, 1.00 mmol) was dissolved in deionized water (4 mL), followed by the addition of 167 μL of aqueous NH_3_ solution (15 M) (ca. 2.5 mmol of NH_3_). The mixture was stirred until a clear, deep yellow solution was formed. Stirring was then stopped, and the reaction mixture was left in the fume hood for slow evaporation of the solvent. Yellow rod-shaped crystals of (NH_4_)_2_ **3a** H_2_O formed after 4–5 days. These crystals were isolated by removing the residual solution and were air-dried. Yield: 0.310 g (76.0%). Mp = 182 °C (decomp). FT-IR (selected bands, cm^–1^): 3083 (m, br), 1606 (m), 1567 (s), 1465 (m), 1430 (m), 1381 (m), 1316 (s), 1235 (m), 1191 (m), 1151 (m), 1035 (s), 1007 (s), 838 (s), 818 (m), 791 (m), 766 (m). ^1^H NMR (D_2_O): δ 3.70 (d, ^3^J_PH_ = 11.7 Hz, −OCH_3_). ^13^C NMR (D_2_O): δ 176.7–176.6 (m, C–O/C=O (quinone ring)), 98.5–96.6 (dm, C–P), 53.1 (d, ^2^J_CP_ = 5.3 Hz). ^31^P NMR (D_2_O): δ 23.3 (s). Calcd for C_10_H_22_N_2_O_11_P_2_ ((NH_4_)_2_ **3a·**H_2_O): C: 29.42, H: 5.43, N: 6.86; found: C: 29.44, H: 5.05, N: 6.83.

#### Ammonium 2,5-Bis­(diethylphosphonato)-3,6-dioxy-1,4-benzoquinonate ((NH_4_)_2_ **3b**)

Synthesis of (NH_4_)_2_ **3b** was conducted with 2.00 mmol (0.822 g) of H_2_ **3b** in 9 mL of water and 333 μL of aqueous NH_3_ solution (15 M) (ca. 5.0 mmol of NH_3_). Bright yellow crystalline blocks of (NH_4_)_2_ **3b**·2H_2_O were isolated by filtration and were air-dried. Yield: 0.921 g (96.7%). Mp = 190 °C (decomp). FT-IR (selected bands, cm^–1^): 3369 (m, br), 3169 (m, br), 2981 (w, br), 1636 (w), 1538 (s), 1452 (s), 1393 (m), 1356 (m), 1297 (m), 1193 (s), 1019 (s), 960 (s), 874 (w), 796 (s), 764 (m). ^1^H NMR (D_2_O): δ 4.03–3.96 (m, 8H, −OCH_2_CH_3_), 1.25 (t, ^3^J_HH_ = 7.0 Hz, 12H, −OCH_2_CH_3_). ^13^C NMR (D_2_O): δ 181.1–180.9 (m, br), C–O/C=O (quinone ring)), 96.7 (d, ^1^J_CP_ = 180.9 Hz, C–P), 62.5 (d, ^2^J_CP_ = 5.1 Hz, −OCH_2_CH_3_), 15.6 (d, ^3^J_CP_ = 6.9 Hz, −OCH_2_ CH_3_). ^31^P­{H} NMR (D_2_O): δ 25.4 (s). Calcd for C_14_H_32_N_2_O_12_P_2_ ((NH_4_)_2_ **3b**·2H_2_O): C: 34.86, H: 6.69, N: 5.81; found: C: 34.74, H: 6.40, N: 5.85.

Lithium salts Li_2_ **3a** and Li_2_ **3b** were prepared by the same methodology; therefore, the detailed procedure for only Li_2_ **3a** is provided

#### Lithium 2,5-Bis­(dimethylphosphonato)-3,6-dioxy-1,4-benzoquinonate, (Li_2_ **3a** *)*

Solid LiOH (0.050 g, 2.1 mmol) was added to a stirred solution of H_2_ **3a** (0.356 g, 1.0 mmol) in deionized water (13 mL). A bright yellow solution formed immediately. After stirring for 30 min, the reaction mixture was removed from the stirring plate and left open for slow water evaporation. Bright yellow crystals started forming after ∼48 h. After 8 days, the bottom of the reaction vessel (20 mL vial) was covered with a layer of yellow crystalline solid and a small quantity (∼0.5 mL) of dark yellow solution. The liquid was removed using a pipet, and the remaining solid Li_2_ **3a**·2H_2_O was allowed to air-dry for another 2 days. Yield: 0.358 g (88.6%). FT-IR (selected bands, cm^–1^): 3563 (w, br), 3199 (w, br), 2952 (w), 2851 (w), 1550 (s), 1460 (m), 1441 (m), 1377 (m), 1309 (s), 1228 (m), 1210 (m), 1150 (w), 1042 (s), 996 (m), 871 (m), 818 (s), 753 (s). ^1^H NMR (D_2_O): δ 3.64 (d, ^3^J_PH_ = 11.6 Hz, −OCH_3_). ^13^C NMR (D_2_O): δ 181.1–180.9 (m, br), C–O/C=O (quinone ring)), 96.6–94.7 (dm, CP), 52.4 (d, ^2^J_CP_ = 5.3 Hz, −OCH_3_). ^31^P NMR (D_2_O): δ 28.8 (s). Calcd for C_10_H_16_Li_2_O_12_P_2_ (Li_2_ **3a**·2H_2_O): C: 29.73, H: 3.99; found: C: 29.52, H: 3.72.

#### Lithium 2,5-bis­(diethylphosphonato)-3,6-dioxy-1,4-benzoquinonate (Li_2_ **3b** *)*

The reaction was conducted in an analogous fashion, using 0.824 g (2.0 mmol) of H_2_ **3b** and 0.097 g (4.0 mmol) of LiOH, which were reacted in 18 mL of deionized water. Salt Li_2_ **3b** was isolated as a bright yellow crystalline solid. Yield: 0.741g (87.4%). FT-IR (selected bands, cm^–1^): 2980 (w), 2928 (w), 1625 (w), 1520 (s), 1475 (m, sh), 1445 (w, sh), 1385 (w), 1362 (w), 1318 (s), 1206 (s), 1165 (m), 1096 (w), 1067 (s), 1027 (s), 1027 (w), 961 (s), 941 (s), 863 (m), 812 (m), 787 (m), 740 (s). ^1^H NMR (D_2_O): δ 4.04–3.96 (m, 2H, −OCH_2_CH_3_), 1.25 (t, 3H, ^3^J_HH_ = 7.0 Hz, −OCH_2_CH_3_). ^13^C NMR (D_2_O): δ 181.1–180.9 (m, br), C–O/C=O (quinone ring)), 97.6–95.7 (m, P–C), 62.5 (d, ^2^J_CP_ = 4.9 Hz, OCH_2_CH_3_), 15.6 (d, ^3^J_CP_ = 6.6 Hz, OCH_2_ CH_3_). ^31^P NMR (D_2_O): δ 25.4 (s). Calcd for C_14_H_20_Li_2_O_10_P_2_ (Li_2_ **3b**): C: 39.65, H: 4.75; found: C: 39.19, H: 4.35 (repeated elemental analyses carried out on two separate samples were consistently slightly lower on carbon).

## Supplementary Material



## Notes

NMR spectra of **1a**–**b**, **2a**–**b**, H_2_ **3a**, H_2_ **3b**, (NH_4_)_2_ **3a**, (NH_4_)_2_ **3b**, Li_2_ **3a**, and Li_2_ **3b** are provided; supplementary crystallographic data were deposited at the Cambridge Crystallographic Data Centre; CCDC numbers are listed in Table 2; copies of the available material can be obtained free of charge by application to the director, CCDC, 12 Union Road, Cambridge CH2 1EZ, UK (fax: + 44 1223 336033; E-mail: deposit@ccdc.cam.ac.uk or http://www.ccdc.cam.ac.uk) (PDF)
