# Supplementary material for: 2,5-Dihydroxy-1,4-benzoquinones Appended with −P(=O)(OR)2 (R = Me or Et) Groups and Their Ammonium and Lithium Salts: Structural, Spectroscopic, and Electrochemical Properties
Source: ACS Omega. 2025 Oct 31;10(44):52954–67. doi: 10.1021/acsomega.5c07399 (PMC12612944; doi:10.1021/acsomega.5c07399)
Supplement: Supplementary file 1 [file ao5c07399_si_002.pdf]

2,5-Dihydroxy-1,4-benzoquinones appended with -P(=O)(OR)$_2$ (R = Me or Et) groups, and their ammonium and lithium salts: structural, spectroscopic, and electrochemical properties

Claire A. Kearney, Kailin M. Mooney, Jordan N. Sanders, Kai J. Edison, Milan H. Hague, S. Joseph Lippert, Timothy J. Dobson, Edward J. Valente, Eugenijus Urnezius[1]

Department of Chemistry & Biochemistry, University of Portland, 5000 N. Willamette Blvd., Portland, OR 97203, USA

Supplementary Material

[1] Corresponding author (urnezius@up.edu)

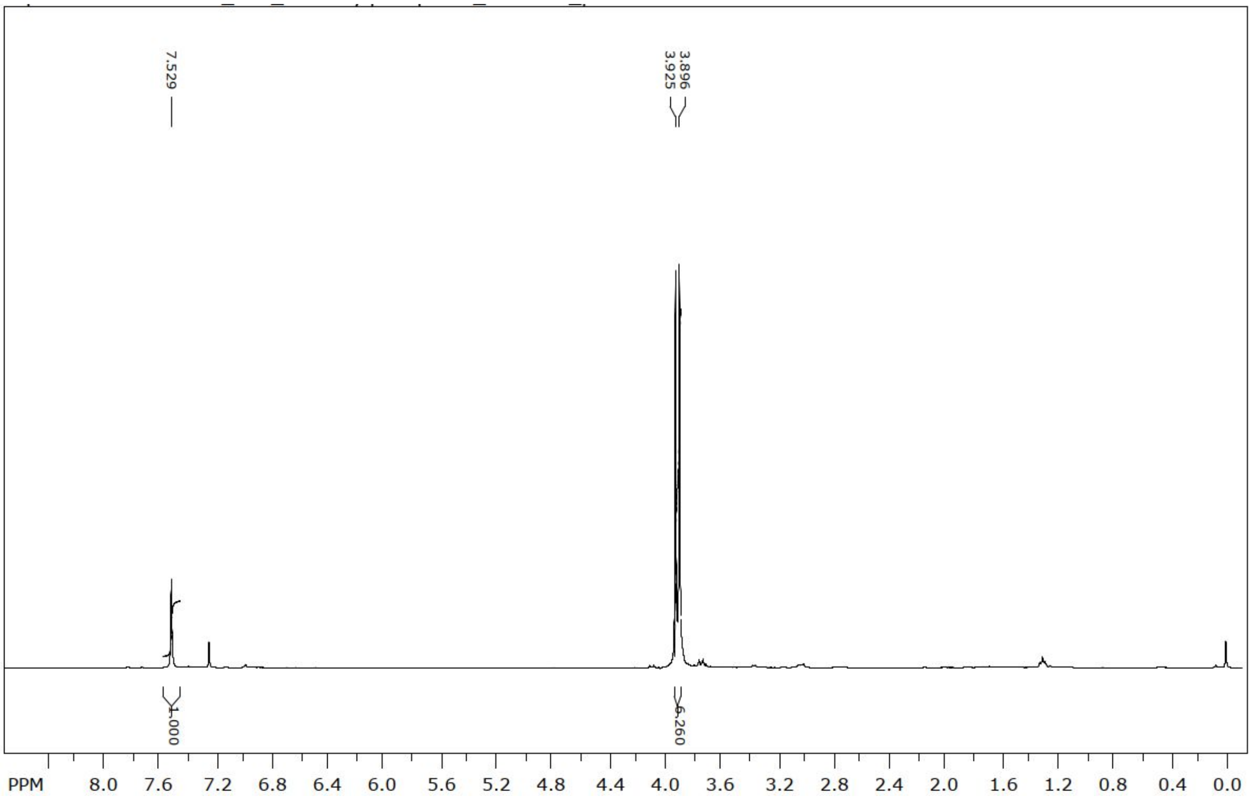

Figure S1. ^{1}H NMR spectrum of **1a** ($CDCl_3$).

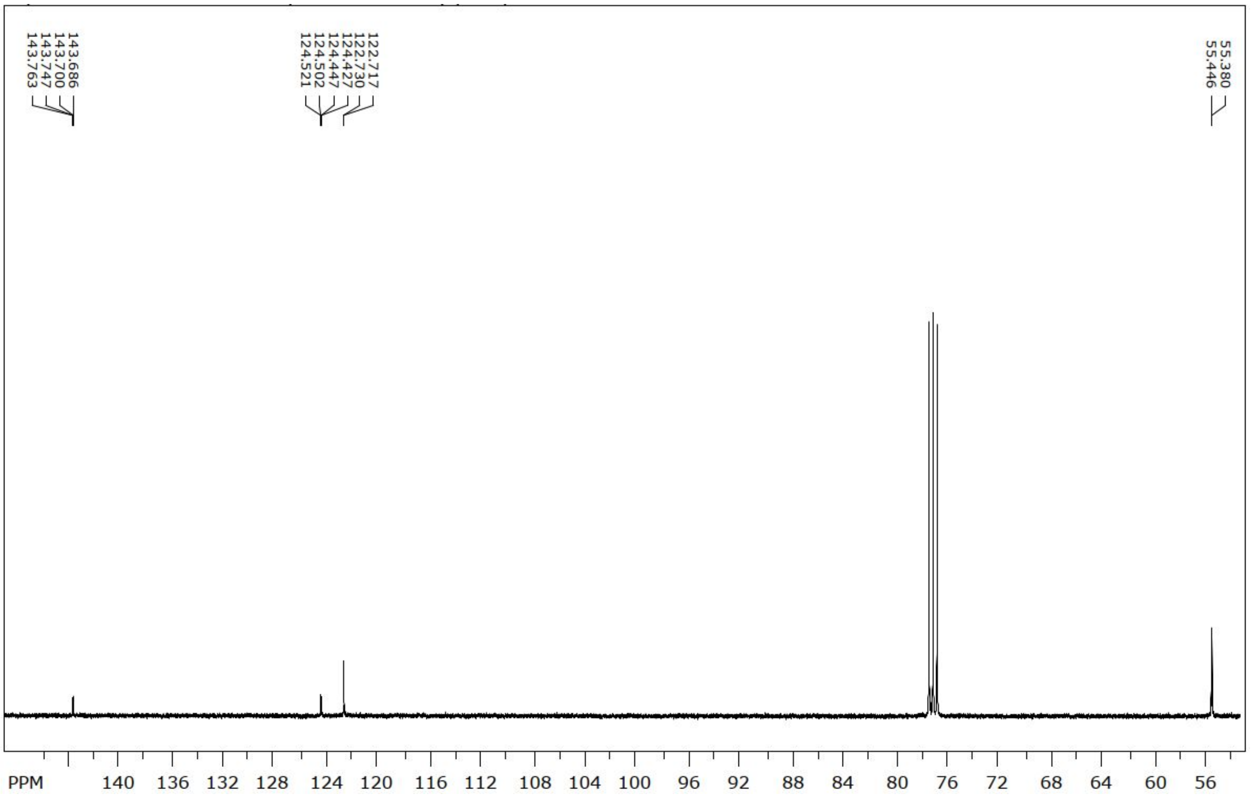

Figure S2. ^{13}C NMR spectrum of **1a** ($CDCl_3$).

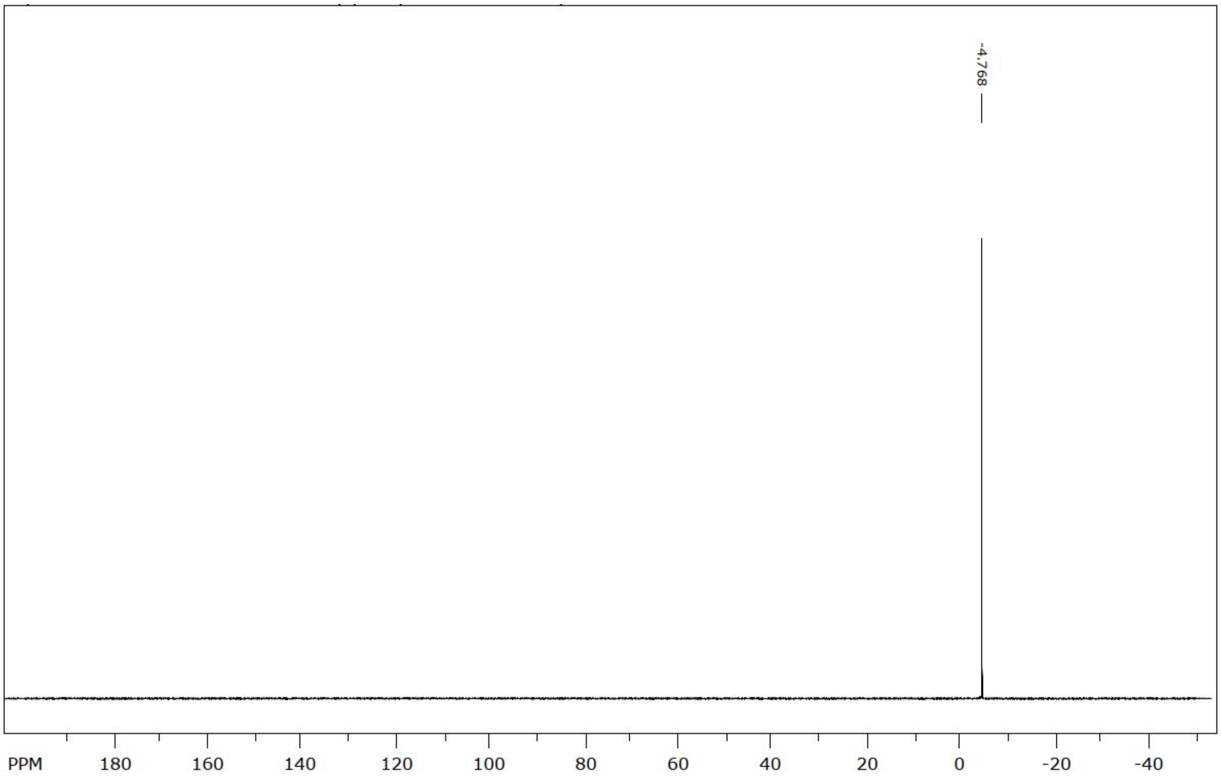

Figure S3. $^{31}P\{H\}$ NMR spectrum of **1a** ($CDCl_3$).

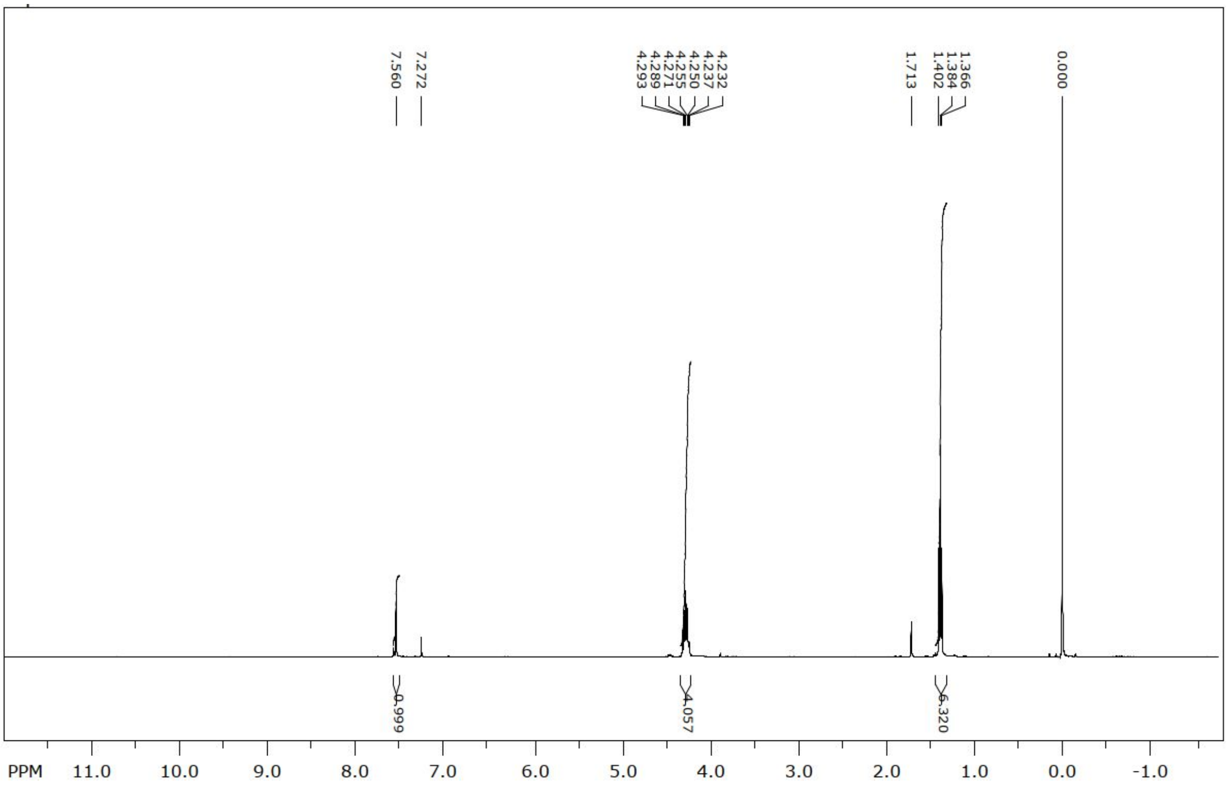

Figure S4. ^{1}H NMR spectrum of **1b** ($CDCl_3$).

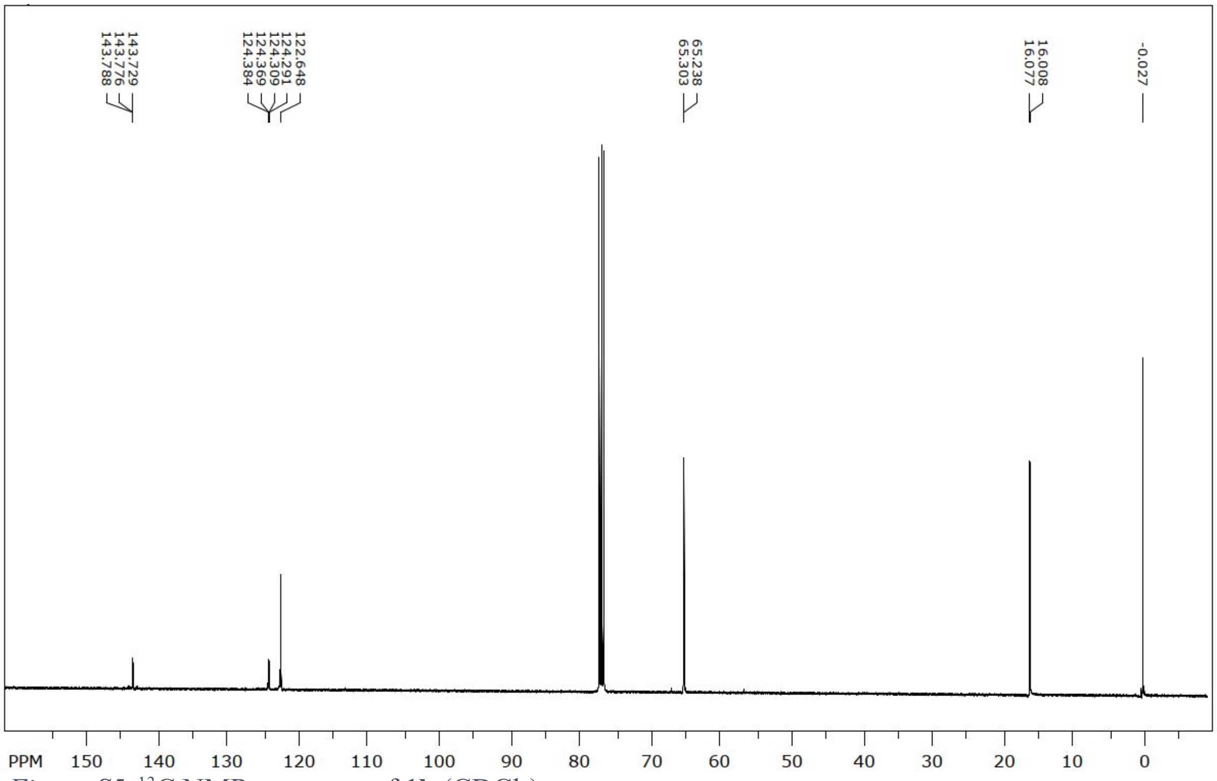

Figure S5. ^{13}C NMR spectrum of **1b** ($CDCl_3$)

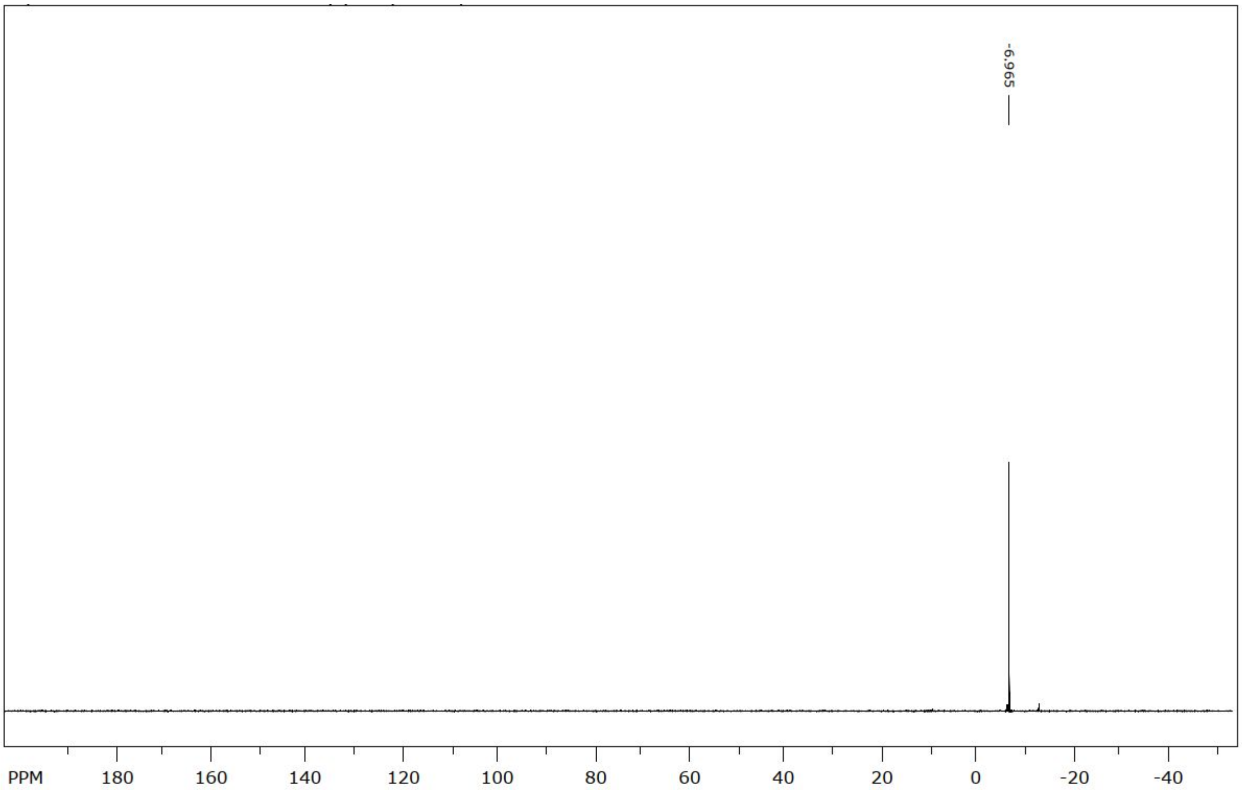

Figure S6. $^{31}P\{H\}$ NMR spectrum of **1b** ($CDCl_3$)

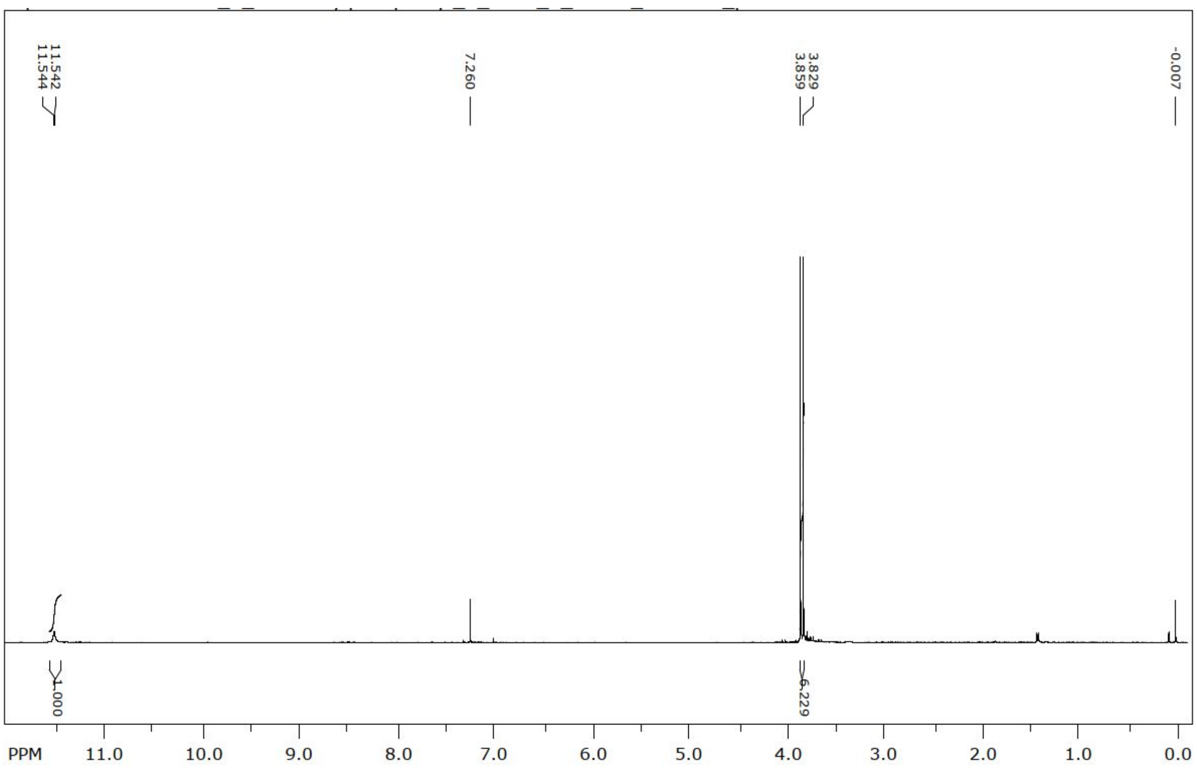

Figure S7. ^{1}H NMR spectrum of **2a** ($CDCl_3$)

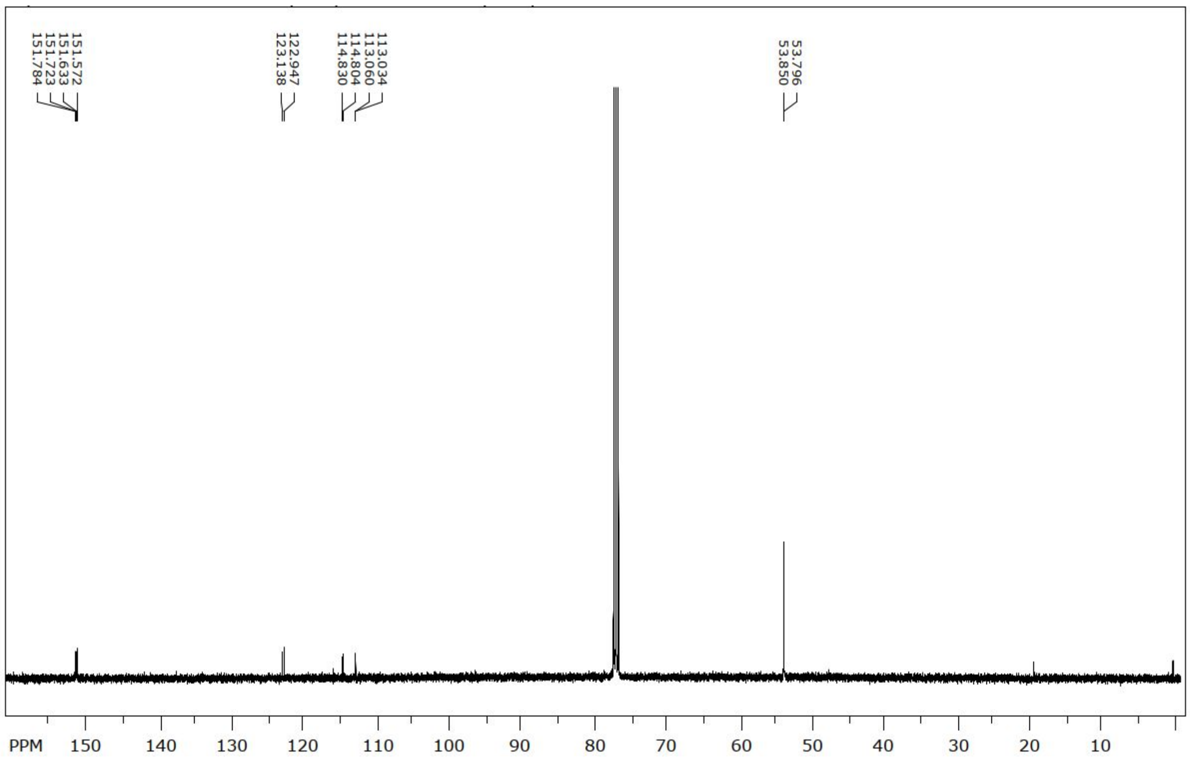

Figure S8. ^{13}C NMR spectrum of **2a** ($CDCl_3$)

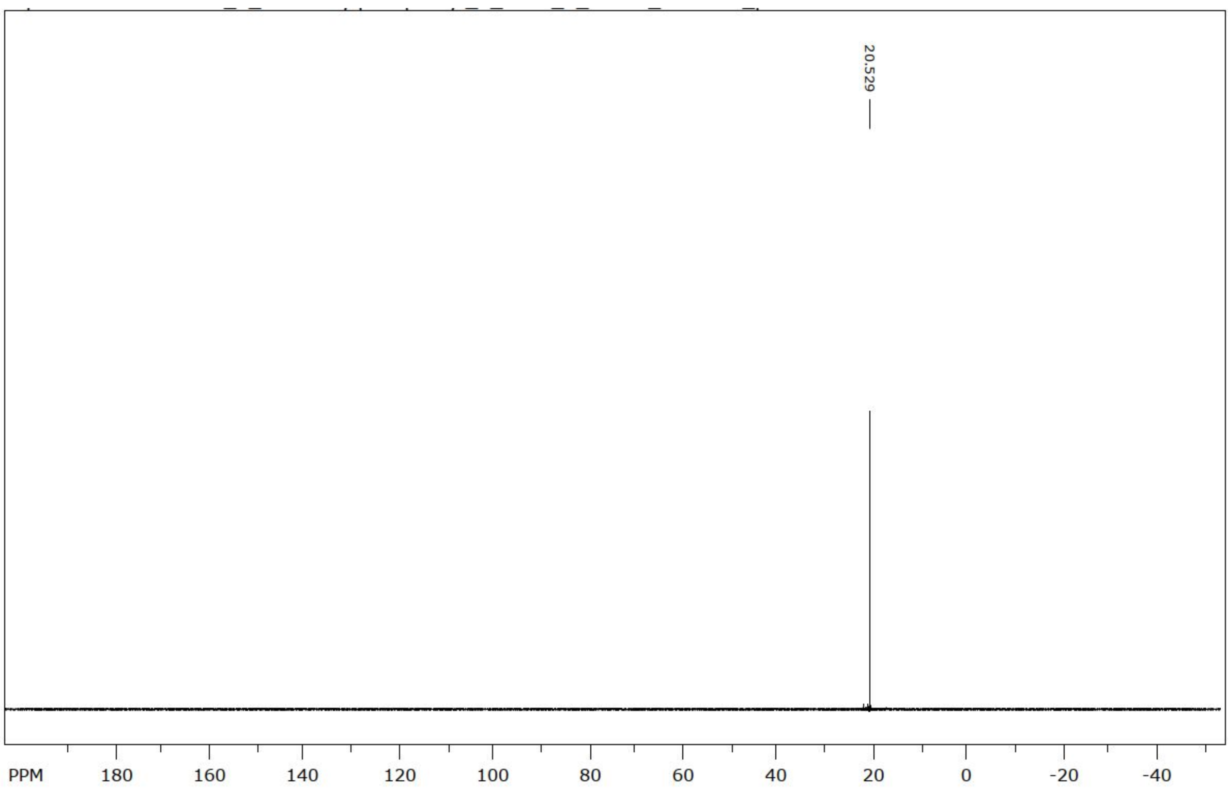

Figure S9. ^{31}P{H} NMR spectrum of **2a** ($CDCl_3$)

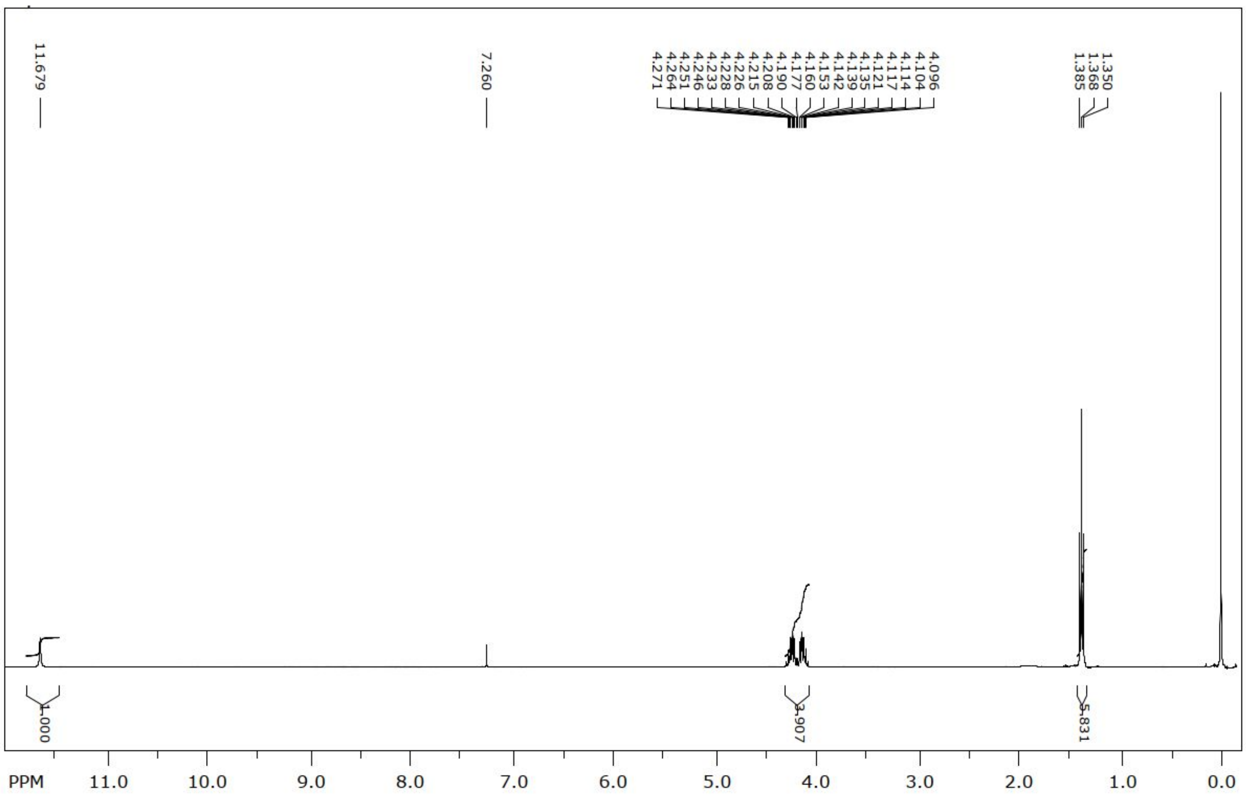

Figure S10. ^{1}H NMR spectrum of **2b** ($CDCl_3$)

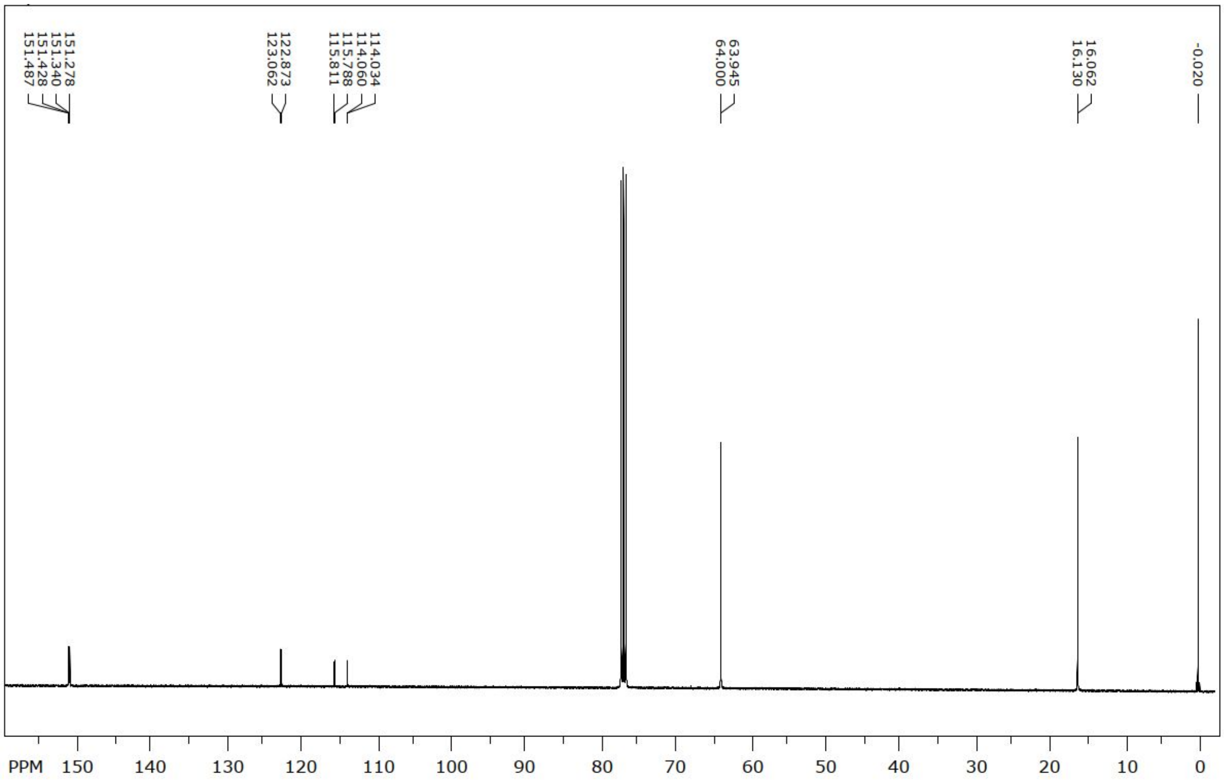

Figure S11. ^{13}C NMR spectrum of **2b** ($CDCl_3$)

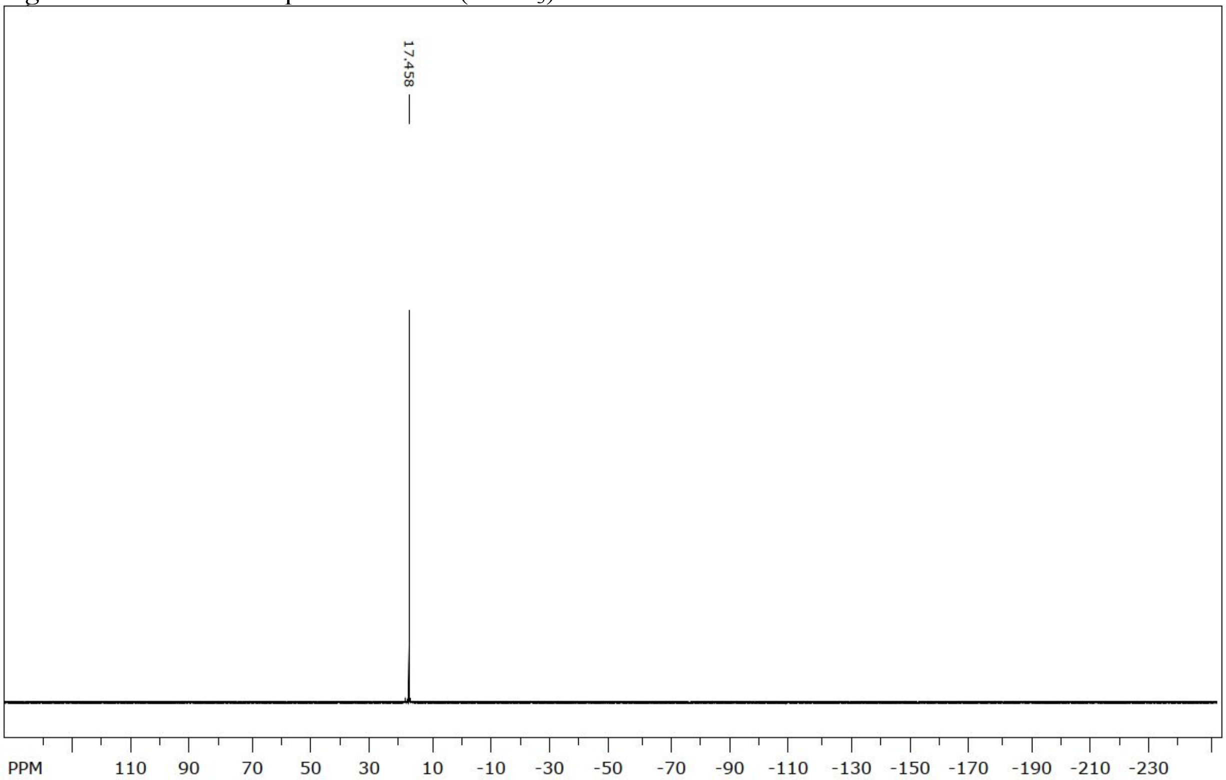

Figure S12. $^{31}P\{H\}$ NMR spectrum of **2b** ($CDCl_3$)

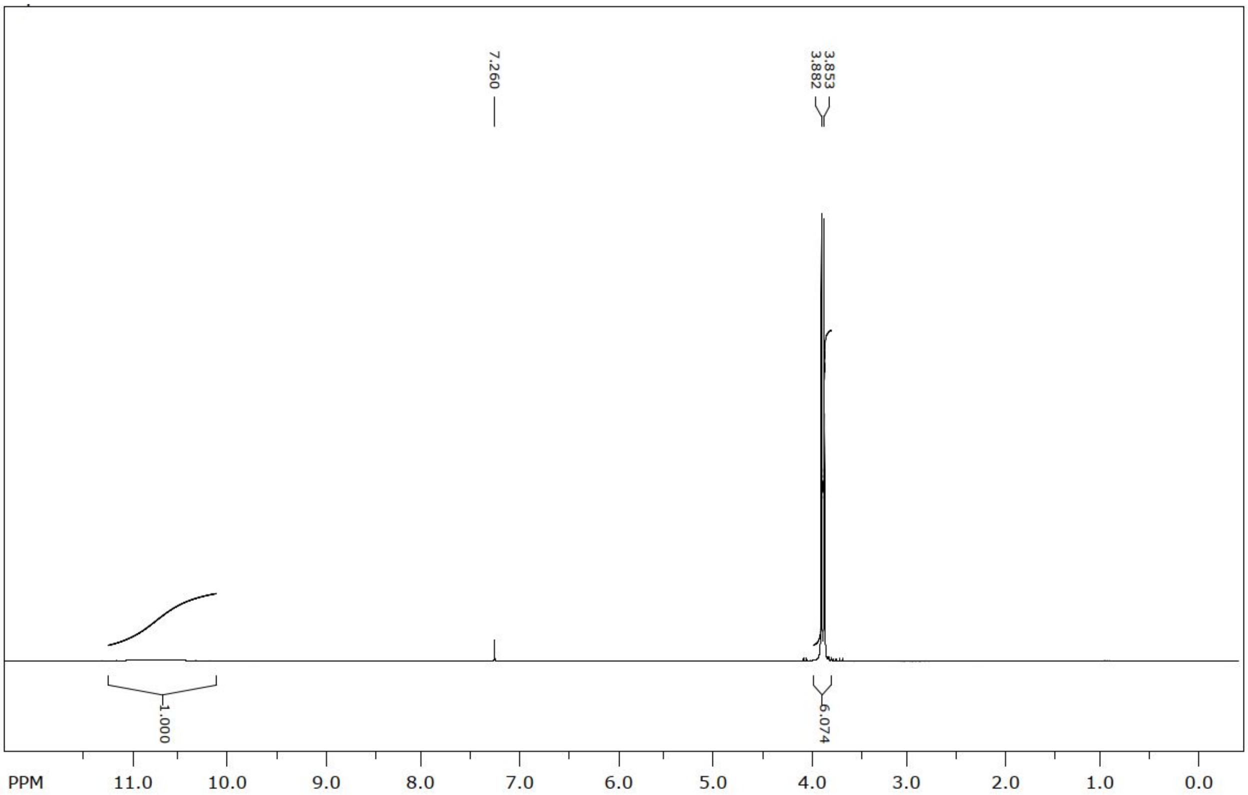

Figure S13. ^{1}H NMR spectrum of H_2**3a** ($CDCl_3$)

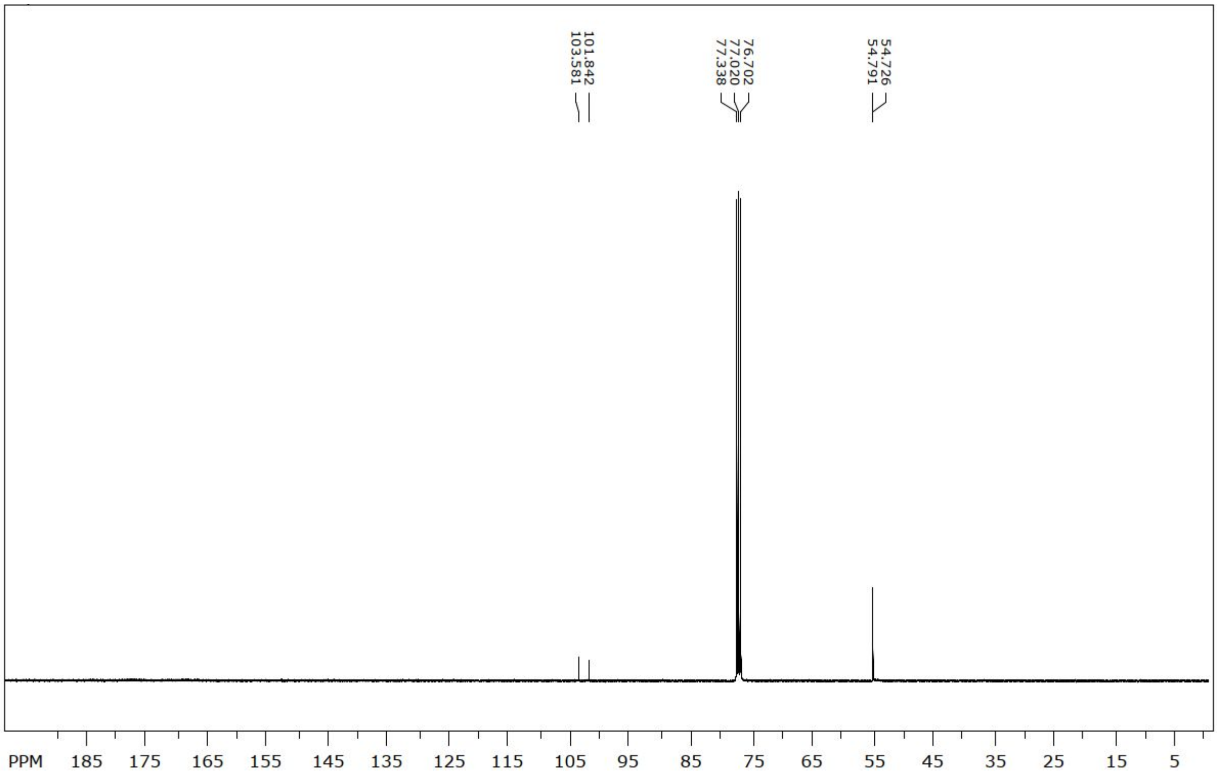

Figure S14. ^{13}C NMR spectrum of H_2**3a** ($CDCl_3$)

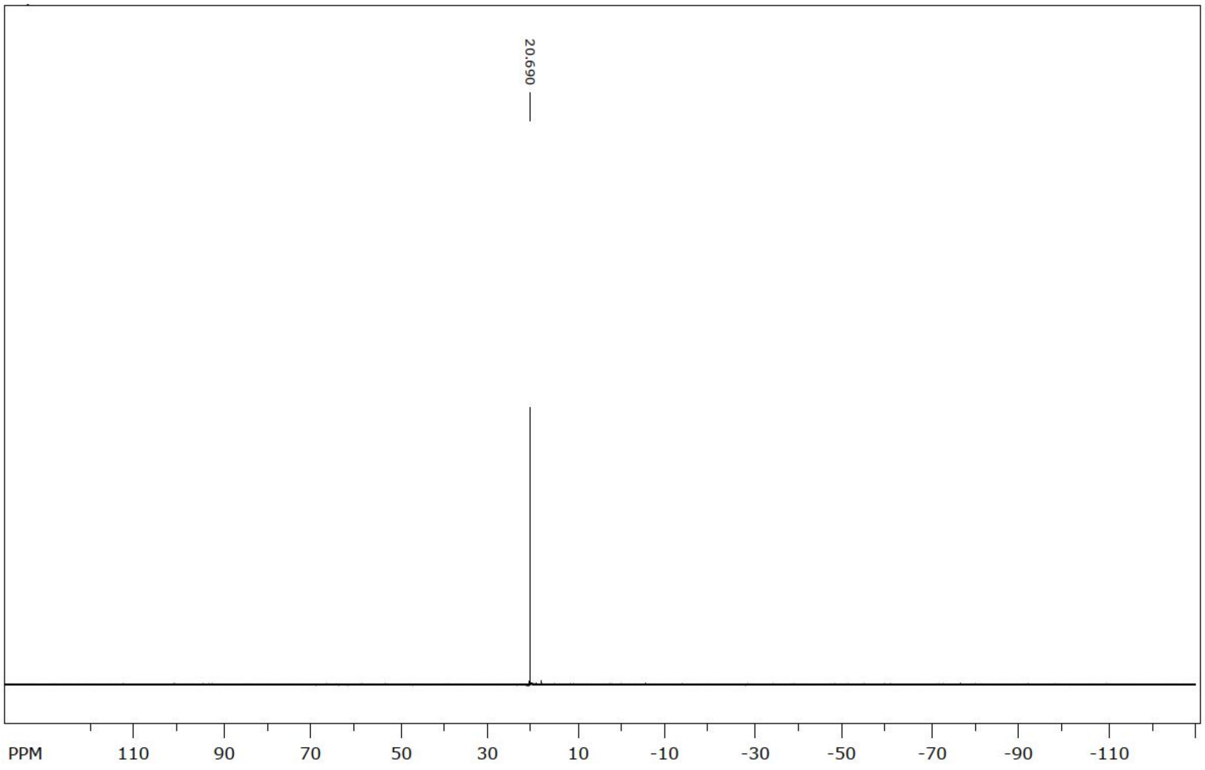

Figure S15. $^{31}P\{H\}$ NMR spectrum of H_2**3a** ($CDCl_3$)

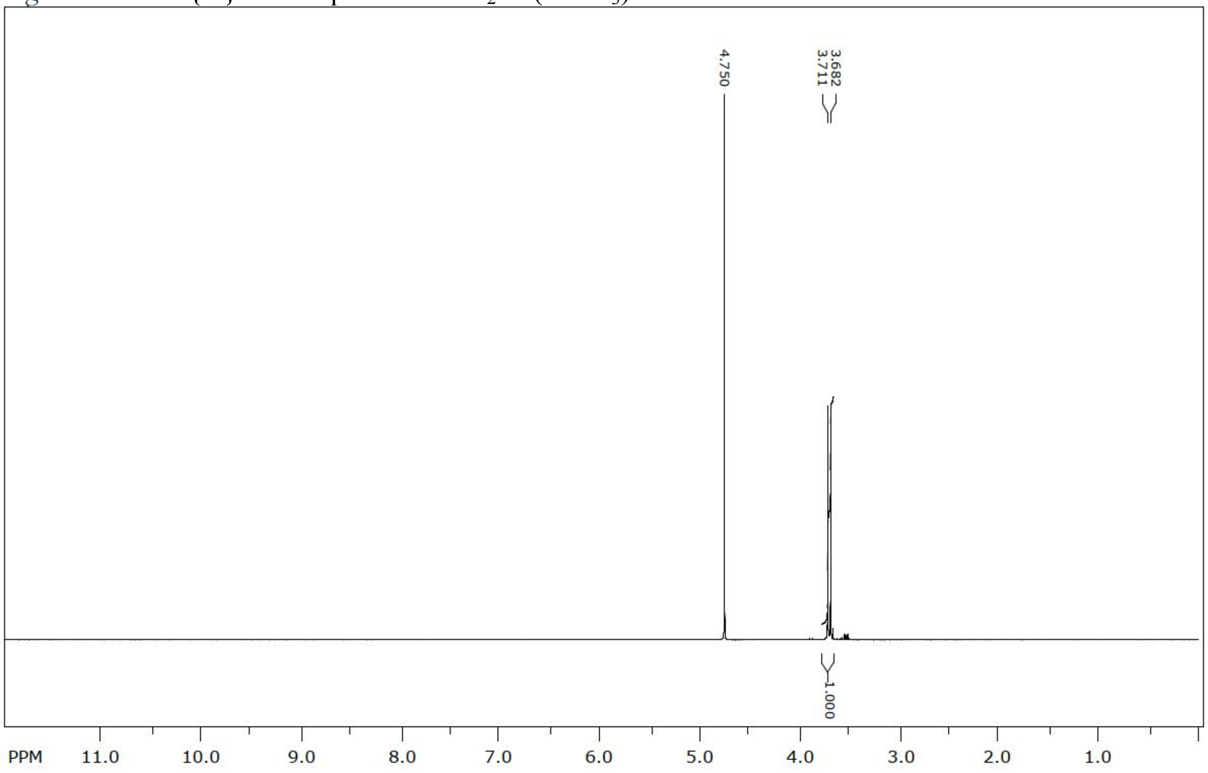

Figure S16. ^1H NMR spectrum of H_2**3a** (D_2O)

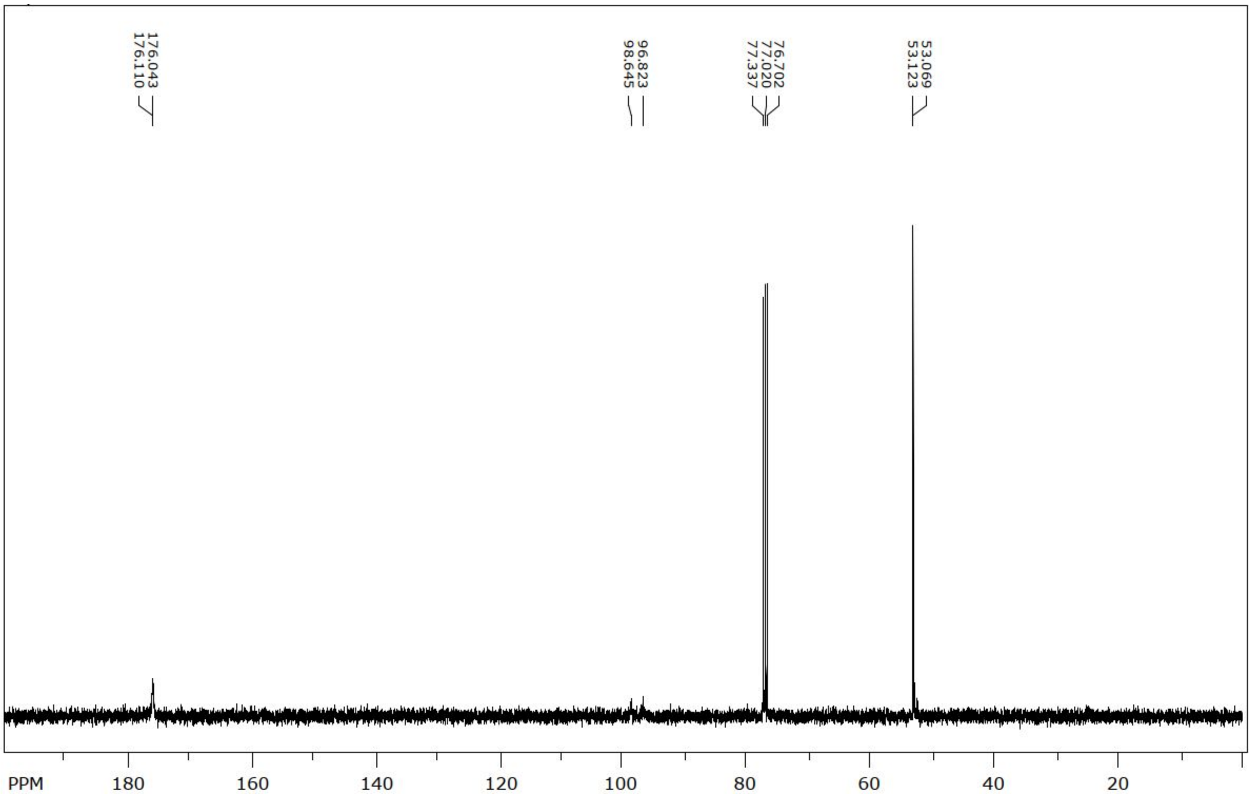

Figure S17. ^{13}C NMR of H_2**3a** (D_2O)

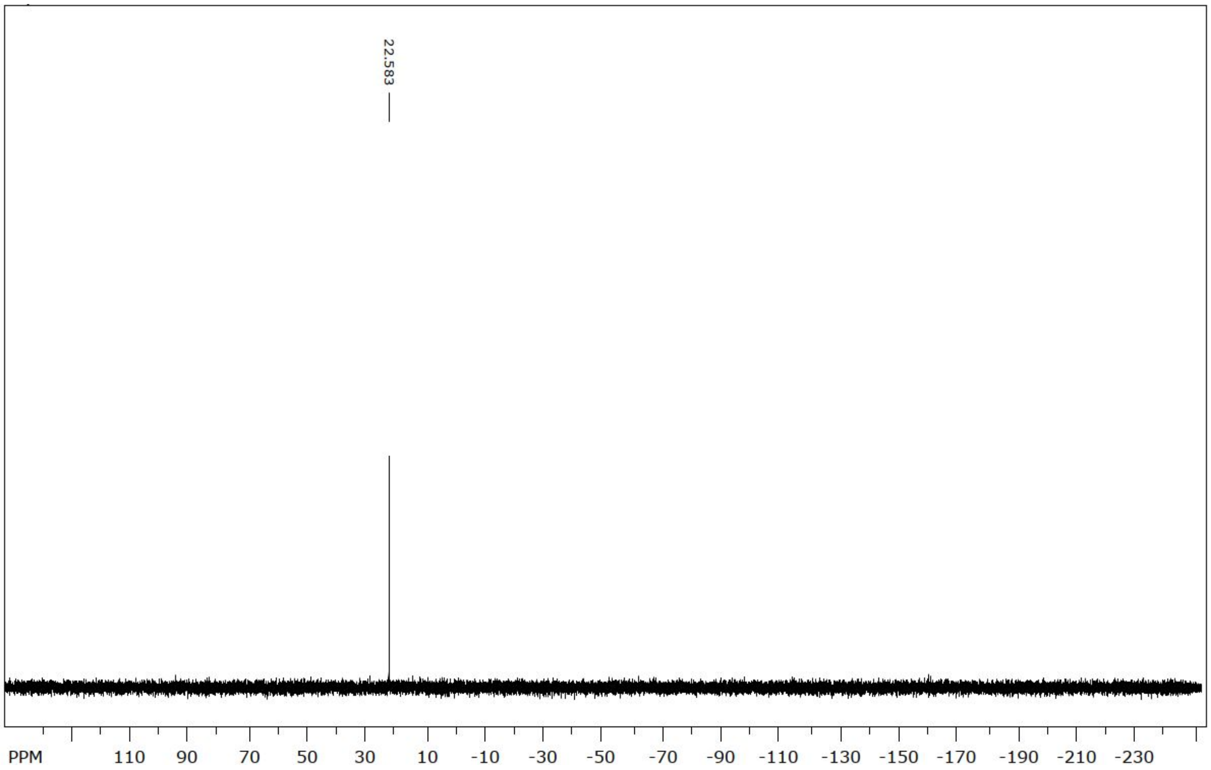

Figure S18. $^{31}P\{H\}$ NMR of H_2**3a** (D_2O)

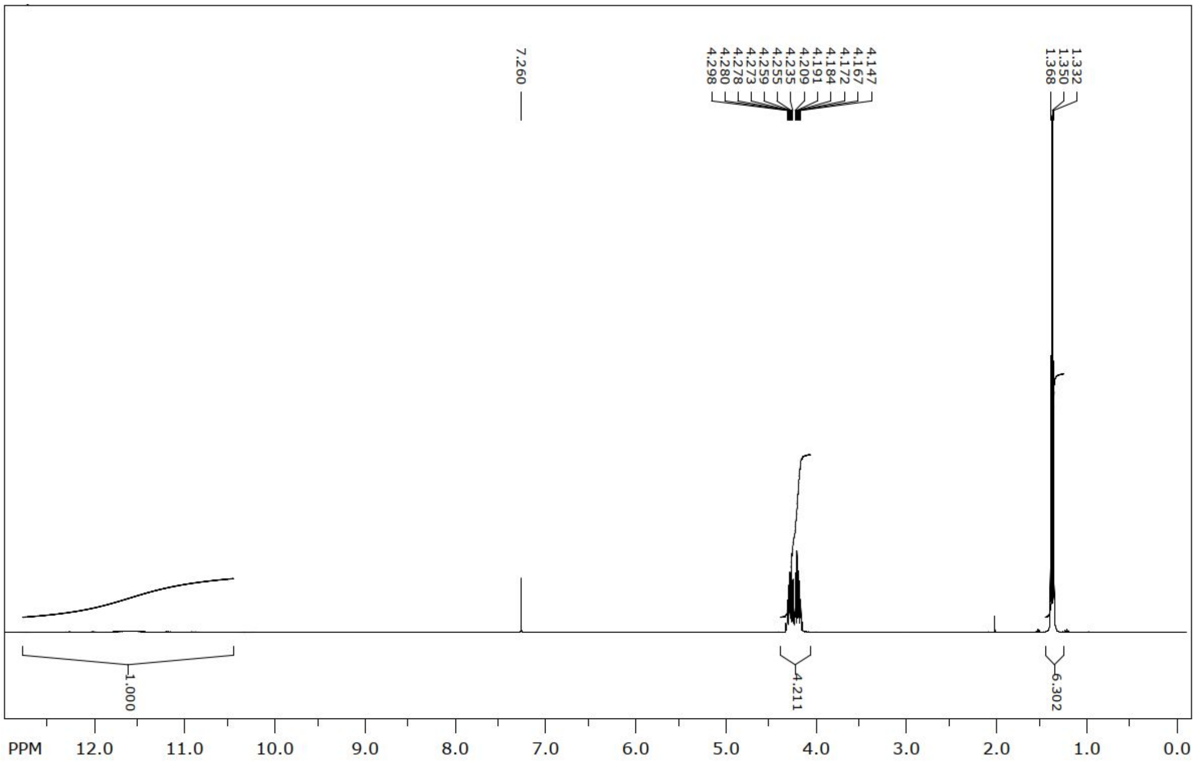

Figure S19. ^{1}H NMR spectrum of H_2**3b** ($CDCl_3$)

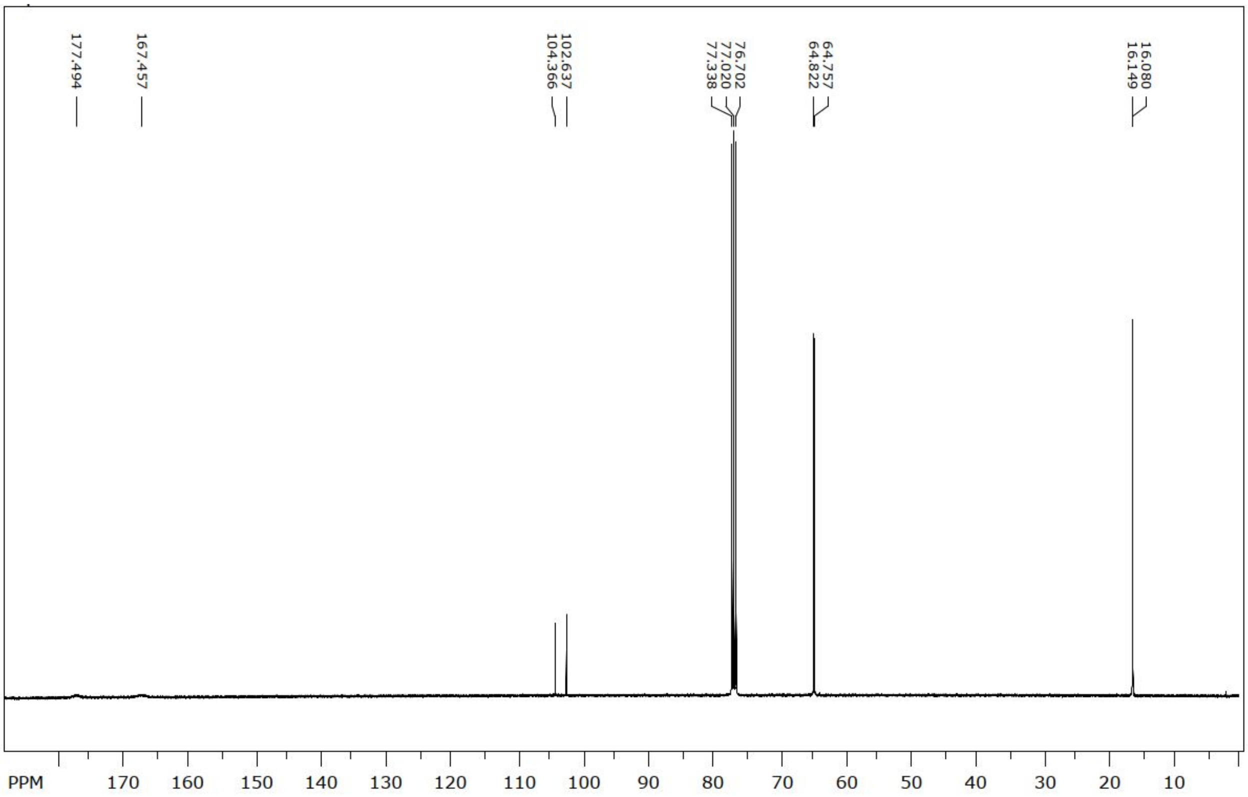

Figure S20. ^{13}C NMR spectrum of H_2**3b** ($CDCl_3$)

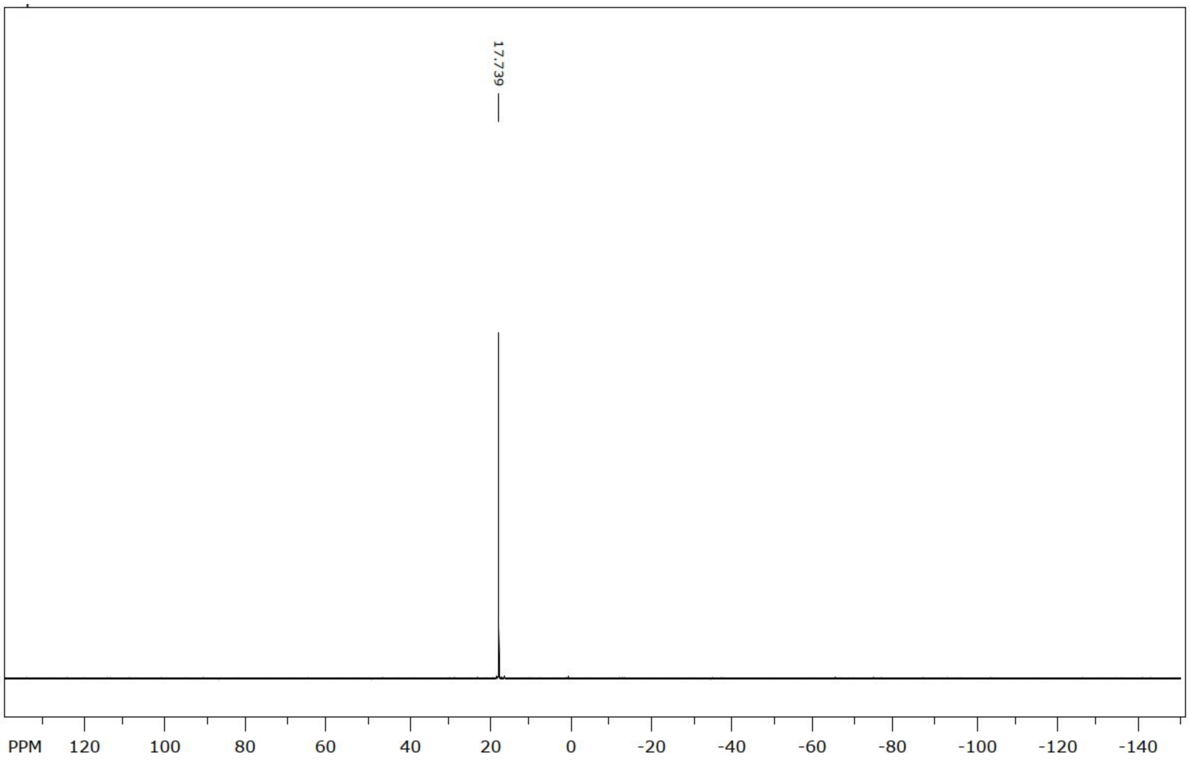

Figure S21. $^{31}P\{H\}$ NMR spectrum of H_2**3b** ($CDCl_3$)

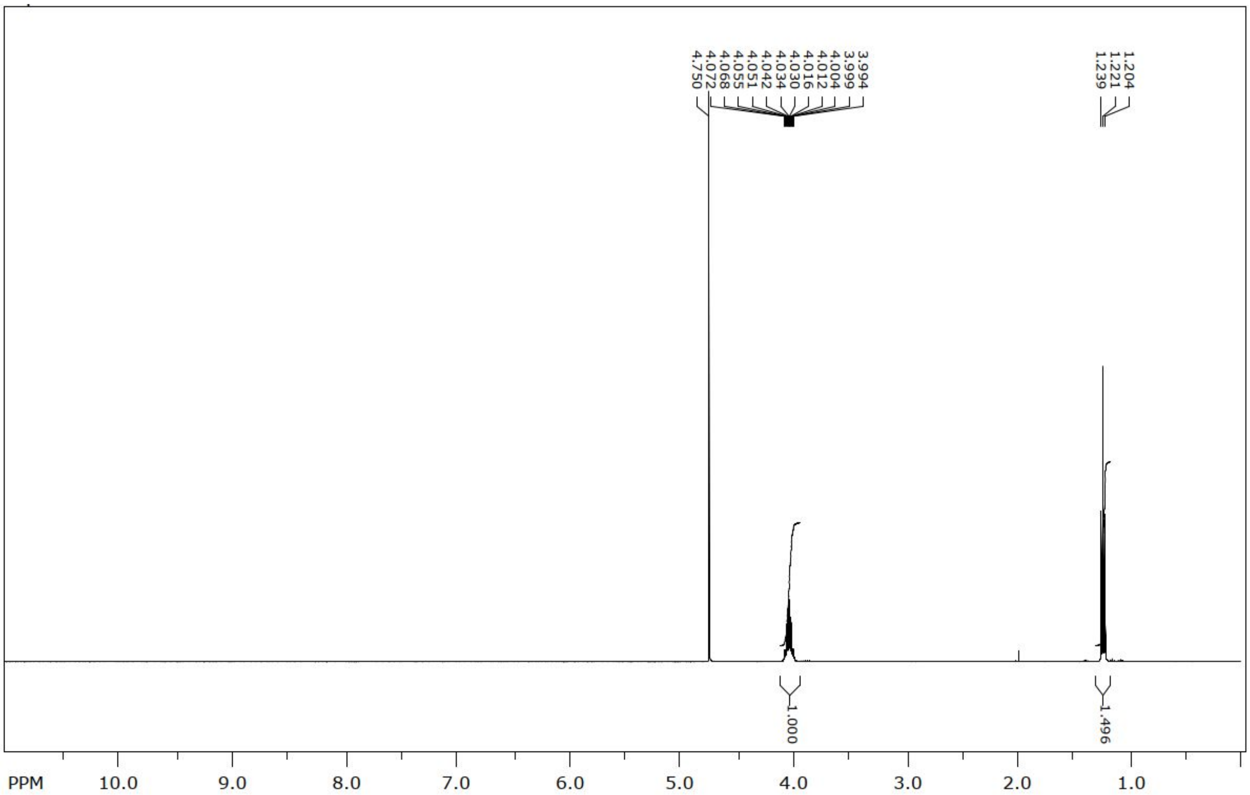

Figure S22. ^1H NMR spectrum of H_2**3b** (D_2O)

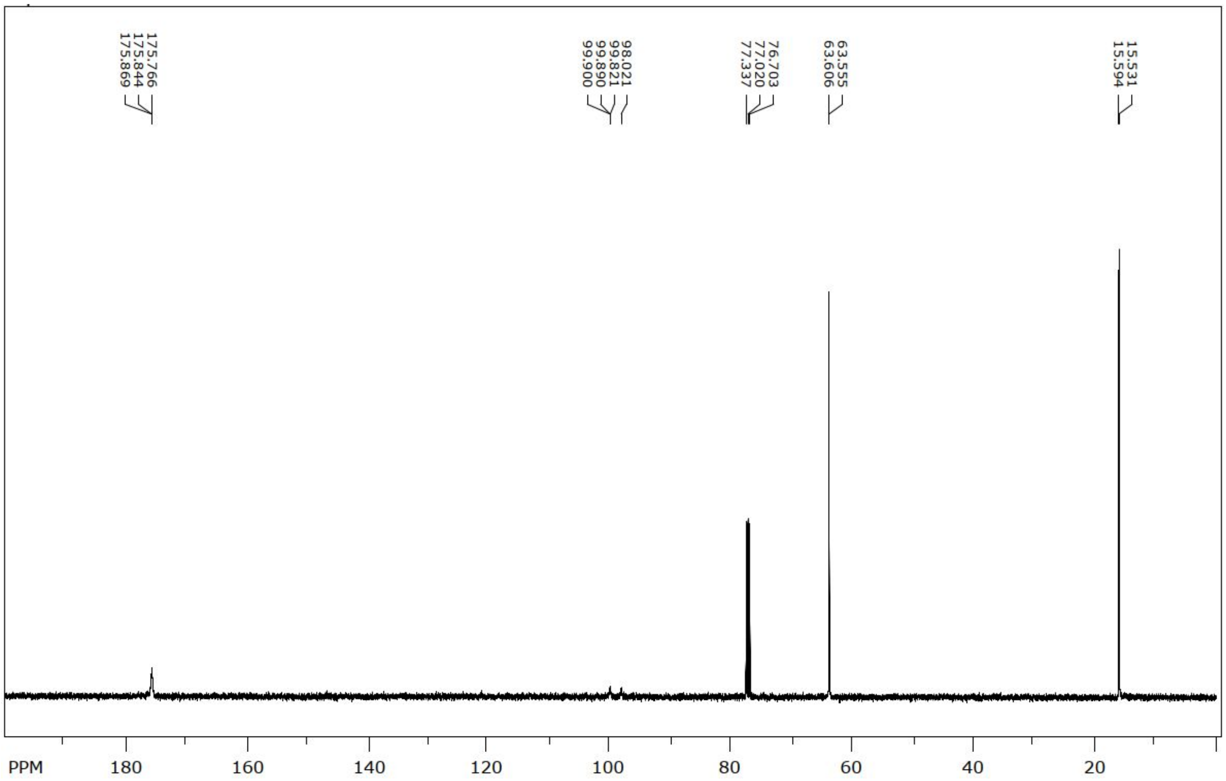

Figure S23. ^{13}C NMR spectrum of H_2**3b** (D_2O)

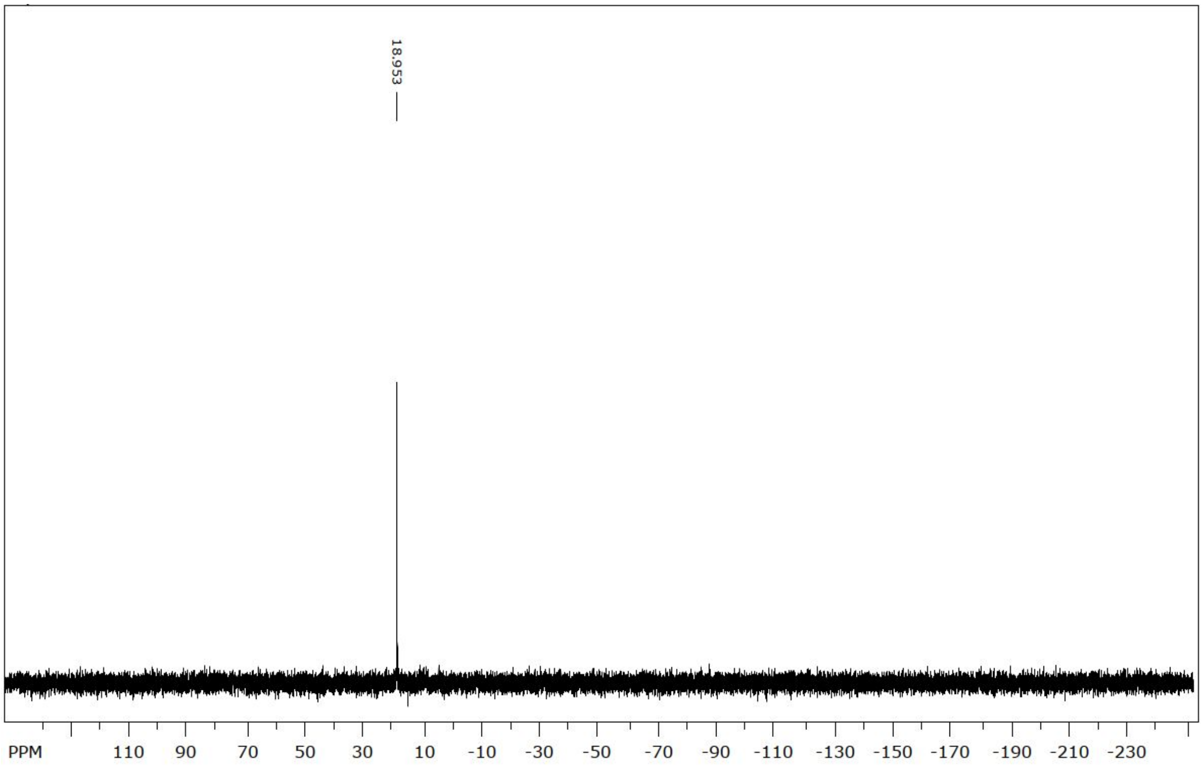

Figure S24. $^{31}P\{H\}$ NMR spectrum of H_2**3b** (D_2O)

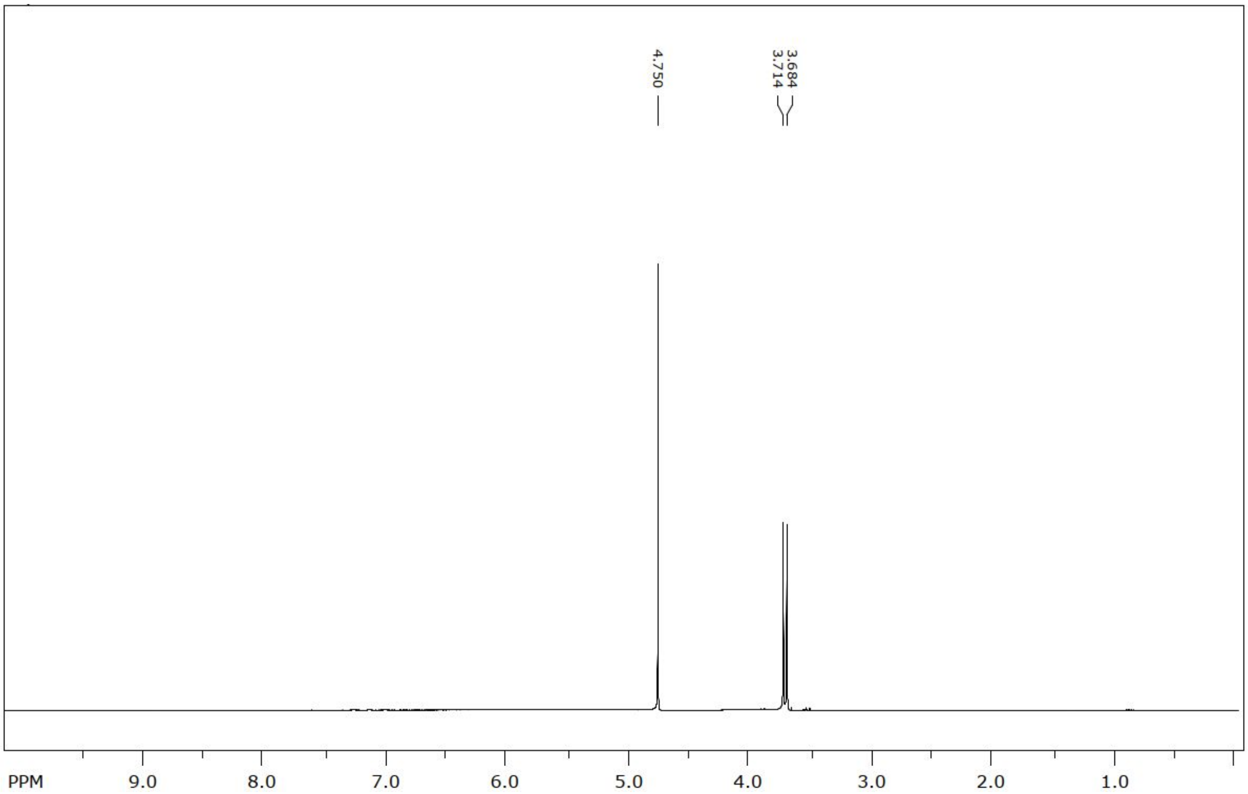

Figure S25. ^{1}H NMR spectrum of $(NH_4)_2$**3a** (in D_2O)

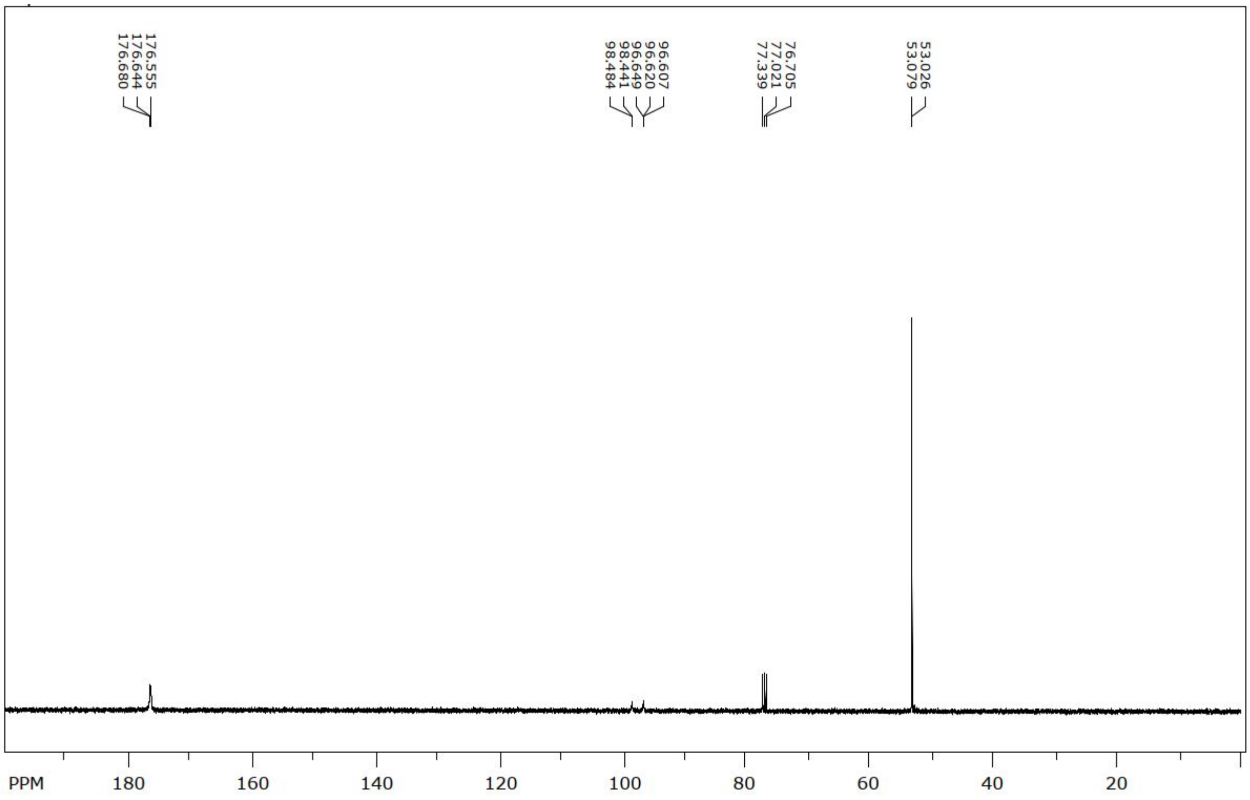

Figure S26. ^{13}C NMR spectrum of $(NH_4)_2$**3a** (in D_2O, with $CDCl_3$ insert (sealed capillary tube))

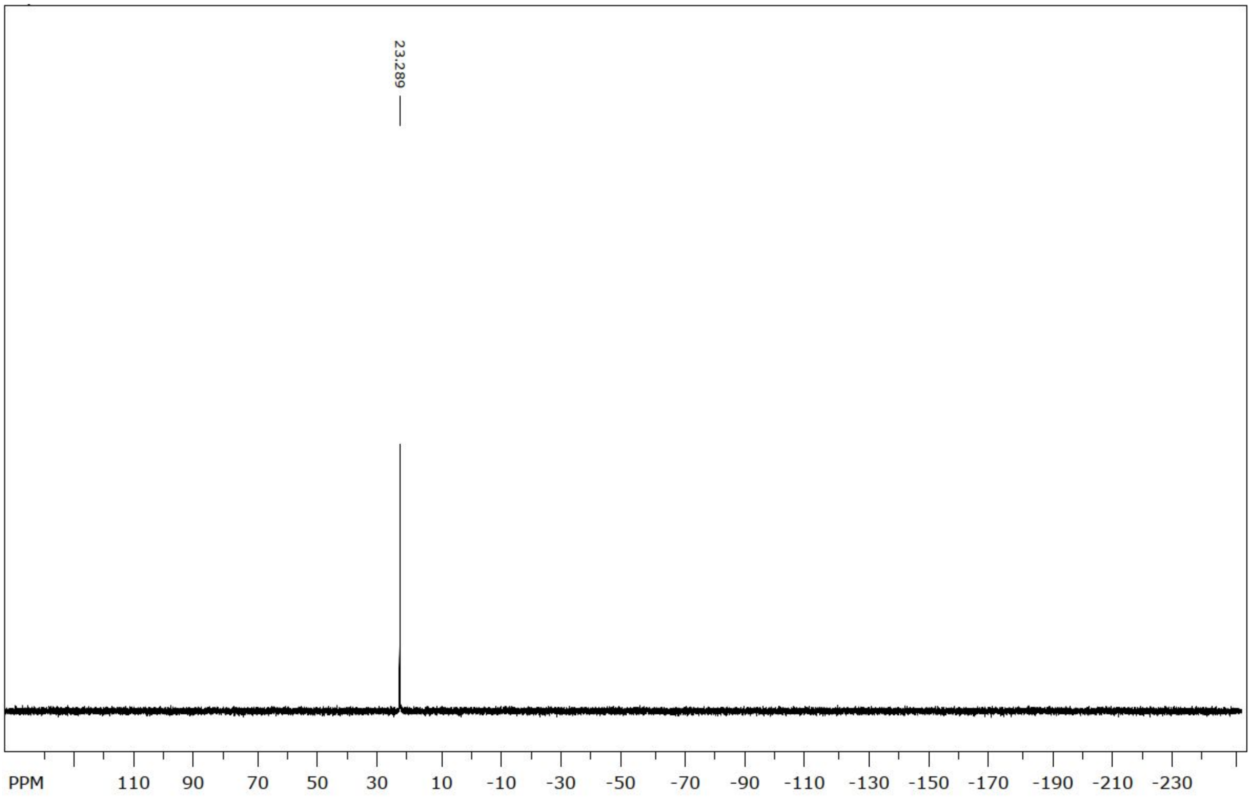

Figure S27. $^{31}P\{H\}$ NMR spectrum of $(NH_4)_2$**3a** (in D_2O)

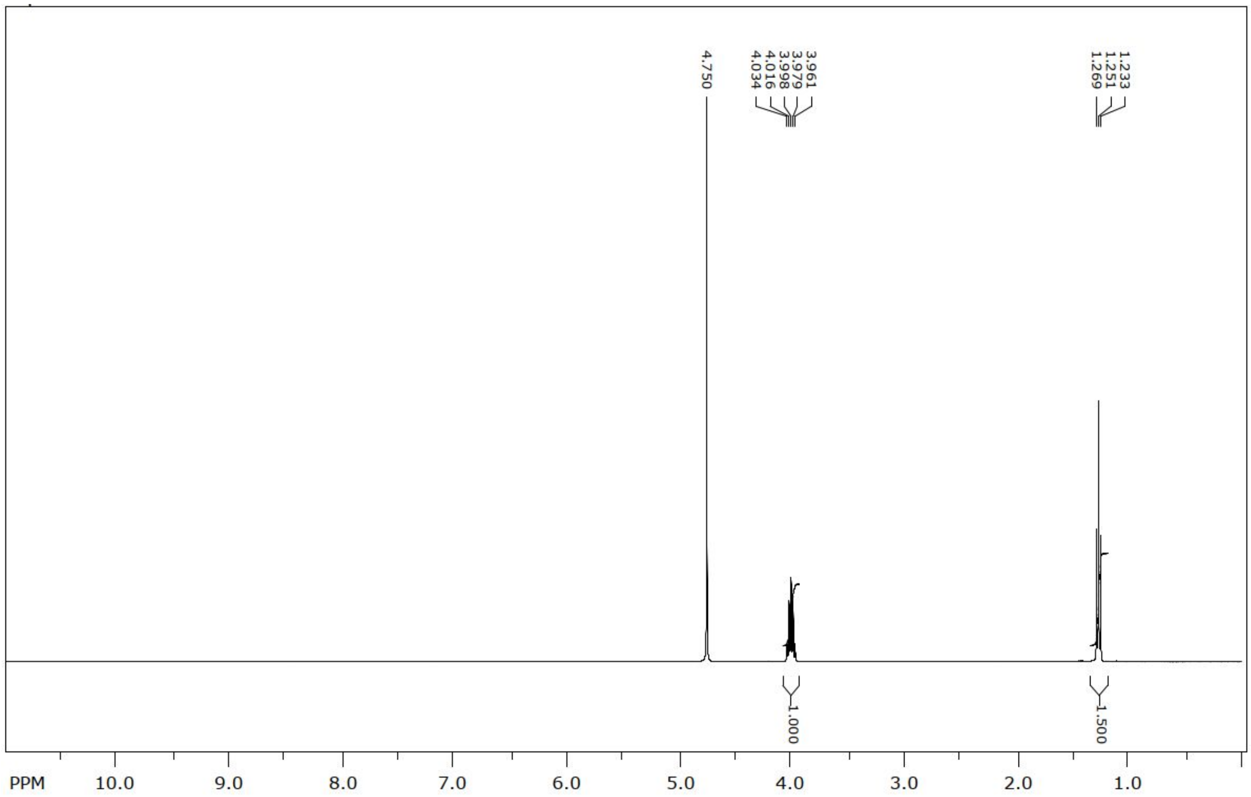

Figure S28. ^{1}H NMR spectrum of $(NH_4)_2$**3b** (in D_2O)

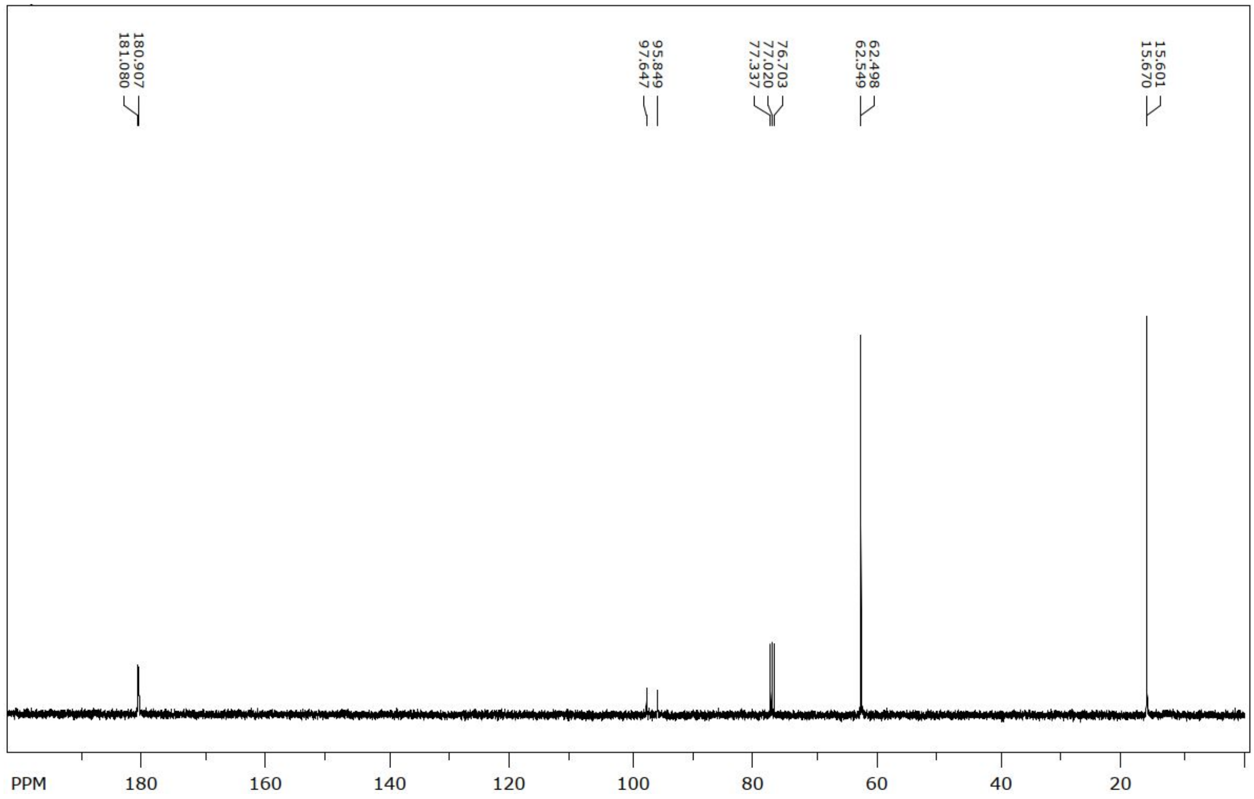

Figure S29. ^{13}C NMR spectrum of $(NH_4)_2$**3b** (in D_2O with $CDCl_3$ insert (sealed capillary tube))

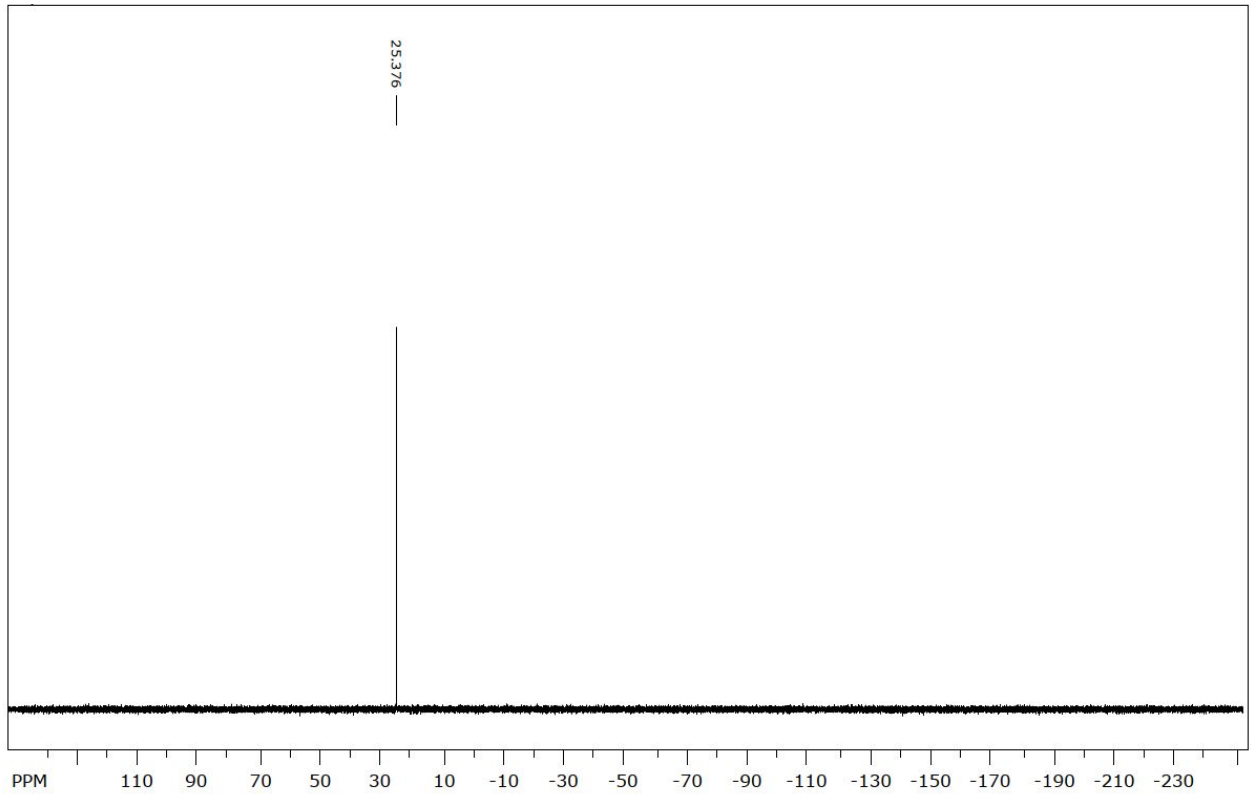

Figure S30. ^{31}P {H}NMR spectrum of $(NH_4)_2$**3b** (in D_2O)

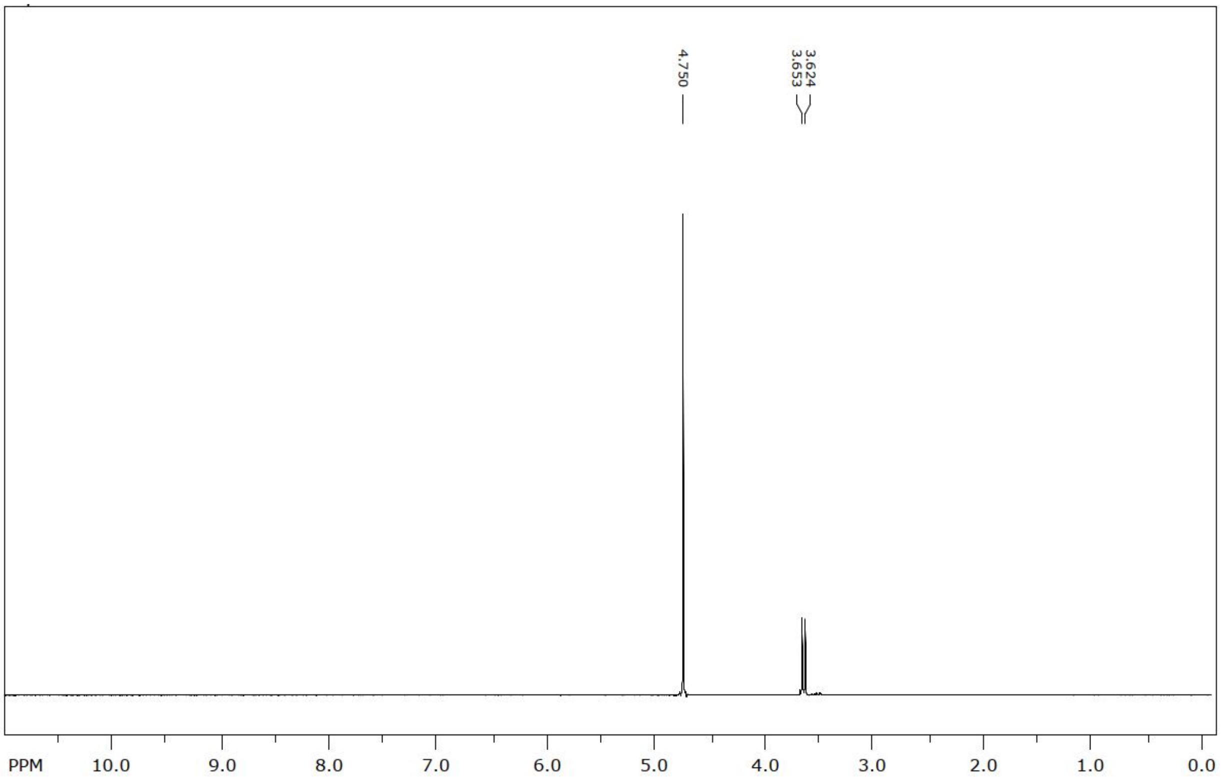

Figure S31. ^{1}H NMR spectrum of Li_2**3a** (in D_2O)

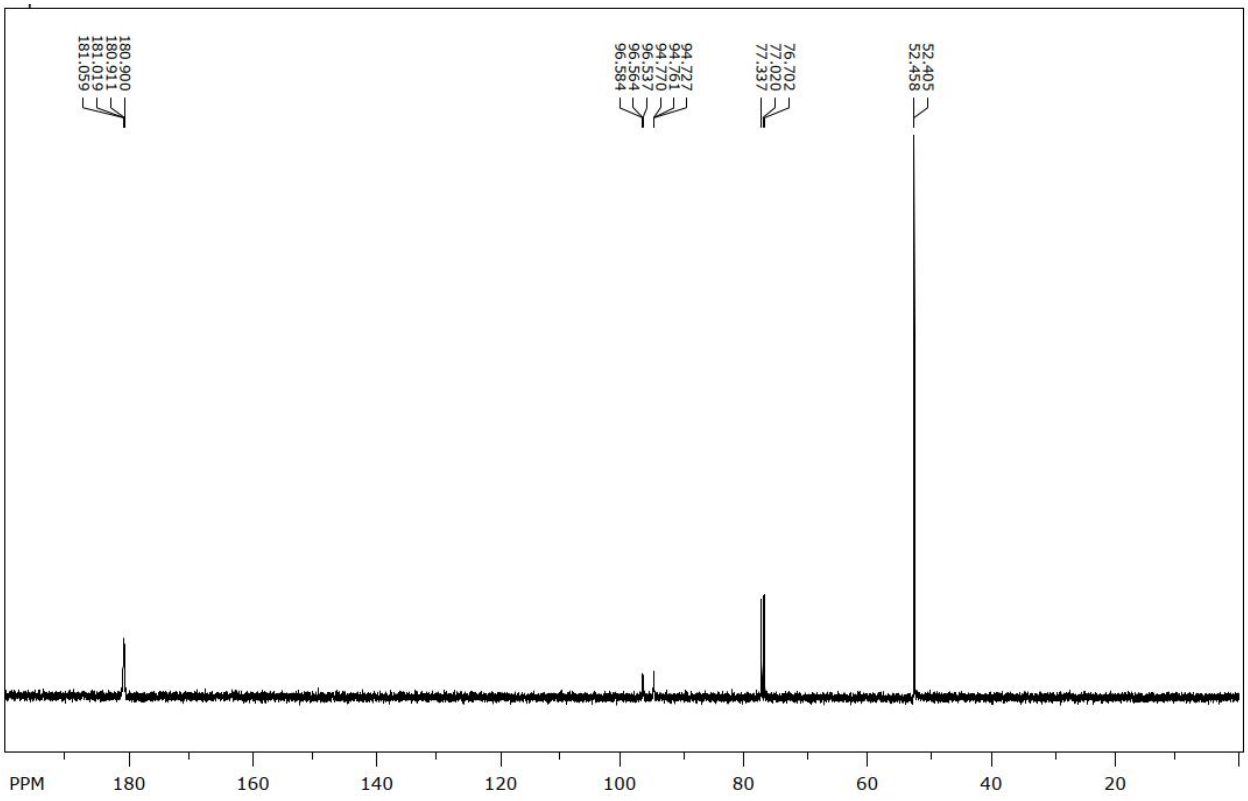

Figure S32. ^{13}C NMR spectrum of Li_2**3a** (in D_2O with $CDCl_3$ insert (sealed capillary tube))

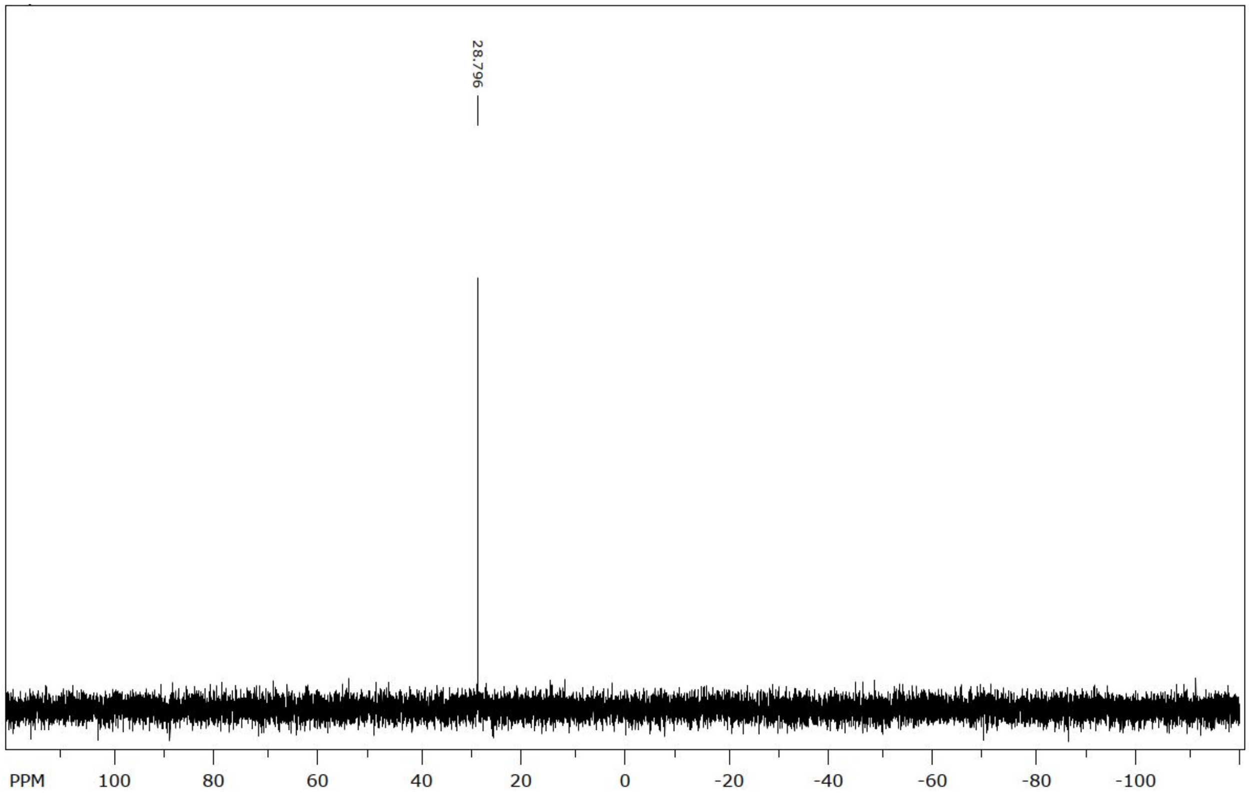

Figure S33. ^{31}P{H} NMR spectrum of Li$_2$**3a** (in D_2O)

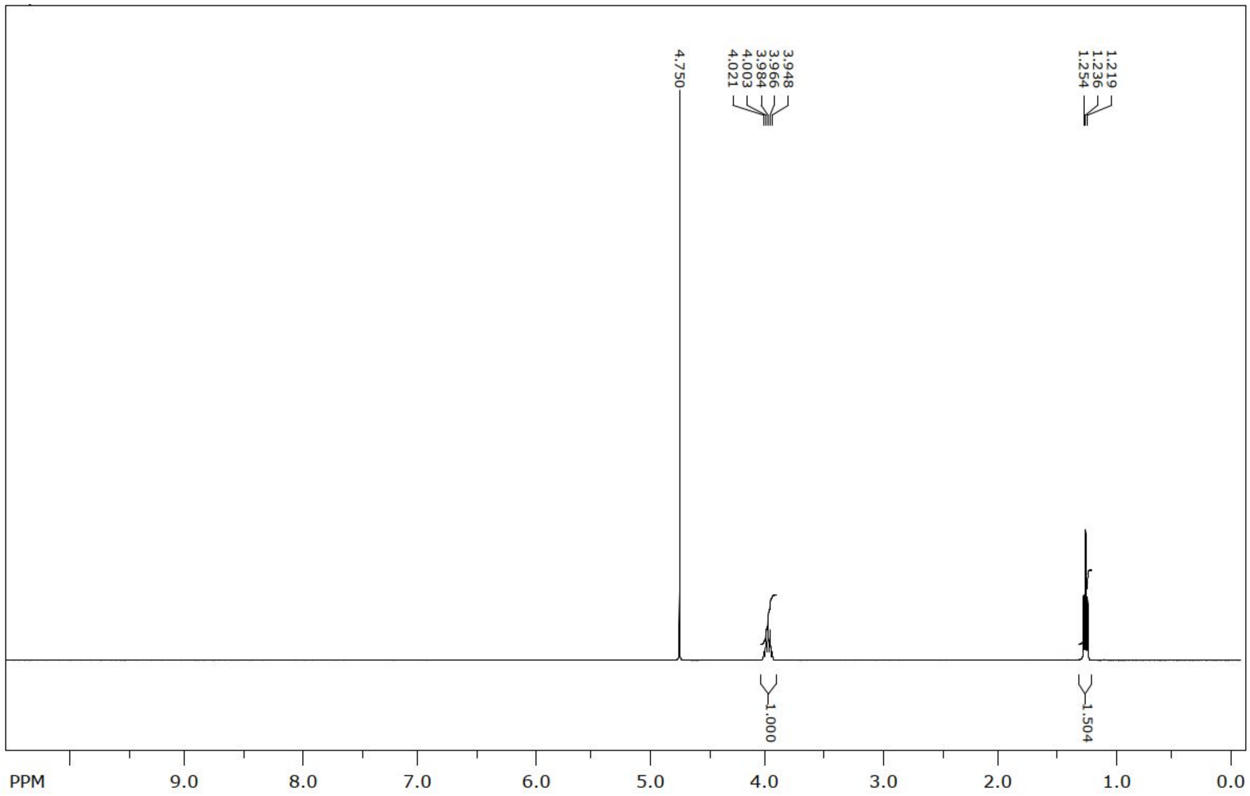

Figure S34. ^{1}H NMR spectrum of Li$_2$**3b** (in D_2O)

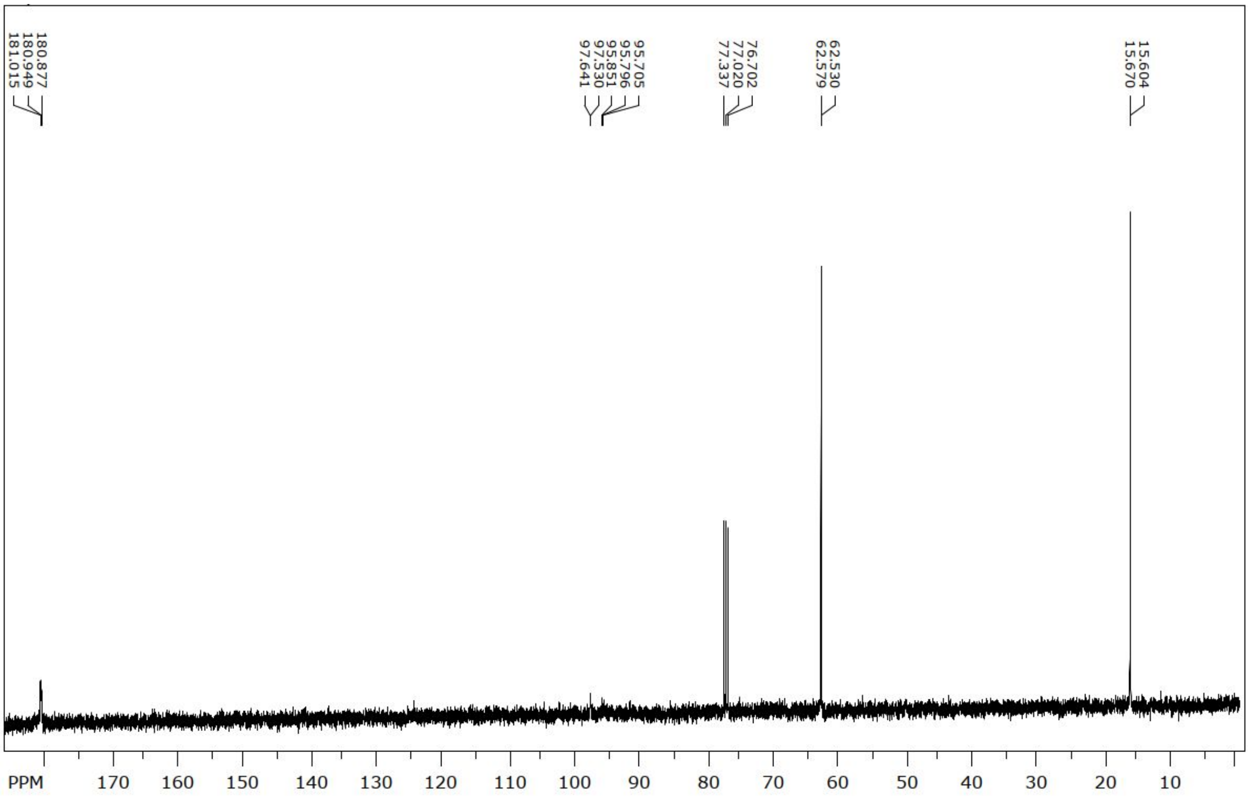

Figure S35. ^{13}C NMR spectrum of Li_2**3b** (in D_2O with $CDCl_3$ insert (sealed capillary tube))

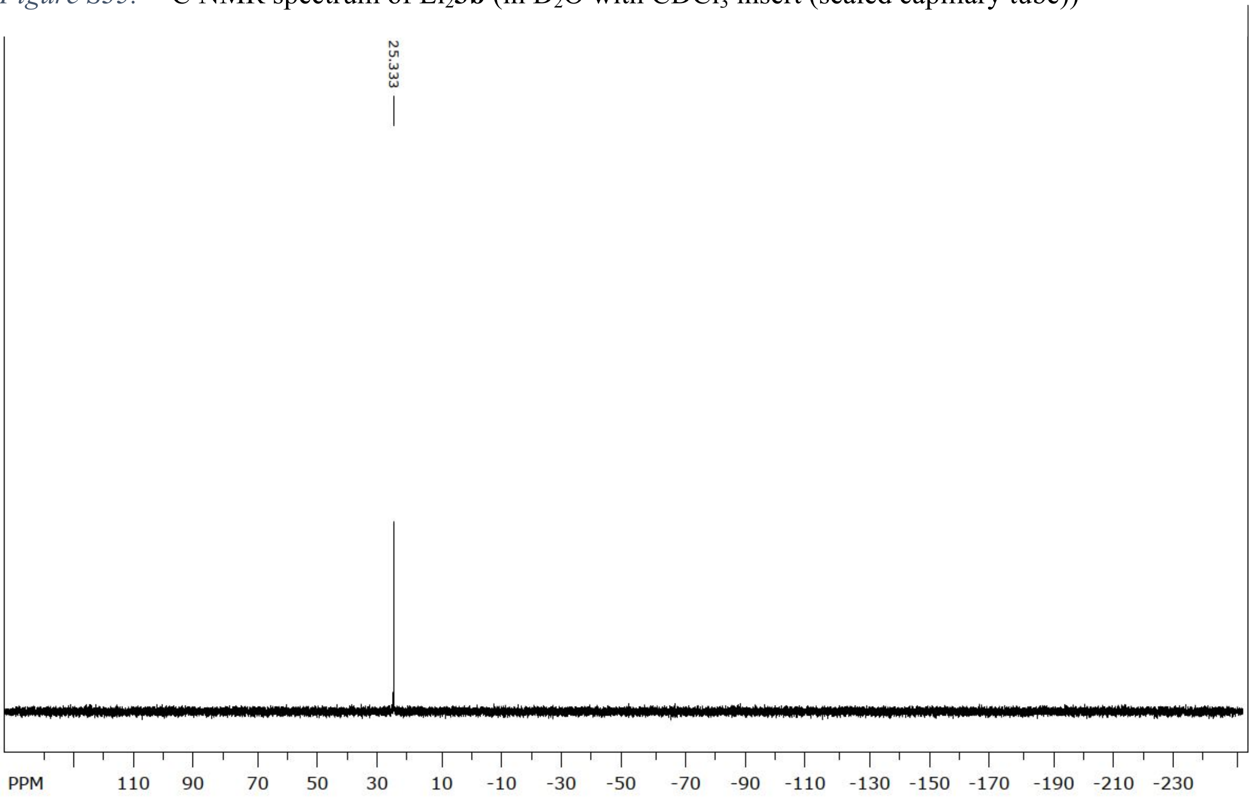

Figure S36. $^{31}P\{H\}$ NMR spectrum of Li_2**3b** (in D_2O)

Table S1. General crystallographic data for 2a, H_2**3a,** H_2**3b,** $(NH_4)_2$**3a**$\cdot H_2O$, $(NH_4)_2$**3b**$\cdot 2H_2O$, Li_2**3a**$\cdot 2H_2O$, and Li_2**3b**

	**2a**	H_2**3a**	H_2**3b**	$(NH_4)_2$**3a**$\cdot H_2O$	$(NH_4)_2$**3b**$\cdot 2H_2O$	Li_2**3a**$\cdot 2H_2O$	Li_2**3b**
Chemical formula	$C_{10}H_{14}Cl_2O_8P_2$	$C_{10}H_{14}O_{10}P_2$	$C_{14}H_{22}O_{10}P_2$	$C_{10}H_{22}N_2O_{11}P_2$	$C_{14}H_{32}N_2O_{12}P_2$	$C_{10}H_{16}Li_2O_6P_2$	$C_{14}H_{20}Li_2O_{10}P_2$
Formula Mass	395.06	356.16	412.27	408.24	482.36	404.06	414.12
Crystal system	orthorhombic	monoclinic	triclinic	triclinic	monoclinic	tetragonal	monoclinic
Space group	Pbca	P 21/c	P -1	P -1	P 21/n	P 41 21 2	C2/c
a/Å	6.9231(5)	9.1887(9)	5.5027(6)	8.0275(4)	12.2174(3)	11.52950(10)	24.776(3)
b/Å	13.8740(10)	9.6177(7)	9.1015(9)	10.4259(7)	13.7492(3)	11.52950(10)	5.3951(5)
c/Å	16.6134(11)	16.7346(13)	9.8565(7)	12.1943(5)	14.5411(3)	12.8329(2)	14.0757(15)
α/°	90	90	92.302(7)	101.250(5)	90	90	90
β/°	90	91.807(8)	104.226(8)	106.994(4)	108.814(2)	90	94.851(10)
γ/°	90	90	97.230(9)	105.104(5)	90	90	90
Unit cell volume/Å^3	1595.74(19)	1478.2(2)	473.40(8)	900.73(9)	2312.10(9)	1705.87(4)	1874.7(4)
Temperature/K	299(2)	297(2)	299(2)	110(2)	297(2)	110(2)	297(2)
Density (g/cm^3)	1.644	1.6	1.446	1.505	1.386	1.573	1.503
Radiation wavelength	0.71073Å	0.71073Å	1.54184Å	0.71073Å	0.71073Å	1.54184Å	1.54184Å
Formula units per unit cell, Z	4	4	1	2	4	8	8
Absorption coefficient, μ/mm^{-1}	0.642	0.343	2.550	0.299	0.247	2.891	2.575
Reflections measured	2705	2822	1423	5821	7680	1674	1426
Independent reflections	2225	1732	1311	5355	6274	1673	1224
R_{int}	0.0372	0.0873	0.0421	0.0186	0.0466	0.0206	0.0472
Final R_1 values ($I > 2\sigma(I)$)	0.0363	0.0818	0.048	0.0291	0.0482	0.0279	0.0721
Final $wR(F^2)$ values ($I > 2\sigma(I)$)	0.1023	0.1791	0.1298	0.0773	0.1372	0.0742	0.1888
Final R_1 values (all data)	0.0457	0.1113	0.0508	0.0323	0.059	0.0279	0.0778
Final $wR(F^2)$ values (all data)	0.1083	0.1871	0.1328	0.0794	0.1482	0.0742	0.1997
Goodness of fit on F^2	1.055	1.085	1.067	1.032	1.04	1.086	1.096
CCDC number	2356578	2356579	2356580	2356582	2356584	2356581	2356583
